# Supramolecular Effects of Alkyl Sulfonates in Silver Nanocrystal Synthesis

**DOI:** 10.1021/acsnanoscienceau.5c00121

**Published:** 2025-12-04

**Authors:** Nicola L. Myers, Clara M. Hansen, Clare N. Hermanson, Keenan Tiddle, Grant Didway, Noah Kaplan, Helen C. Larson, Catherine C. Bodinger, Brandi M. Cossairt, Steven M. Hughes, Mark P. Hendricks

**Affiliations:** † Department of Chemistry, 8233 Whitman College, Walla Walla, Washington 99362, United States; ‡ Department of Chemistry, 7284 University of Washington, Seattle, Washington 98195, United States; § Department of Chemistry, 7037 Roanoke College, Salem, Virginia 24153, United States

**Keywords:** silver nanocrystals, nanocrystal synthesis, nanocrystal surface chemistry, surfactants, supramolecular chemistry

## Abstract

While cationic surfactants such as hexadecyltrimethylammonium bromide (CTAB) are ubiquitous in the synthesis of noble metal nanocrystals, anionic surfactants are rarely used. This work explores the addition of sodium alkyl sulfonates with chain lengths ranging from one to eight carbons to a silver nanoplatelet reaction. Short-chain sulfonates comprised of one to four carbons show little effect on the nanocrystal synthesis, but alkyl sulfonates comprised of five or more carbons at concentrations above 1 mM have a pronounced effect on the absorbance of the nanocrystals, causing a blue-shift in the wavelength of maximum absorbance (λ_max_) from approximately 800 to 400 nm as the sulfonate concentration is increased to 7 mM. Higher concentrations of sulfonate result in a subsequent red-shift of the peak. Investigation into the possible formation mechanisms responsible for this synthetic control revealed the absence of sulfonate micelles under the reaction conditions. Instead, we hypothesize that sulfonate bilayers are nucleated around the silver nanocrystals at concentrations below the critical micelle concentration and interact with either the citrate ligands or the silver surface to influence nanocrystal morphology, and thus absorbance. Strikingly, the addition of long-chain alkyl sulfonates to already-synthesized nanocrystals results in similar changes to the nanocrystal absorbance that occur within seconds, providing further support for the proposal that these effects are related to the surface chemistry of the nanocrystals, which appear to be highly dynamic.

## Graphical abstract

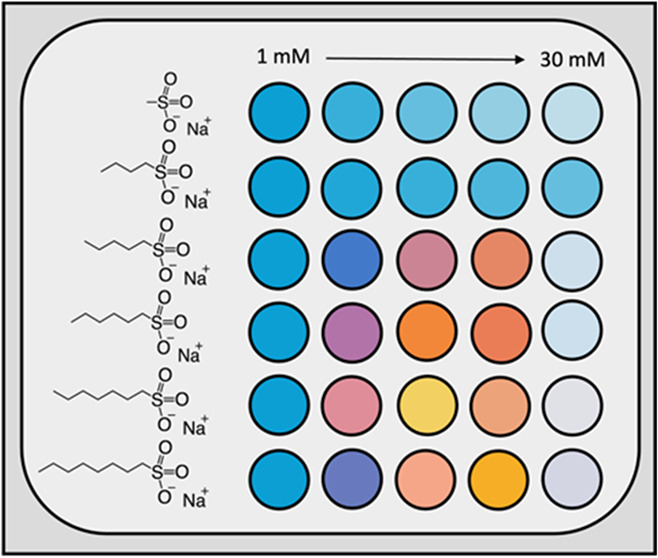

## Introduction

While many sizes and shapes of noble metal nanocrystals are now accessible thanks to significant work in developing modern syntheses,^1–5^ there are reasons to continue exploring new synthetic routes, even beyond expanding the scope of size and shape control. In particular, the synthesis conditions of nanocrystals define the surface chemistry of the particles, and thus producing different surfaces amenable to myriad applications without the need for postsynthetic ligand exchange is a worthy endeavor.^6^ Additionally, new synthetic routes can also shed light on the underlying mechanisms through which the crystals form.^2–5^ Gold nanocrystals generally receive more attention for both their synthesis and applications, likely due to their relative stability compared to silver.^3,7,8^ However, silver demonstrates the strongest localized surface plasmon resonance (LSPR) in the visible region among the noble metals^8^ and also exhibits antimicrobial properties,^9,10^ thus making it an important target of study. The LSPR enhances the light absorption and scattering properties of silver nanocrystals and can be tuned for specific applications by modulating the sizes and shapes of nanocrystals.^1,11^ The development of syntheses that influence the absorption properties and surfaces of plasmonic metal nanocrystals is important for their applications in sensing, imaging, electronics, and catalysis.^1,8,12,13^

Within traditional reductant-driven syntheses of metallic nanocrystals, there are a variety of general methods that can be used to influence the size and shape of the resultant nanocrystals.^14^ One of these is the rate of reduction of the metal ions, which can be influenced by the strength and concentration of the reducing agent, and by the presence of species that compete to be reduced.^14–16^ General reaction conditions, such as reaction temperature, also influence the rate of reduction, but can affect the size and shape of the particles in more complicated ways.^17^ Finally, among the most common methods for influencing the morphology of metallic nanocrystals is through the use of ligands that interact with the surface of the crystals.^2,14,18^ Halide anions, small molecules, surfactants, and polymers are commonly used precursors that interact with the nanocrystal surface. Previous work has shown that silver nanocubes with well-defined edges can be synthesized with the use of halide anions such as chloride or bromide and polymers such as poly­(vinylpyrrolidone) (PVP), which both preferentially bind the {100} facets of nanocrystal surfaces.^19–21^ The small molecule citrate is thought to passivate {111} silver nanocrystal surface facets through electrostatic interactions between the negatively charged carboxylate groups and the positively charged silver surface.^22,23^ Citrate is commonly used in prismatic plate syntheses, though other carboxylate anions have also been found to stabilize silver nanoplatelets, the dimensions of which can be controlled through the addition of ligands with hydroxyl groups.^2,24^ Halide anions, such as bromide, can also be used to tune the size of silver nanoplatelets by preferentially binding to {100} facets.^22^

Surfactants provide an additional route for ligand influence of nanocrystal morphology.^18,26–29^ Surfactants, amphiphilic molecules that self-assemble into supramolecular structures via intermolecular forces,^30^ are known to interact with nanocrystal surfaces.^18,26–29^ The most commonly used surfactant in noble metal nanocrystal synthesis is hexadecyltrimethylammonium bromide (CTAB), with a hydrophobic chain of 16 carbons and a positively charged hydrophilic quaternary ammonium headgroup.^3,26,28,29,31,32^ This is coupled with a bromide counterion, thus providing two distinct ligand motifs from the same molecule. Both are critical to the formation of gold nanorods in seed-mediated syntheses, as bromide by itself or the surfactant with a chloride counterion fail to reproduce the nanorod structures.^33^ The hexadecyltrimethylammonium molecule is thought to interact with the gold nanorods in a bilayer structure, the packing of which is dependent on surface curvature.^34–36^ It has also been shown that the length of the chain influences the aspect ratio of the nanorods, with chains of 12 or more carbons necessary to produce significant anisotropy.^31,32,34^

While cationic surfactants such as CTAB are ubiquitous in the synthesis of noble metal nanocrystals, anionic surfactants have received far less attention, so we were interested in exploring their supramolecular behavior in silver nanocrystal syntheses. Sodium dodecyl sulfate (SDS) is one of the few commonly used anionic surfactants, but other alkyl sulfates are rather uncommon.^18,27,28,37,38^ We have opted to focus on alkyl sulfonates, which differ from the sulfates with the alkyl chain bonding directly to the sulfur. Alkyl sulfonates should not interact strongly with silver ions. In the only study we could find that measured the binding strength of a surfactant sulfonate with silver, the log­(K) of the stability constant of phenylsulfonate and para-methoxyphenylsulfonate with silver ions was measured as −0.04 and −0.12, respectively.^39^ In cases where self-assembled monolayers (SAMs) are formed between alkylthiols and metal surfaces, a proposed decomposition pathway is the oxidation of the thiol to the associated sulfonate, which would then desorb from the surface due to the weak binding of sulfonates to metals.^40,41^ While sulfonates should not bind strongly with the silver, other mechanisms could nonetheless lead to interactions; even the details of how and why the quaternary ammonium headgroup of hexadecyltrimethylammonium interacts with metal nanocrystal surfaces remain unresolved.^26,31^ The sulfonate headgroup is also biologically compatible,^42–45^ which could make the alkyl sulfonates a potentially interesting target when coupled with the antimicrobial properties of silver.^9,10^ It should be noted that sulfonate polymers have been used in conjunction with metal nanoparticles, but rather than being used directly in the syntheses, they are used to modulate the charge of the nanoparticles through a polyelectrolyte layer-by-layer wrapping approach and we therefore believe the work described herein is distinct.^42,45–47^

In this work, a well-established silver nanoprism synthesis was adapted to investigate the effect of small changes to the molecular structure of sulfonate surfactants on nanocrystal absorbance.^48^ The aqueous and room temperature nature of the synthesis provides optimal conditions for studying the complexity of supramolecular systems. Previous work has shown nanoprism size is sensitive to the addition of bromide, which was used at concentrations 3 to 4 orders of magnitude lower than the silver precursor concentration.^22^ With the use of an automated system, we explored the effects of sodium alkyl sulfonates with varying chain lengths across a concentration range of 6 orders of magnitude. We find that alkyl sulfonates with five or more carbons strongly influence the absorbance of silver nanocrystals above concentrations of 1 mM that remain well below the critical micelle concentration of the alkyl sulfonates. Similar effects are observed whether the longer-chain sulfonate is included in the synthesis or added after the nanocrystals have formed, where changes occur within seconds. Short-chain alkyl sulfonates do not show similar effects.

## Results and Discussion

In 2005, Métraux and Mirkin developed an aqueous, room-temperature, seedless silver nanoprism synthesis that balanced the concentrations of hydrogen peroxide as an oxidative etchant and sodium borohydride as the reducing agent to control the size, and therefore absorption, of the nanoprisms.^48^ Their reaction used both poly­(vinylpyrrolidone) and citrate as ligands, which when coupled with the hydrogen peroxide, effectively formed triangular platelets with tailorable thickness. This work was later adapted by Frank et al., who removed the poly­(vinylpyrrolidone) and optimized the reaction at a constant sodium borohydride concentration to instead use bromide ion concentration to influence the size and absorption of the nanoprisms.^22^ The bromide ions are thought to attach to silver ions on the nanoparticle surface and slow growth, resulting in smaller nanocrystals at higher bromide concentrations.^22^ While the synthesis developed by Frank et al., like some metallic nanocrystal syntheses,^5,49–52^ was optimized to work without stirring, we find that our reaction is highly sensitive to stirring (Figure S1 in Supporting Information).^53^

We adapted this synthesis to explore the role of anionic surfactants in silver nanocrystal synthesis, removing the bromide and instead adding a sodium alkyl sulfonate of variable carbon chain length to the reaction. For control reactions, the equivalent concentration of sodium nitrate was added to match the ionic strength of the sulfonate using ions that are already present in the reaction solution at relatively high concentrations. As described in the experimental section, we developed our reaction to run at a 200 μL scale in common plastic 96-well microplates using a liquid-handling robot and integrated UV–vis plate reader to make and measure the nanocrystal samples, respectively (Figure S2 in Supporting Information).^54^ We utilize the shake function of the plate reader to mix reagents during a reaction. In a standard reaction, reagents were added in the following order to obtain the final reaction concentrations as listed: milli-Q water (if needed), silver nitrate (0.09375 mM), trisodium citrate (1.25 mM), sodium alkyl sulfonate or sodium nitrate (0–30 mM), hydrogen peroxide (12.5 mM), and sodium borohydride (0.625 mM). Upon addition of the borohydride, nanocrystal nucleation and growth begin within seconds (Figures S3–S6 in Supporting Information).

We expected that the short-chain alkyl sulfonates (e.g., sodium 1-butanesulfonate) would not exhibit strong supramolecular interactions, while longer-chain alkyl sulfonates (e.g., sodium 1-octanesulfonate) would form supramolecular structures. A wide range of sodium alkyl sulfonate concentrations were tested, with an initial screening of sulfonate concentrations sweeping from 0.0001 mM to 30 mM in the nanocrystal reactions. Figure 1 highlights the resulting absorbance spectra of silver nanocrystals synthesized with four of the sulfonate concentrations (0 mM, 0.3 mM, 4.5 mM, 25 mM). As shown in Figure 1A, the same general spectra are obtained for all concentrations of butanesulfonate, with the wavelength of maximum absorbance (λ_max_) shifting slightly from approximately 750 to 800 nm as the butanesulfonate concentration increases to 25 mM. These reactions consistently produce nanocrystals that result in a blue solution color (Figure 1B). In contrast, as the concentration of octanesulfonate increases in Figure 1C, the absorbance of the nanocrystals shifts dramatically. When the concentration of octanesulfonate increases from 1 mM to 4.5 mM, the nanocrystals sweep through a range of absorbance maxima from approximately 700 to 450 nm. Interestingly, the 25 mM octanesulfonate sample shows a broad peak that falls between the peaks for the 0.3 mM and 4.5 mM samples and has a lower overall absorbance. Figure 1D showcases the variety of silver nanocrystal colors from reactions with octanesulfonate, with the sulfonate concentration increasing down the reaction well columns starting at the top left. The nanocrystals transform from vibrant blue to purple to orange to yellow. The striking color changes that result from the shifting absorbance peak of the silver nanocrystals with the octanesulfonate reveal that long-chain sulfonates have an influence over the silver nanocrystal properties, in contrast to the minimal changes observed for the short-chain alkyl sulfonate nanocrystals.

**Figure 1:**
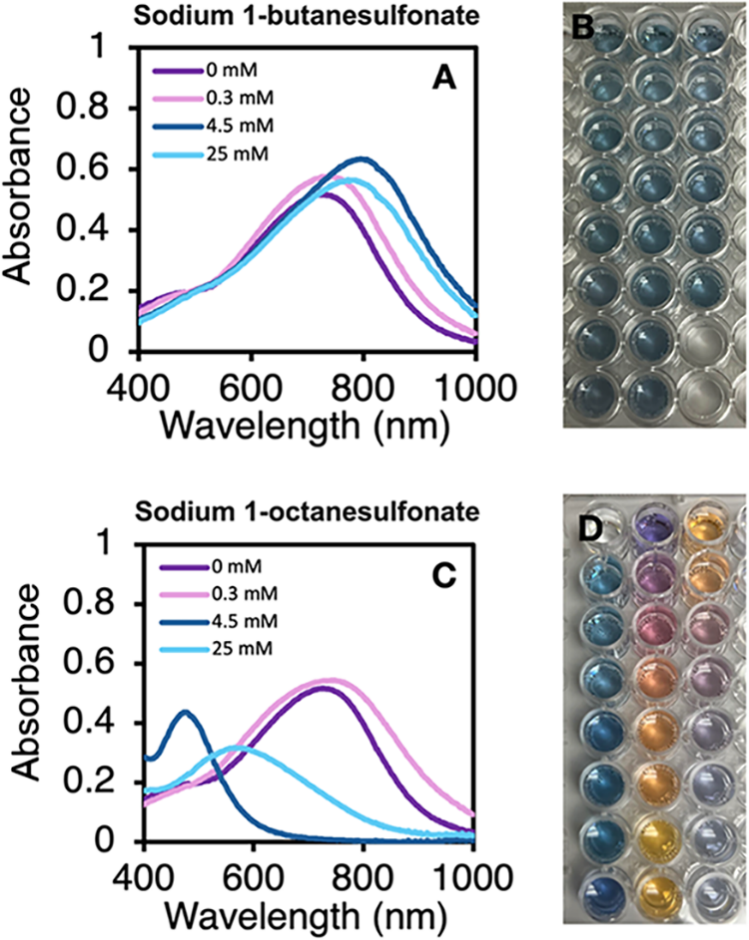
Absorbance spectra of silver nanocrystals with 0 mM, 0.3 mM, 4.5 mM, and 25 mM alkyl sulfonate synthesized with (A, top) sodium 1-butanesulfonate (C4) and (C, bottom) sodium 1-octanesulfonate (C8). Photos of corresponding nanocrystals, synthesized with alkyl sulfonate concentrations ranging from 0.1 mM to 30 mM starting with the lowest concentration in the upper left well and increasing down columns for (B) sodium 1-butanesulfonate (C4) and (D) sodium 1-octanesulfonate (C8). The last two wells in the bottom right in (B) and the first well in the upper left in (D) are water controls that do not contain nanocrystal reactions. Absorbance spectra (A, C) represent scans recorded 35 to 100 min after the addition of sodium borohydride to a reaction.

To further explore how the concentration and chain length of the alkyl sulfonates affects nanocrystal absorbance, we tested sulfonates with chain lengths of one (sodium 1-methanesulfonate, C1), four (sodium 1-butanesulfonate, C4), five (sodium 1-pentanesulfonate, C5), six (sodium 1-hexanesulfonate, C6), seven (sodium 1-hepantesulfonate, C7), and eight (sodium 1-octanesulfonate, C8) carbons between concentrations of 0.1 mM and 30 mM (see Tables S1–S7 in Supporting Information). We focused on this concentration range due to the limited stability of nanocrystals synthesized with higher concentrations of long-chain alkyl sulfonates (see Figures S7–S12 in Supporting Information). Figure 2 summarizes the results of over 1500 reactions by plotting the average λ_max_ against the concentration of the added alkyl sulfonate (on a logarithmic scale) for the sulfonates of focus. The average and standard deviation of the λ_max_ and absorbance intensity for each reaction type are provided in Tables S1–S7 in the Supporting Information. We were also interested in whether similar results would be obtained if the sulfonate functional groups were bound to a polymer, so we measured the full range of concentrations of polystyrenesulfonate (PSS) and polystyrenesulfonate co-maleic acid (PSSMA) polymers in the silver nanocrystal synthesis (Figure S13 in Supporting Information). These failed to yield similar effects on the absorbance spectra of the nanocrystals, in apparent contrast to a previous report on the use of PSS.^55^

**Figure 2:**
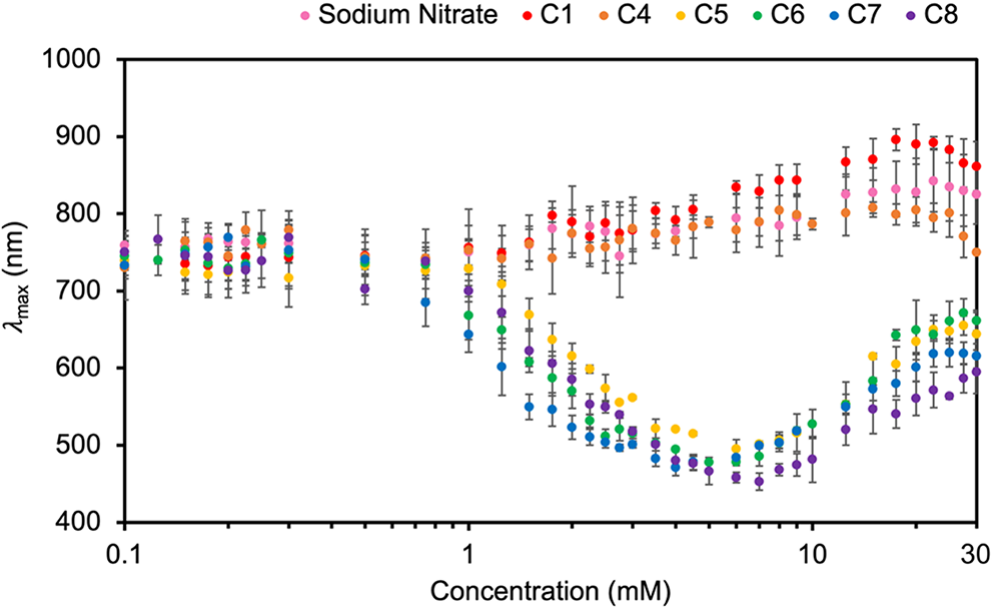
Wavelength of maximum absorbance (λ_max_) for silver nanocrystals synthesized with various concentrations of sodium alkyl sulfonates with different chain lengths (*C* = 1, 4, 5, 6, 7, 8) on a logarithmic scale. Reactions with sodium nitrate are included for comparison as an ionic strength control. Error bars represent one standard deviation (where not visible, the bars are within the size of the data points). Nanocrystals synthesized with high concentrations of sodium nitrate and sodium 1-methanesulfonate showed higher variability than other samples, the cause of which has not been determined.

Below 1 mM, all the sulfonates behave similarly in the synthesis, producing blue solutions of nanocrystals with λ_max_ between 700 and 800 nm. This result is comparable to the λ_max_ (∼735 nm) of nanocrystals without a sulfonate additive, suggesting that low concentrations of both short- and long-chain sulfonate have little influence over nanocrystal absorbance. Silver nanocrystals synthesized with sodium nitrate have λ_max_ consistently around 750 to 825 nm, despite the increasing ionic strength. Methanesulfonate (C1) and butanesulfonate (C4) exhibit similar wavelength trends to the sodium nitrate control up to concentrations of 10 mM.^56^ The butanesulfonate aligns better with the sodium nitrate control above 10 mM, with a slight blue-shift that becomes more evident at the highest concentrations; this could be the initial appearance of the supramolecular interactions that occur for the longer-chain sulfonates at lower concentrations.

Increasing the alkyl chain length of the sulfonate from four to five carbons has a profound impact on the silver nanocrystals produced from the synthesis. When less than 1 mM of pentanesulfonate (C5) is in the reaction, the resulting λ_max_ of the silver nanocrystals remain at ∼725 nm like the short-chain sulfonates. Above 1 mM of pentanesulfonate, however, the silver nanocrystals exhibit vibrant color changes. With 1 mM to 7 mM pentanesulfonate in solution, the silver nanocrystal color changes from blue to purple to pink to orange, which is demonstrated in the λ_max_ of the peak shifting from approximately 725 to 500 nm. At pentanesulfonate concentrations above 7 mM, the λ_max_ steadily returns to a peak at ∼650 nm, and the nanocrystal color transitions back to muted tones of purple and blue. This shift in λ_max_ wavelength between butanesulfonate and pentanesulfonate reveals a supramolecular threshold, where sulfonate carbon chains with at least five carbons modify the silver nanocrystal morphology and thus the absorption and solution color. At alkyl sulfonate concentrations above 7 mM, a similar red-shift is apparent for all long-chain sulfonates, with the longest-chain sulfonates resulting in the smallest red-shift. The blue-shift of λ_max_ wavelength upon increasing sulfonate concentration from 1 mM to 7 mM is inconsistent with an aggregative effect, which should cause a red-shift in the maximum absorbance. The red-shift at concentrations beyond 7 mM could result from aggregation.

Based on these results, it appears that the influence the alkyl sulfonates have on the silver nanocrystal morphology is related to a supramolecular interaction among the sulfonates, given the dependence on sulfonate chain length. Since ionic strength is known to affect critical micelle concentrations (CMCs) and some alkyl sulfonates have reported CMC values, a Nile Red fluorescence assay was used to estimate the CMC ranges of these alkyl sulfonates under the same ionic strength as the silver nanocrystal reaction conditions reported here. When micelles assemble in solution, the Nile Red sequesters inside the hydrophobic core of the micelles.^57^ A decrease in emission wavelength and increase in fluorescence intensity reveal a CMC range of 150 mM to 250 mM for octanesulfonate, the longest-chain sulfonate most prone to forming micelles in the reactions reported above. The experimental CMC range aligns with the reported literature values of 130 mM to 155 mM, measured using alternative techniques and under different ionic strength conditions (Figure S14 and Table S8 in Supporting Information).^58,59^ CMC values for the rest of the alkyl sulfonates were even higher, and all above the 30 mM maximum concentration used in our studies (Figure S14 and Table S8 in Supporting Information). This indicates that micelles are not forming under the reaction conditions, and thus micelle formation cannot explain the control of the silver nanocrystal absorption. This is reinforced by the observation that the preparation methods of the alkyl sulfonate solutions (e.g., mixing, annealing, etc.) did not have an impact on the absorbance of the silver nanocrystals; supramolecular structures are often kinetically trapped species that can be sensitive to handling conditions (Figures S15 and S16 in Supporting Information). We did, however, run some reactions with sodium 1-decanesulfonate (C10) for which we measured a CMC range of 30 mM to 50 mM (Figure S14 and Table S8 in Supporting Information) that aligns with the reported CMC value of 44 mM.^59^ The absorbance of the nanocrystals synthesized with high decanesulfonate concentrations *did* show sensitivity to the preparation conditions of the stock 100 mM decanesulfonate solution (Figures S15 and S16 in Supporting Information). This indicates that indeed some supramolecular structure that formed in the decanesulfonate stock solution likely persisted in the nanocrystal reaction. Further studies into this effect were limited by the stability of nanocrystals with high decanesulfonate concentrations (see Figures S10–S12 in Supporting Information), but we believe other systems that form micelles could produce similar results.

If micelles are not forming in the alkyl sulfonate solutions with eight or fewer carbons, yet there appears to be a supramolecular effect on the nanocrystals, we instead hypothesize that the nanocrystals are seeding the nucleation of sulfonate bilayers around the crystals. Bilayer structures formed around particles are known as admicelles and can assemble below the CMC thanks to the nucleation point of the solid.^60,61^ While we have no direct evidence of the bilayer structure as opposed to a more classic micelle structure (hemimicelle around a particle), we expect that under the aqueous conditions used in these reactions, the amphiphilic sulfonates would assemble into bilayers similar to the cetyltrimethylammonium bilayers reported to interact with gold nanorod surfaces.^31,32,34^ An important difference to the CTAB example is that most ligands thought to bind to gold or silver surfaces are negatively charged due to the partial positive charge of the metallic surface that comes about from the delocalization of the positive charge from a small fraction of oxidized metal atoms.^22,62^ Thus, the positively charged ammonium headgroup of CTAB would be electrostatically attracted to those ligands akin to a second coordination sphere of a coordination complex. In contrast, the negatively charged sulfonate headgroup would be repelled by negatively charged ligands, such as citrate in our case, which would be expected to outcompete the sulfonate for direct binding to the silver surface.^31^ We therefore believe that the formation of the supramolecular structure is driving the alkyl sulfonate interactions with the nanocrystals, and hypothesize that the electrostatic repulsion between the alkyl sulfonates and the citrate on the silver surface may be responsible for the observed changes in nanocrystal morphology and absorbance.

To directly observe the effects of the sulfonates on the morphology of the silver nanocrystals, a series of samples were prepared and analyzed using transmission electron microscopy (TEM). Silver nanocrystals were synthesized with butanesulfonate, hexanesulfonate, and octanesulfonate at low (0.5 mM), medium (4.5 mM), and high (25 mM) sulfonate concentrations and analyzed with TEM. Nanocrystals synthesized with sodium nitrate at equivalent concentrations were also analyzed as a control without alkyl sulfonates present (Figure S17 in Supporting Information). Unfortunately, the samples are highly polydisperse, making clear correlations between sulfonate conditions and nanocrystal structure challenging (Figures S17–S22 in Supporting Information). Whereas the published reaction that was adapted for this work resulted in relatively monodisperse samples of nanoplatelets with potassium bromide,^22^ the use of sulfonates results in far more polydisperse populations. We hypothesize that the sulfonates are disrupting the citrate surface ligands that are critical to the formation of the prismatic platelets, thereby causing the polydispersity. We expect that some of the observed effects are related to the thickness of the platelets (see Tables S9 and S10 in Supporting Information). However, the shape is also clearly playing a role as shown in Figures S17–S22 in the Supporting Information. Overall, the sulfonate chain length and concentration likely affect the nanocrystal population ensemble to provide the control over absorbance.

Efforts to study nanocrystal surface chemistry with nuclear magnetic resonance (NMR), infrared, and Raman spectroscopies, high performance liquid chromatography–mass spectrometry, and capillary electrophoresis were inconclusive and challenging given the limited concentrations of the particles and the large particle sizes compared with other nanocrystal systems such as quantum dots. Additionally, we expect that the alkyl sulfonate molecules in the admicelle are in dynamic equilibrium with free alkyl sulfonate in solution and that this equilibrium would further complicate surface chemistry studies. While solution-phase NMR seems like a strong candidate to study organic molecules interacting with the surface of silver nanocrystals and recent work from the Murphy group has indeed shown the utility of this technique for studying midsized gold nanocrystals,^63^ we were unable to obtain useful data from the technique. Based on the size, polydispersity, and dynamic surface of the alkyl sulfonate silver nanocrystals, it is unsurprising that they are outside the capabilities of this method (see NMR Studies Discussion in Supporting Information).^40,41^ Furthermore, the small size of the alkyl sulfonates relative to the overall size and polydispersity of the nanocrystals means that the admicelle sulfonate bilayer would be within the error of hydrodynamic radius measurements, limiting the usefulness of dynamic light scattering for these systems.

Limited by more direct observations, we attempted to probe our hypothesis through additional reactions. We investigated the effects of combining different sulfonates in the same reaction. In these combination studies, a total sulfonate concentration of 20 mM was introduced into the silver nanocrystal reactions. The composition of the total sulfonate concentration was varied with ratios of two different chain length sulfonates. When two long-chain sulfonates, hexanesulfonate and octanesulfonate, were used together in the reaction solution, the resulting λ_max_ values of the nanocrystal absorbance spectra fall between the values of nanocrystals synthesized with only hexanesulfonate or octanesulfonate, as shown in Figures 3A and S23A. We expect that an average admicelle forms between what the hexanesulfonate or octanesulfonate would independently form.

**Figure 3:**
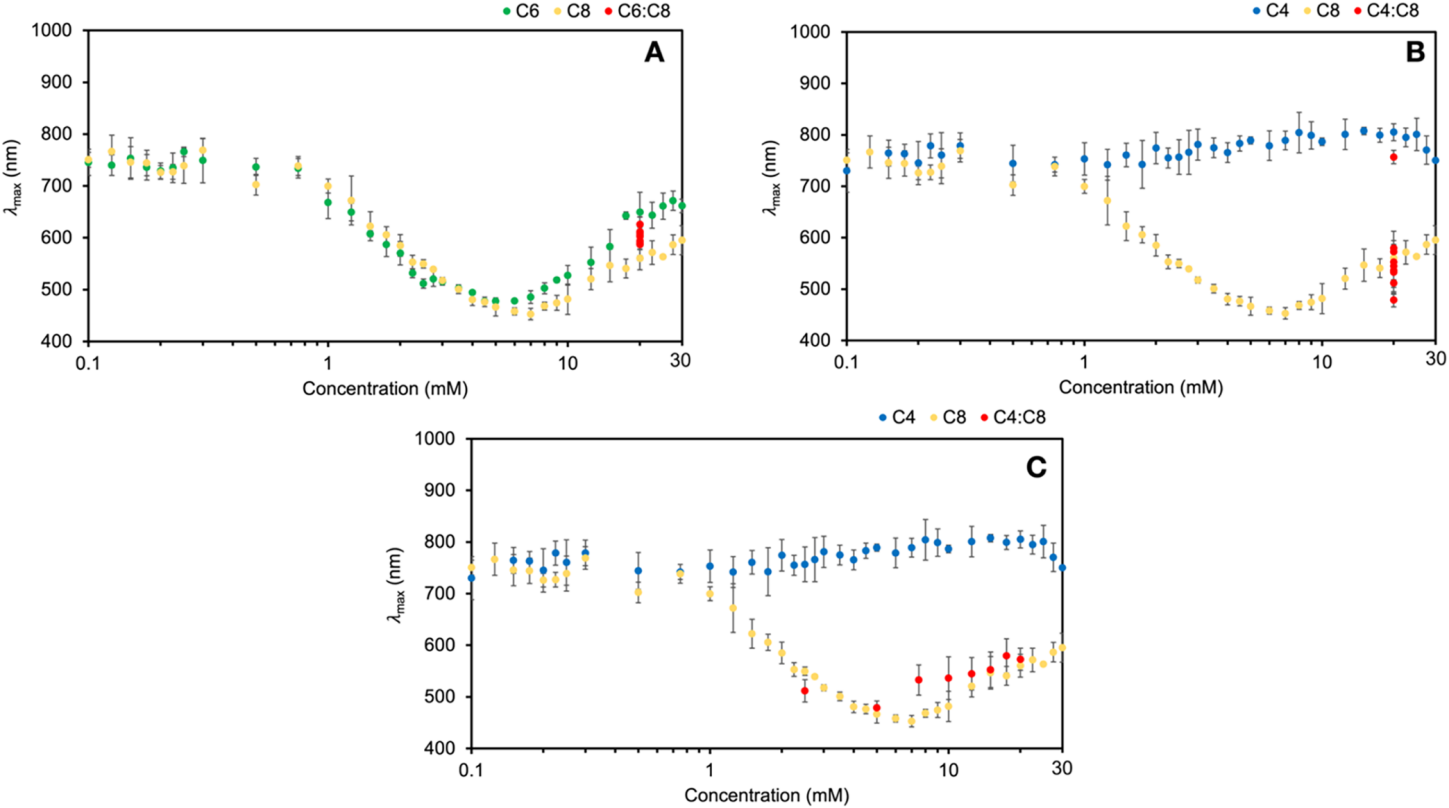
λ_max_ of silver nanocrystals with a constant total 20 mM sulfonate concentration and varying ratios of sulfonates with different chain lengths on a logarithmic scale. (A) λ_max_ of nanocrystals synthesized with varying ratios of sodium 1-octanesulfonate (C8) and sodium 1-hexanesulfonate (C6) plotted with respect to the total sulfonate concentration in solution, 20 mM. (B) λ_max_ of nanocrystals with varying ratios of sodium 1-octanesulfonate (C8) and sodium 1-butanesulfonate (C4) plotted with respect to the total sulfonate concentration in solution, 20 mM. (C) λ_max_ between of nanocrystals with varying ratios of sodium 1-octanesulfonate (C8) and sodium 1-butanesulfonate (C4) plotted with respect to the concentration of C8 in the reaction. Wavelength ranges were restricted to capture the peaks of interest (see Figure S24 in Supporting Information). Error bars represent one standard deviation (where not visible, the bars are within the size of the data points).

When a long-chain sulfonate (octanesulfonate) and a short-chain sulfonate (butanesulfonate) were simultaneously introduced into the reaction solution, the resulting UV–vis spectra and λ_max_ values do not fall within the absorbance profiles of the two sulfonates independently (Figure 3B) and instead are correlated more closely to that expected of reactions with only octanesulfonate (Figures 3C and S23B). Based on the earlier results, we believe that butanesulfonate does not have enough nonpolar character to form admicelles under these conditions, and therefore it does not contribute to the supramolecular structure in a significant way.

We further investigated the influence of long-chain sulfonates on nanocrystal absorbance with the addition of sulfonates to already-synthesized silver nanocrystals. Nanocrystals were synthesized with 10 mM butanesulfonate and allowed to grow for 20 min before additional sulfonate (or sodium nitrate) was added to a reaction well. When 10 mM sodium nitrate, methanesulfonate, or butanesulfonate was introduced postsynthesis, the absorbance spectra of the silver nanocrystals were similar to that of the control reaction without an additive (Figure 4A). However, upon addition of 10 mM of a longer-chain sulfonate, pentanesulfonate or hexanesulfonate, to the butanesulfonate nanocrystals, the resulting absorbance spectra display two peaks, one near 700 nm and the other near 400 nm (Figure 4A). This change in absorbance could correspond to a transition period in particle shape and size, where the addition of a longer chain sulfonate causes a change in the distribution of particle morphologies in the sample. Finally, when 10 mM of the sulfonates with the longest chain lengths (heptanesulfonate, octanesulfonate, and decanesulfonate) are added to butanesulfonate nanocrystals, the absorbance spectra display a narrower peak at approximately 400 nm (Figure 4A). Kinetics studies and the corresponding video of the octanesulfonate postsynthesis addition in scaled-up reactions illustrate that the absorbance change happens in seconds, with the color of the reaction solution turning from dark blue to yellow in less than 10 s (Figure S25 and attached Video in Supporting Information). However, when butanesulfonate or sodium nitrate is used as the postsynthesis additive, no color change is observed, with the nanocrystal solution remaining blue in color (Figure S25 in Supporting Information). These observations support the proposal that the alkyl sulfonates are not impacting the reaction pathway of the nanocrystals because significant changes to the absorbance are observed whether the long-chain sulfonates are originally in the solution during the formation of the nanocrystals or added subsequent to their formation. Instead, this aligns with the hypothesis that that long-chain alkyl sulfonates are influencing the morphology of the crystals through surface interactions either directly with the surface or indirectly by influencing the binding of the citrate ligands, and that these nanocrystals are quite dynamic, such that this can occur in seconds.

**Figure 4:**
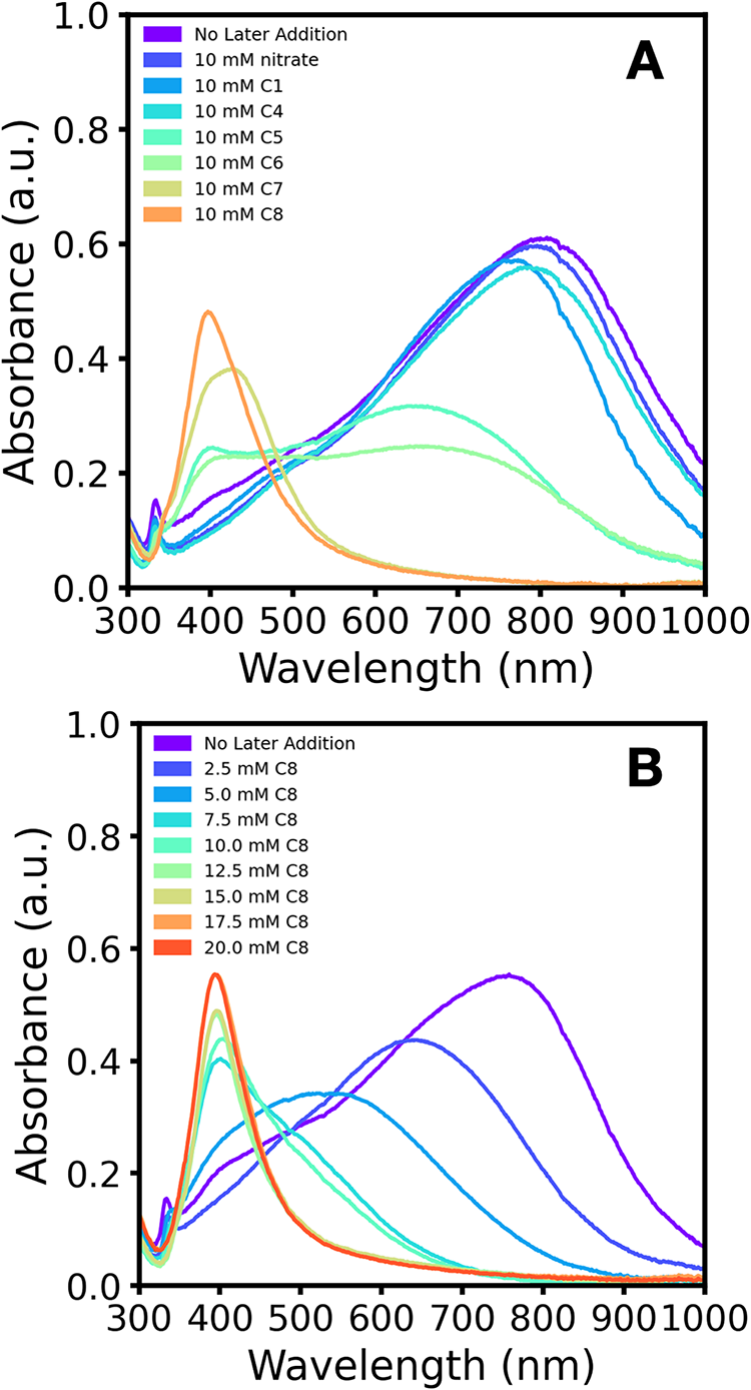
Absorbance spectra of 10 mM sodium 1-butanesulfonate (C4) silver nanocrystals to which additional sulfonate was added postsynthesis. (A) Absorbance spectra of silver nanocrystal reactions to which 10 mM of sodium nitrate, sodium 1-methanesulfonate (C1), sodium 1-butanesulfonate (C4), sodium 1-pentanesulfonate (C5), sodium 1-hexanesulfonate (C6), sodium 1-heptanesulfonate (C7), or sodium 1-octanesulfonate (C8) was added approximately 20 min after initial nanocrystal synthesis. Spectra were recorded between 28 and 34 min after the addition of sulfonate to the reaction wells. A control reaction (scanned 61 min after borohydride addition) with no postsynthesis additive is included for comparison. (B) Absorbance spectra of silver nanocrystal reactions to which 2.5, 5.0, 7.5, 10.0, 12.5, 15.0, 17.5, or 20 mM of sodium 1-octanesulfonate was added approximately 20 min after initial nanocrystal synthesis. Spectra were recorded between 30 and 34 min after the addition of sodium 1-octanesulfonate to the reaction wells. A control reaction (scanned 68 min after borohydride addition) with no postsynthesis addition of sodium 1-octanesulfonate is included for comparison.

Additional postsynthesis studies starting with 10 mM butanesulfonate nanocrystals revealed that relatively low concentrations of a long-chain sulfonate are required to initiate the blue-shift in absorbance. Concentrations of 2.5 mM and 5 mM octanesulfonate progressively shift the λ_max_ values of the nanocrystals to about 650 and 550 nm, respectively (Figure 4B). At concentrations at or above 7.5 mM octanesulfonate, the nanocrystals to which sulfonate has been added have a λ_max_ at about 400 nm, displaying a narrow band that grows in as octanesulfonate concentration increases to 17.5 mM (Figure 4B). This blue-shift occurs regardless of the time that the octanesulfonate was added to the nanocrystals, suggesting that this effect is not dependent on the ongoing decomposition of excess reagents or the formation of coproducts (Figure S26 in Supporting Information). When sodium nitrate was added to nanocrystal samples postsynthesis at equivalent concentrations, a notable blue-shift in the lambda maxima of the resulting nanocrystals was not observed (Figure S27 in Supporting Information). However, a similar blue-shift did occur when sulfonate was added postsynthesis to 10 mM butanesulfonate particles that had been purified from the reaction solution (Figure S28 in Supporting Information), further indicating that the effect does not require the presence of coproducts or leftover precursors. Comparable trends were also observed for particles with a different shape and different ligands; both bromide-capped silver nanoprisms and hexadecyltrimethylammonium chloride-capped silver nanocubes showed little change upon addition of sodium nitrate or butanesulfonate, but distinct absorbance changes upon addition of octanesulfonate (see Figures S29–S34 in Supporting Information). The nanocrystal absorbance changes resulting from the addition of the long-chain sulfonates, like octanesulfonate, could be due to the formation of sulfonate bilayers around the already-grown nanocrystals. The bilayers might destabilize the citrate bound to the silver surface, allowing for oxidative etching of the exposed particle surface. This could cause morphological changes or even dissolution and regrowth of the particles, which is supported by TEM images that show particles that appear more round in shape and of possibly different thickness (Figure S21 in the Supporting Information). These changes correspond with the shift in absorbance spectra that we observe (Figure S21 in the Supporting Information).^64^

## Conclusions

This work demonstrates that long-chain alkyl sulfonates have a pronounced effect on the absorption of silver nanocrystals, while short-chain sulfonates have minimal influence on their absorption. The fact that alkyl sulfonates show any effect on silver nanocrystal synthesis is somewhat surprising given that the sulfonate group should not strongly interact with silver. Nonetheless, long-chain sulfonates can be used to tune the λ_max_ of silver nanocrystals throughout the visible spectrum, based on the length of the alkyl chain and concentration of sulfonate. We attribute this observation to the formation of a supramolecular structure by the long-chain sulfonates that occurs below the CMC of these sulfonates under the reaction conditions. We therefore propose that the long-chain alkyl sulfonates are nucleating bilayers around the silver nanocrystals to form admicelles, which are known to occur below the CMC. Perhaps most striking is that the addition of long-chain sulfonates to previously synthesized silver nanocrystals result in their absorbance spectra shifting to resemble those synthesized with the long-chain sulfonate in seconds. This result suggests that the alkyl sulfonates are not influencing the reaction pathway of the nanocrystals but are instead impacting the absorption of the nanocrystals by adjusting their morphology through surface interactions, which seem quite dynamic. This work furthers our understanding of the role that anionic surfactants can play in silver nanocrystal synthesis.

Determining the precise way in which the sulfonates are changing the nanocrystal morphology to influence their absorbance spectra is complicated by the polydispersity of the nanocrystal samples. Additional efforts to directly measure the alkyl sulfonates interacting with the surface of the nanocrystals were inconclusive. This means that the evidence for our hypotheses related to the formation of admicelles and possible influence on citrate binding is circumstantial, and further work is needed to provide more direct evidence of these proposals. Indeed, we believe that developing analytical techniques to study the surface chemistry of these low-concentration, large nanostructures is critical to advance our understanding of these materials. Finally, while the nanocrystal samples formed herein may be too polydisperse for many purposes, the combination of biologically compatible sulfonate groups and the antimicrobial properties of silver nanoparticles continue to make these types of materials scientifically and commercially relevant.

## Methods

### Chemicals

Silver nitrate (AgNO_3_, 99%), hydrogen peroxide (H_2_O_2_, 3% w/w), trisodium citrate (Na_3_C6H5O7, 99% min), l-ascorbic acid (99.0%), and methanol (CH_3_OH, 99.8% min) were purchased from VWR Chemicals. Potassium bromide (KBr, 99% min), iron­(III) chloride, anhydrous (98.0%), silver trifluoroacetate (min. 98%) and sodium borohydride (NaBH4) were purchased from STREM Chemicals, Inc. Sodium methanesulfonate (98% min) was purchased from TCI Chemicals. Sodium 1-hexanesulfonate (99%), and sodium 1-decanesulfonate (99% dry weight) were purchased from Beantown Chemicals. Sodium 1-butanesulfonate was purchased from Beantown Chemicals (99%) and Thermo Scientific (99%). 1-octanesulfonic acid sodium salt (98% min), sodium nitrate (≥99.5%), poly­(sodium 4-styrenesulfonate) (PSS, average MW ∼ 70,000, powder), poly­(4-styrenesulfonic acid-*co*-maleic acid) sodium salt (PSSMA 1:1, 1:1 4-styrenesulfonic acid:maleic acid mol ratio, average MW ∼ 20,000, powder), poly­(4-styrenesulfonic acid-*co*-maleic acid) sodium salt (PSSMA 3:1, 3:1 4-styrenesulfonic acid:maleic acid mol ratio, MW ∼ 20,000, powder), and sodium chloride (NaCl, 99.0% min), were purchased from Sigma-Aldrich. Nile Red (99%) was purchased from Thermo Scientific. (1-hexadecyl)­trimethylammonium chloride (96%) was purchased from Alfa Aesar. Chemicals were used without additional purification. All solutions were prepared with Milli-Q water from a Milli-Q water purification system with a resistivity of 18.2 MΩ·cm unless otherwise specified. ^1^H NMR was used to confirm the absence of organic impurities in the alkyl sulfonates, sodium borohydride, and trisodium citrate. Sodium borohydride was made fresh daily, and hydrogen peroxide was used fresh from the manufacturer’s bottle each day, but the age of the bottle varied across experiments. Other solutions were stored as stock solutions for weeks to months at a time.

### Sulfonate Solution Preparation

Most sodium sulfonate stock solutions (100 mM or 250 mM) were equilibrated to ensure that the solid fully dissolved. Milli-Q water was added to centrifuge tubes with the solid sodium sulfonate. The solutions were placed in a hot water bath between 90 and 100 °C for an hour and then slow-cooled. The tubes were inverted twice (after being in the hot water bath for 30 and 60 min) to encourage dissolution. The 100 mM sodium sulfonate stock solutions were used to prepare 10 mM and 1 mM solutions by serial dilution for use in nanocrystal synthesis. Some sulfonate solutions were prepared using a different method that consisted of a placement in a warm water bath (40–45 °C) for 10 min followed by vortexing for 15 min. While the equilibration method of the decanesulfonate stock solution equilibration was shown to influence nanocrystal absorbance, the equilibration method did not affect nanocrystal absorbance for the next longest-chain sulfonate, octanesulfonate (see Figures S9 and S10 in Supporting Information). Therefore, stock solutions of alkyl sulfonates with 8 or fewer carbons were prepared via both equilibration methods and used in nanocrystal synthesis.

### Automated Synthesis of Silver Nanocrystals

All automated experiments were conducted with an Opentrons OT-2 liquid-handling robot integrated with a UV–vis plate reader (BMG BioTech SpectroSTAR Nano) to allow for direct pipetting of reagents into a well plate and spectroscopic characterization of samples without the need for human intervention (see Figure S2 in the Supporting Information).^54^ Nanocrystals were synthesized following a silver nanoprism synthesis modified from original work by Métraux and Mirkin that was later developed by Frank et al.^22,48^ Reactions were conducted at 200 μL volumes in a 96-well microplate (VWR, poly­(ethylene terephthalate)). The sodium borohydride and hydrogen peroxide solutions were prepared fresh each day by the robot for use in the experiment. For experiments taking more than 3 h to complete, a second solution of sodium borohydride was prepared for use midway through the experiment. To prepare sodium borohydride, Milli-Q water was first cooled on a temperature controller set at 4 °C for the duration of the experiment. The cold Milli-Q water was added by the robot to a tube on the temperature controller containing solid sodium borohydride to prepare a 130 mM solution. The 130 mM sodium borohydride was then diluted by the robot to 6.25 mM for use in the nanocrystal reactions. 3% w/w (∼890 mM) hydrogen peroxide was diluted to 50 mM hydrogen peroxide by the robot at room temperature for use in the nanocrystal reactions. The hydrogen peroxide was prepared in a dark tube to prevent decomposition during the experiment. After preparing a solution or performing a dilution, the robot arm automatically mixes by pipetting with a 300 μL tip at four heights spread equally in a range from 8 to 58 mm below the liquid height, such that the pipet tip is never fully submerged in the liquid. At each height, 300 μL of liquid is aspirated and dispensed four times at a rate of 100 μL s^–1^, resulting in 16 total cycles per mix.

In a standard automated silver nanocrystal experiment, Milli-Q water was first added to each reaction well to maintain a final volume of 200 μL in each well. In the following order, 20 μL of 12.5 mM trisodium citrate, 50 μL of 0.375 mM silver nitrate, 0–60 μL of 1, 10, 100, or 250 mM of sodium sulfonate or sodium nitrate, and 50 μL of 50 mM hydrogen peroxide were pipetted by the robot into the reaction wells. Directly after the robotic pipet system obtained a reagent to transfer across the deck, the pipet tip was touched against each cardinal edge of the tube to remove any excess solution. Each of the reagents were dispensed into all the reaction wells before the following reagent was added. To mix reagents, the 96-well plate was shaken (double orbital mix at 300 rpm) in the spectrometer at various time points throughout the experiment. After the addition of hydrogen peroxide to the final reaction, the well plate was shaken for 30 s in some of the experiments. Upon addition of 20 μL of 6.25 mM sodium borohydride to a reaction, the 96-well plate was shaken in the UV–vis plate reader for 30 s and the reaction well was scanned to collect an initial absorbance spectrum. After sodium borohydride was added to the final reaction in the experiment, the well plate was shaken for an additional 1 min. Importantly, the silver nanocrystal reactions are sensitive to the mixing conditions, as shown in Figure S1 in the Supporting Information. UV–vis spectra were then collected for all the reactions in the experiment at 5 min increments over a 20 min time frame. Reactions in each experiment were run in duplicate.

### Data Analysis

Every automated experiment included an initial well with a Milli-Q water blank that was scanned and subtracted from the reaction spectra prior to data analysis. In Figure 2 in the main text, the reported absorbance data represents the scan recorded closest to 45 min after the addition of sodium borohydride to a reaction. These scans ranged from 30 to 80 min following borohydride addition. Some reaction wells contained a bubble that interfered with UV–vis absorbance measurements, and this data was removed prior to statistical analysis. Dixon’s *r*_10_ two-tailed Q test was then used to reject outliers for reactions with more than three replicates at the 99% confidence interval.^65^ Each reaction type in Figure 2 represents an average of data from three or more replicate reactions.

### Instrumentation

UV–vis spectra were recorded from 300 to 1000 nm on a SPECTROstar Nano plate reader from BMG Labtech that was integrated into the Opentrons OT-2 liquid-handling robot, as shown in Figure S2 in the Supporting Information. All samples were centrifuged using a Centrifuge 5420 from Eppendorf. Transmission electron microscopy imaging was done on an FEI Tecnai G2 F20 SuperTwin microscope operated at 200 kV using bright field imaging. Information about TEM sample preparation and analysis is provided on page 4 of the Supporting Information. Fluorescence-emission spectra were recorded with an Agilent Cary Fluorescence Spectrometer. The excitation wavelength was set at 550 nm and emission spectra were recorded between 570 and 700 nm at a scan rate of 13 nm/min. Information about fluorescence sample preparation is provided on page 4 of the Supporting Information.

## Supplementary Material





## Notes

Additional experimental methods and data, including studies related to stirring, kinetics, nanocrystal stability, sulfonate polymers, Nile Red fluorescence, sulfonate equilibration, sulfonate combinations, postsynthesis addition of sulfonates, transmission electron micrograph data, and a brief discussion of NMR experiments are provided (PDF)
Post-synthesis C8 addition (MOV)
