# Supplementary material for: Supramolecular Effects of Alkyl Sulfonates in Silver Nanocrystal Synthesis
Source: ACS Nanosci Au. 2025 Dec 4;6(1):129–38. doi: 10.1021/acsnanoscienceau.5c00121 (PMC12921613; doi:10.1021/acsnanoscienceau.5c00121)
Supplement: Supplementary file 1 [file ng5c00121_si_001.pdf]

Supporting Information for

Supramolecular Effects of Alkyl Sulfonates in Silver Nanocrystal Synthesis

Nicola L. Myers[1#], Clara M. Hansen[1#], Clare N. Hermanson[1#], Keenan Tiddle[1], Grant Didway[1], Noah Kaplan[1], Helen C. Larson[2], Catherine C. Bodinger[2], Brandi M. Cossairt[2], Steven M. Hughes[3], Mark P. Hendricks[1]*

[1]*Department of Chemistry, Whitman College, Walla Walla, WA 99362*

[2]*Department of Chemistry, University of Washington, Seattle, WA 98195*

[3]*Department of Chemistry, Roanoke College, Salem, VA 24153*

*Corresponding author: hendrimp@whitman.edu

[#]equal contribution

Supplemental Materials and Methods **4**
Manual Silver Nanocrystal Synthesis with Alkyl Sulfonates 4
Silver Nanocube Synthesis **4**
Kinetics Studies 5
Micelle Studies with Fluorescence 5
Equilibration of Sodium 1-decanesulfonate & Sodium 1-octanesulfonate 6
Transmission Electron Microscopy 6
Sulfonate Combination Studies **7**
Post-Synthesis Ligand Addition Studies 7
Kinetics of Post-Synthesis Ligand Addition 8
Supplemental Results & Figures **10**
Stirring Study 10
Opentrons OT-2 Robot Experimental Set-Up 12
Kinetics of Alkyl Sulfonate Nanocrystal Synthesis 13
Alkyl Sulfonate Data 17
Effect of High Sulfonate Concentration on Nanocrystal Absorbance 24
Effect of High Sulfonate Concentration on Nanocrystal Growth Kinetics 25
Silver Nanocrystal Long-Term Kinetics Studies 27
Polystyrene Sulfonate Nanocrystal Synthesis 30
Micelle Studies Using Fluorescence Spectroscopy 31
Equilibration Method Study 33
Transmission Electron Microscopy 36
NMR Studies Discussion 44
Sulfonate Combination Studies: % Sulfonate vs λ_{max} 45
Sulfonate Combination Studies: Absorbance Spectra 46
Kinetics of Post-Synthesis Ligand Addition 47
Time Variation of Post-Synthesis Sulfonate Addition 48
Post-Synthesis Addition of Sodium Nitrate 49
Post-Synthesis Sulfonate Addition to Purified Sulfonate Nanocrystals 50
TEM for Post-Synthesis Sulfonate Addition to Bromide Nanocrystals 51
Post-Synthesis Sulfonate Addition to Nanocubes 55
Post-Synthesis Sulfonate Addition to Purified Nanocubes 56
TEM of Purified Nanocubes with Post-Synthesis Sulfonate Addition 57

References **58**

Supplemental Materials and Methods

Manual Silver Nanocrystal Synthesis with Alkyl Sulfonates

Nanocrystals were synthesized following the procedure described for the automated synthesis that was inspired from initial work by Métraux and Mirkin and later developed by Frank et. al.[1,2] For the manual syntheses, reactions were conducted at 1 mL total volumes in plastic 24-well plates (VWR, polyethylene terephthalate, Tissue Culture Plates, non-treated, sterilized). A solution of 130 mM sodium borohydride was prepared in ice-cold Milli-Q water. The 130 mM solution was diluted in ice-cold Milli-Q water to 6.25 mM for use in the nanocrystal reactions. The 6.25 mM solution was stored on ice for the duration of the manual syntheses. Hydrogen peroxide was diluted from a 3% w/w (~890 mM) stock solution to 50 mM in an opaque 15 mL plastic centrifuge tube for use in the reactions. Autopipettes were used to add reagents to reaction wells. Reagent addition followed the same order as the standard automated experiments. Each of the reagents was dispensed into all the reaction wells before addition of the next reagent began. Milli-Q water was added first in the volume required to maintain a total reaction volume of 1 mL. Next, 100 µL of 12.5 mM trisodium citrate and then 250 µL of 0.375 mM silver nitrate were added. The volume and concentration of sulfonate was determined by the constraint of the total sample volume (1 mL) and the desired reaction concentration of sulfonate such that additions used 0 – 250 µL of 1 mM, 10 mM or 100 mM sulfonate). Finally, 250 µL of 50 mM hydrogen peroxide and 100 µL of 6.25 mM sodium borohydride were added to each well. Manual nanocrystal syntheses were conducted with two different mixing conditions. The first mixing condition consisted of using the plate reader to shake the 24-well plate using the same settings as for automated syntheses (double orbital mix at 300 rpm). The plate was shaken for 30 seconds after hydrogen peroxide addition was complete and after the addition of borohydride to each well. A 60 second shake was executed at the conclusion of the experiment and the reactions in the well plate were scanned approximately 45 minutes later. The second mixing condition consisted of continuous stirring at 350 rpm using a magnetic stir bar in each reaction well. Stirring began upon the addition of Milli-Q water and continued until the well plate was scanned (approximately 45 minutes after sodium borohydride addition) with the UV-Vis spectrometer (from 300 – 1000 nm) on the plate reader.

Silver Nanocube Synthesis

Silver nanocubes were synthesized following the procedure from Zhou et. al.[3] Solutions of 20 mM hexadecyltrimethylammonium chloride (CTAC), 100 mM ascorbic acid, 10 mM silver trifluoroacetate, and 4.29 µM iron(III) chloride were prepared in Milli-Q water in glass vials with a stir bar used when needed. 5 mL of 20 mM CTAC and 0.5 mL of 100 mM ascorbic acid were added to a glass vial heated to 60 °C with a magnetic stir bar and allowed to stir for 10 minutes. 80 µL of 4.29 µM iron (III) chloride and 50 µL of silver trifluoroacetate were then simultaneously injected into the reaction solution from separate pipettes. The reaction vial was capped and stirred at 60 °C for approximately six hours. The resulting nanocubes were allowed to cool prior to use in post-synthesis ligand addition experiments. For nanocubes that were purified, 1.6 mL of reaction solution was added to each of two 2 mL centrifuge tubes. The tubes were centrifuged for 10 minutes at 9391 rcf, after which a pellet formed in the tube. The pellet was removed by scraping the solid while aspirating 50 µL into a pipette tip, which was repeated for a total of two aspirations to remove 100 µL per tube. To approximately double the nanocube concentration, 700 µL of Milli-

Q water was added to the 100 μL containing the pellet that was removed from each tube, and the tube was then vortexed. This procedure was repeated at a 3× scale for screening the nanocubes in the automated experiments without noticeable difference, and in cases where multiple centrifuge tubes were used in a single experiment, the final nanocube samples were combined before use to ensure all samples were the same concentration.

Kinetics Studies

Kinetics experiments were conducted with the interfaced Opentrons OT-2 liquid-handling robot and UV-Vis plate reader system. Silver nanocrystals were synthesized with low (0.5 mM), medium (5 mM), and high (25 mM) concentrations of sodium 1-butanesulfonate, sodium 1-hexanesulfonate, sodium 1-octanesulfonate, and sodium 1-decanesulfonate following the standard automated synthesis procedure. Milli-Q water, trisodium citrate, silver nitrate, sodium alkyl sulfonate, and hydrogen peroxide were added to a set of three reaction wells, consisting of a reaction each for the low, medium, and high concentrations of alkyl sulfonate. Each reagent in the synthesis was dispensed into the set of three wells before addition of the next reagent. After the addition of sodium borohydride to the first reaction well, the plate was shaken for 30 seconds, and the reaction well was scanned with the UV-Vis spectrometer every 30 seconds for a total of 20 scans. Sodium borohydride was then added to the next reaction well, the plate was shaken, and the reaction well was scanned in the same format. This procedure was then repeated for nanocrystal reactions with low, medium, and high concentrations for each of the four alkyl sulfonates. All reported kinetics data is representative of one reaction for each reaction type.

Long-term kinetics were similarly conducted with the interfaced Opentrons OT-2 liquid-handling robot and UV-Vis plate reader system. Silver nanocrystals were synthesized with medium (5 mM) and high (25 mM) concentrations of sodium 1-butanesulfonate, sodium 1-octanesulfonate, and sodium 1-decanesulfonate following the standard automated synthesis procedure. Each reaction type was conducted in duplicate, and all reactions were carried out at the beginning of the experiment, with an initial absorbance spectrum recorded after sodium borohydride was introduced to a well as in a typical automated experiment. After the addition of sodium borohydride to the final reaction well, all wells were scanned every minute for 5 minutes, followed by scans every 5 minutes for 40 minutes, then every 10 minutes for 90 minutes, and then every 20 minutes for 160 minutes. The final scan time varied (338 minutes to 358 minutes) from the time of borohydride addition to a well. Long-term kinetics studies were conducted with the same protocol for reactions with sodium 1-butanesulfonate and sodium 1-octanesulfonate concentrations above 30 mM (31.3 mM, 43.8 mM, 56.3 mM, 75.0 mM).

Micelle Studies with Fluorescence

Fluorescence-emission spectra were recorded with an Agilent Cary Fluorescence Spectrometer. The excitation wavelength was set at 550 nm and emission spectra were recorded between 570 nm and 700 nm at a scan rate of 13 nm/min. Samples were prepared following a procedure modified from da Silva et. al.[4] A 2 mM Nile Red stock solution was prepared in methanol and diluted daily to 0.05 mM in Milli-Q water. Samples were prepared with 25 μL of 0.05 mM Nile Red, 2.45 mL of sodium alkyl sulfonate and 25 μL of 821 mM sodium chloride to match the ionic strength of the nanocrystal reactions synthesized following the automated procedure. Sulfonate concentrations ranged from 1 mM to 50 mM for sulfonates with 6 or fewer carbons and from 30 mM to 400 mM

for sulfonates with 7 or more carbons. Sulfonate stock solutions were equilibrated following the protocol using the hot water bath as described in the main text and then diluted to lower concentrations. The samples were wrapped with aluminum foil to minimize light exposure and were allowed to equilibrate for 24 to 48 hours before emission spectra were recorded.

Equilibration of Sodium 1-decanesulfonate & Sodium 1-octanesulfonate

UV-Vis spectra were collected for silver nanocrystals synthesized with sodium 1-decanesulfonate concentrations between 0.1 and 25 mM. Two methods were used to prepare the 100 mM sodium 1-decanesulfonate and 100 mM sodium 1-octanesulfonate stock solutions. In the first equilibration method, the stock solutions were equilibrated through heating and slow cooling. Solutions were placed in a hot water bath (90-95 °C) for an hour, inverted after 30 and 60 minutes to encourage dissolution, and then allowed to slow-cool (Figure S10, green and orange). In the non-equilibration method, 100 mM sodium 1-octanesulfonate and 100 mM sodium 1-decanesulfonate solutions were placed in a warm water bath (~45 °C) for 10 minutes and then immediately vortexed for 15 minutes (Figure S10, purple and blue). 100 mM stock solutions were serially diluted to the 1 mM and 10 mM solutions that were also used for silver nanocrystal synthesis.

Transmission Electron Microscopy

Imaging was done on an FEI Tecnai G2 F20 SuperTwin microscope operated at 200 kV using bright field imaging. Silver nanocrystal samples with concentrations of 0.5 mM, 4.5 mM or 25 mM sodium nitrate, sodium 1-butanesulfonate, sodium 1-hexanesulfonate, or sodium 1-octanesulfonate were synthesized at 200 µL volumes following the standard automated synthesis procedure. Reactions were conducted in sets of three for each additive type. Absorbance spectra for the three reactions were collected with plate reader scans of the 96-well plate between 41 and 47 minutes after the addition of sodium borohydride to the final reaction. Three 10 mM sodium 1-butanesulfonate nanocrystal reactions were synthesized for post-synthesis studies. Reaction concentrations of 2.5 mM, 6.25 mM, and 10 mM sodium 1-octanesulfonate were added to the 10 mM sodium 1-butanesulfonate nanocrystals that had been allowed to grow for about 20 minutes. The reactions were scanned to collect absorbance spectra no less than 15 minutes later. Silver nanocrystals were also synthesized with 0 mM, 0.5 µM and 1.5 µM potassium bromide concentrations (following the automated procedure described in the Post-Synthesis Ligand Addition Studies Section). 10 mM sodium 1-butanesulfonate or sodium 1-octanesulfonate was then introduced to the reactions 20 minutes post-synthesis and absorbance spectra were recorded approximately 20 minutes later. Control samples in which there was no later sulfonate addition were also prepared and spectra recorded 40 to 45 minutes after the addition of sodium borohydride to a reaction (see Post-Synthesis Ligand Addition Studies Section for synthesis information). Following the collection of absorption data for a reaction set, 10 µL of each nanocrystal reaction solution was pipetted onto a copper TEM grid (carbon film on 300 mesh CU grids from Electron Microscopy Sciences) and allowed to dry (about 1 hour). TEM analysis was performed manually with ImageJ based on images from at least two different grid locations and 16 to 54 particle measurements per sample. The thickness of nanocrystals suspected to be nanoplatelets standing on their side was measured by fitting a rectangular box along the perimeter to ensure measurements were taken at an angle perpendicular to the long edge.

Sulfonate Combination Studies

Nanocrystals were synthesized following the standard automated synthesis described in the main text. Following the silver nitrate addition, two sulfonates with different chain lengths were added consecutively prior to the introduction of hydrogen peroxide to the reaction. The total sulfonate concentration in each reaction well was held constant at 20 mM and the concentrations of the two sulfonates were varied in ratios of 1:7, 1:3, 1:1.7, 1:1, 1.7:1, 3:1, and 7:1. The reported absorbance data in Figure 3 in the main text and Figure S24 in the Supporting Information represent the scan recorded closest to 45 minutes after the addition of sodium borohydride to a reaction. The reported λ_{max} wavelengths represent the maximum absorbance between 465 nm and 1000 nm. The scans ranged from 30 to 80 minutes post-borohydride addition. Each reaction type in Figure 3 represents an average of data from 3-4 replicate reactions. An outlier test was not used in data analysis.

Post-Synthesis Ligand Addition Studies

Post-synthesis ligand addition studies were performed using the Opentrons OT-2 robot interfaced with the UV-Vis plate reader. Silver nanocrystals were initially synthesized with 10 mM sodium 1-butanesulfonate following the standard automated procedure. After the addition of sodium borohydride to the final reaction well, the nanocrystals were allowed to grow for 20 minutes. Sodium alkyl sulfonates of different chain lengths and concentrations were then added to the reaction wells. In syntheses where the sulfonate additive concentration was 10 mM in reaction, 20 μL of 100 mM sodium alkyl sulfonate (C = 1, 4, 5, 6, 7, 8, or 10) was added to an initial reaction volume of 180 μL. A sodium nitrate reaction was also included for comparison and consisted of the post-synthesis addition of 20 μL of 100 mM sodium nitrate. In some experiments, the time of sulfonate addition varied between 20 and 90 minutes after the nanocrystals were synthesized. Reactions were also conducted with post-synthesis additions of 2.5 mM to 20 mM sodium 1-octanesulfonate or sodium nitrate. The initial sodium 1-butanesulfonate nanocrystal volume was 160 μL for these reactions. 5 μL to 40 μL of either 100 mM sodium 1-octanesulfonate or 100 mM sodium nitrate was added post-synthesis, followed by the addition of Milli-Q water to maintain a final volume of 200 μL. Control nanocrystal reactions with no later additions were synthesized at 200 μL volumes and matched the reagent concentrations in the 160 or 180 μL initial nanocrystal reaction volumes. After the final post-synthesis addition was complete in an experiment, the well plate was shaken for 30 seconds. UV-Vis scans of all the reactions in the well plate were then recorded every five minutes for twenty minutes. Absorption spectra in Figure 4 include data from UV-Vis scans that were taken between 28 and 34 minutes after post-synthesis addition was complete. Data for the control reactions (with no later sulfonate addition) shown in Figure 4 are from UV-Vis scans that were taken between 61 and 69 minutes after the addition of sodium borohydride.

Silver nanocrystals were also synthesized with potassium bromide following the Frank et. al. synthesis[5] and used for post-synthesis sulfonate addition studies. Reactions were conducted with the Opentrons OT-2 robot interfaced with the UV-Vis plate reader and followed the standard automated procedure described in the main text, but potassium bromide was used in place of the sulfonate additive. Reactions with no, low (0.5 μM), and high (1.5 μM) potassium bromide concentrations were studied. After the addition of sodium borohydride to the final reaction well, the nanocrystals were allowed to grow for 20 minutes. 20 μL of 100 mM sodium 1-butanesulfonate or sodium 1-octanesulfonate was then added to an initial reaction volume of 180 μL. Control

nanocrystal reactions with no later additions were synthesized at 200 μL volumes and matched the reagent concentrations in the 180 μL initial nanocrystal reaction volumes. After the final post-synthesis addition was complete in an experiment, the well plate was shaken for 30 seconds. UV-Vis scans of all the reactions in the well plate were then recorded every five minutes for twenty minutes. Absorbance spectra were recorded between approximately 20 to 25 minutes after the addition of sulfonate to a reaction. For the control reactions, absorbance spectra were recorded approximately 40 to 45 minutes after the addition of sodium borohydride to the reactions.

For nanocrystals that were purified prior to the post-synthesis sulfonate addition, 10 mM sodium 1-butanesulfonate (C4) nanocrystals were first synthesized in a 96 well-plate using the OT-2 Opentrons robot. 11 reactions were conducted with each at a total volume of 160 μL that consisted of 20 μL of 12.5 mM trisodium citrate, 50 μL of 0.375 mM silver nitrate, 50 μL of 50 mM hydrogen peroxide, and 20 μL of 6.25 mM sodium borohydride. The well-plate was then shaken for 30 seconds (300 rpm, double orbital mix). The resulting nanocrystals were allowed to grow for 20 minutes and then combined in a 2 mL centrifuge tube (1.76 mL of reaction solution) and centrifuged at 21130 rcf for 15 minutes, after which a nanocrystal pellet formed at the bottom of the tube. During this time, the empty reaction wells in the 96 well-plate were rinsed by adding and removing 160 μL of Milli-Q water. The nanocrystal pellet in the centrifuge tube was removed by scraping while aspirating 40 μL into the pipette tip (this was repeated twice to transfer a total volume of 80 μL). The pellet was then resuspended in 1.68 mL of Milli-Q such that the total volume of water and the pellet corresponded to the initial total volume of the 11 reactions (1.76 mL). 160 μL of the purified nanocrystals were then manually pipetted back into each of eight of the original reaction wells. Sodium nitrate, sodium 1-butanesulfonate, or sodium 1-octanesulfonate was added at volumes of 10, 20, 30, or 40 μL to each well from a stock concentration of 100 mM by the robot. The corresponding volume of water needed to bring the total volume to 200 μL was then introduced. A control was also run in which only 40 μL of water was added to bring the volume to 200 μL. The well-plate was then shaken (1 minute, double orbital mix at 300 rpm) and reaction wells were scanned after 20 minutes to record absorbance spectra.

To study the effects on sulfonates on a different system, nanocubes were manually synthesized following the procedure from Zhou et. al.[3] Post-synthetic ligand addition experiments were conducted for purified and unpurified nanocubes. The Opentrons OT-2 robot interfaced with the UV-Vis plate reader was used for these studies. The appropriate amount of Milli-Q water was first added to a reaction well in a 96 well-plate such that the final volume would be 200 μL. This was followed by the addition of 170 μL of nanocube solution and then 0, 10, 20, or 30 μL of 100 mM sodium nitrate, sodium 1-octanesulfonate, or sodium 1-butanesulfonate. The well-plate was then shaken for 30 seconds (300 rpm, double orbital mix) and absorbance spectra were recorded after 20 minutes.

Kinetics of Post-Synthesis Ligand Addition

10 mM sodium 1-butanesulfonate silver nanocrystals were manually synthesized at 2 mL volumes in a 24-well plate. Reagents were added in the same order and at the same concentrations as a standard post-synthesis ligand addition reaction on the Opentrons OT-2 robot: 200 μL of 12.5 mM trisodium citrate, 500 μL of 0.375 mM silver nitrate, 200 μL of 100 mM sodium 1-butanesulfonate, and 500 μL of 50 mM hydrogen peroxide. The well plate was shaken using the UV-Vis plate

reader (at 300 rpm in a double orbital rotation) for 30 seconds. After this, 200 µL of 6.25 mM sodium borohydride was added to a reaction and the plate was shaken again using the same settings. The nanocrystals were then allowed to sit at room temperature for 30 minutes before the addition of 200 µL Milli-Q water and 200 µL of 100 mM sodium 1-octanesulfonate, sodium nitrate, or sodium 1-butanesulfonate. Under room lighting, an iPhone camera and tripod were used to record videos (see attached video files as Supporting Information) of nanocrystal color upon sulfonate addition. When recording videos, shadows and movement were avoided, and a consistent overhead angle was used to capture the well plate. Frames of a reaction were collected every 0.2 to 0.5 seconds for 15 seconds following ligand addition to a reaction. The mobile application Colorimeter was used for image analysis. Nanocrystal colors were identified and measured using the iPhone camera. RGB index values were collected at three different spatial points of the reaction well: one in the middle of the well, one on the left side of the well, and one on the right side of the well. Points were placed in locations that did not have visible air bubbles and averaged for each video frame to determine when the color transitions occurred.

Kinetics studies attempted using the Opentrons OT-2 robot and integrated UV-Visible plate reader for post-synthesis sulfonate addition reactions were unsuccessful because the change in the color of the nanocrystals occurred too quickly to be captured by the plate reader. Furthermore, meaningful kinetics data could not be obtained when using a fiber optic dip probe and Cary UV-Vis system for reactions at scaled up volumes, likely because of diffusion effects on nanocrystal synthesis.

Supplemental Results & Figures

Stirring Study

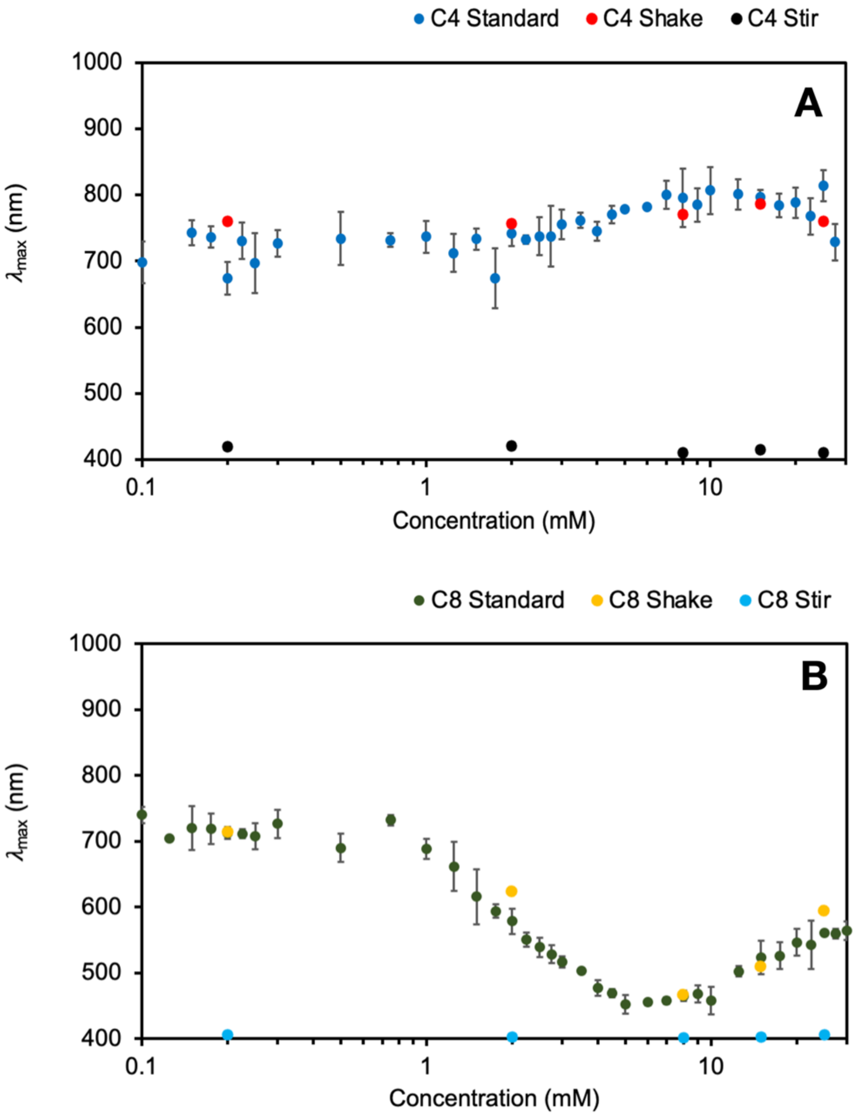

Figure S1. Effects of mixing conditions on silver nanocrystal formation. **A)** Comparison of λ_{max} for 0.1 mM to 30 mM sodium 1-butanesulfonate nanocrystals synthesized with the standard automated procedure at 200 μL volumes (dark blue) and manually at 1 mL volumes, where reactions were mixed by shaking on the plate reader (red) or with continuous stirring (black); **B)** Comparison of λ_{max} for 0.1 mM to 30 mM sodium 1-octanesulfonate nanocrystals synthesized with the standard automated procedure at 200 μL volumes (green) and manually at 1 mL volumes, where reactions were mixed by shaking on the plate reader (yellow) or continuous stirring (light blue). Data points for the scaled up, manually synthesized nanocrystals are representative of one reaction.

The resulting λ_{max} for manually synthesized silver nanocrystals was affected by the mixing conditions. 1 mL reactions with sodium 1-butanesulfonate or sodium 1-octanesulfonate that were continuously stirred at 350 rpm resulted in no silver nanocrystal formation. However, when the mixing conditions of the standard automated synthesis were applied to the scaled-up 1 mL manual syntheses, the resulting λ_{max} were within or near the error bars of the λ_{max} data from automated reactions. The discrepancy in nanocrystal reaction outcomes resulting from the type of mixing could be attributed to differences in reactant diffusion. Further studies are necessary to better understand the effects of different mixing methods on the reaction.

Opentrons OT-2 Robot Experimental Set-Up

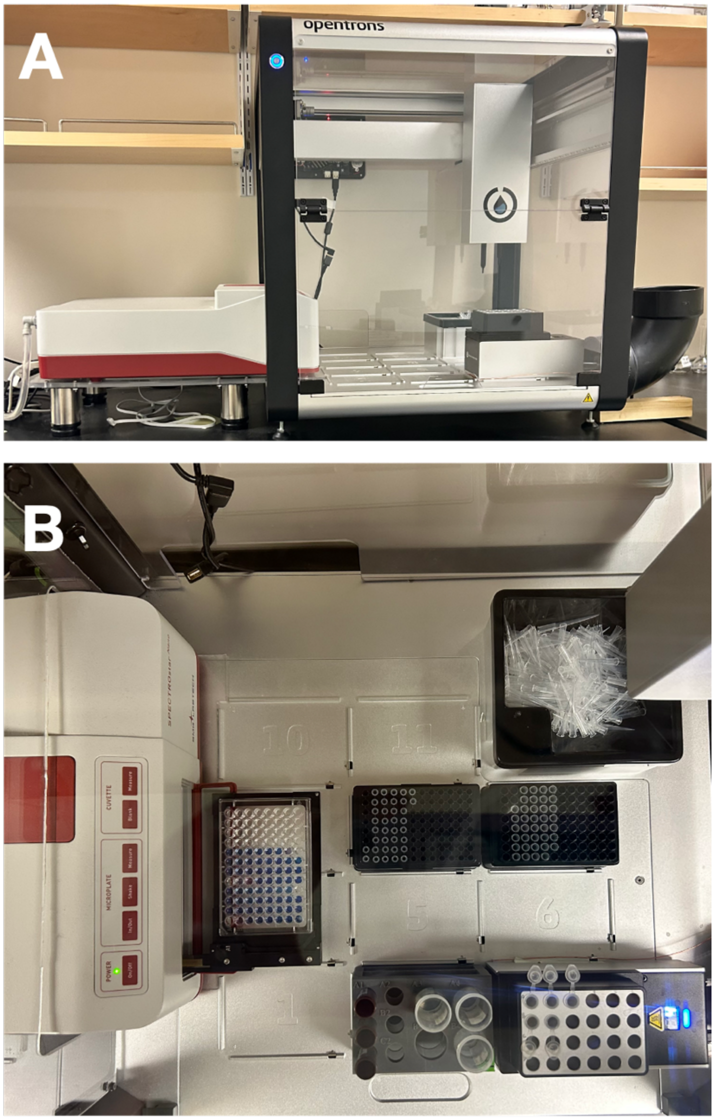

Figure S2. Opentrons OT-2 liquid-handling robot experimental set-up for the automated synthesis of silver nanocrystals. **A)** Opentrons OT-2 liquid-handling robot integrated with a SPECTROstar Nano plate reader from BMG Labtech; **B)** Deck set-up with 20 µL and 300 µL pipette tips, custom-built tube holder with reagents, 96-well plate on ejected plate holder, and temperature controller set to 4 °C with cooled reagents. Software and hardware for the interfaced system is described in Martin et al., 2022.[6]

Kinetics of Alkyl Sulfonate Nanocrystal Synthesis

Sodium 1-butanesulfonate Nanocrystals

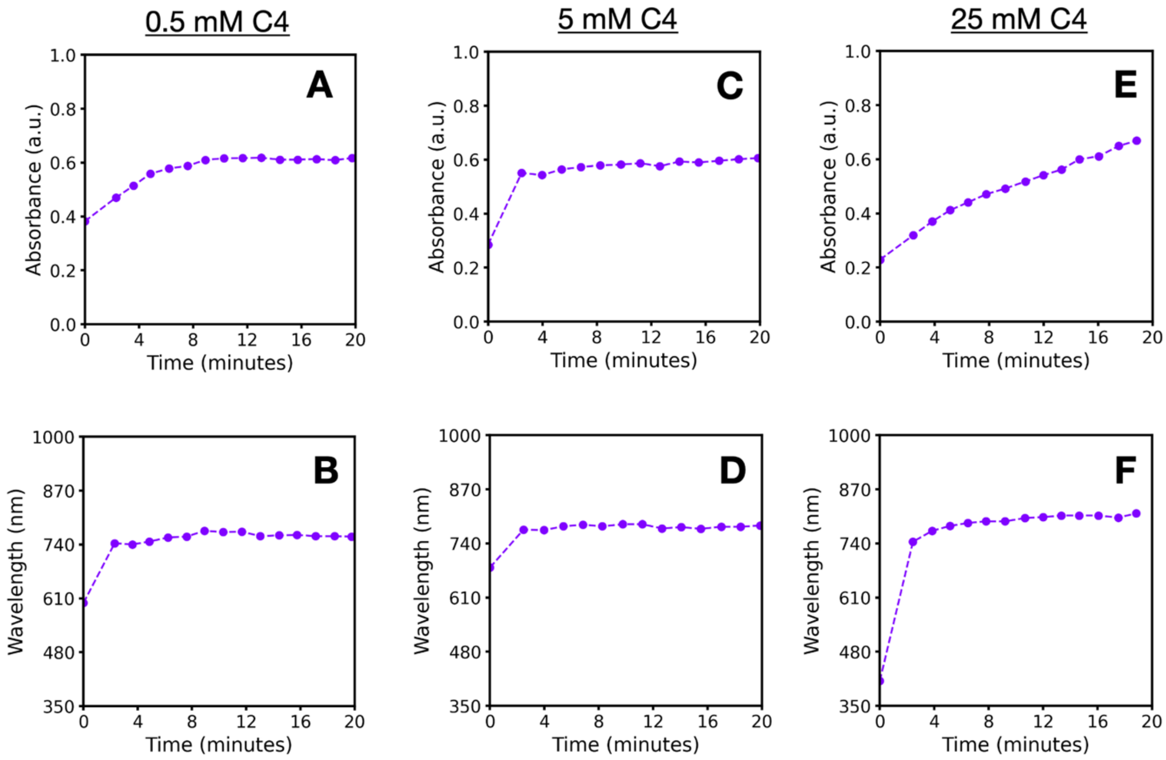

Figure S3. Growth kinetics of silver nanocrystals with varying concentrations of sodium 1-butanesulfonate. Maximum absorbance (top row) and λ_{max} (bottom row) data are shown for the first 20 minutes of nanocrystal growth. **A-B)** nanocrystals with 0.5 mM sodium 1-butanesulfonate; **C-D)** nanocrystals with 5 mM sodium 1-butanesulfonate; **E-F)** nanocrystals with 25 mM sodium 1-butanesulfonate.

Sodium 1-hexanesulfonate Nanocrystals

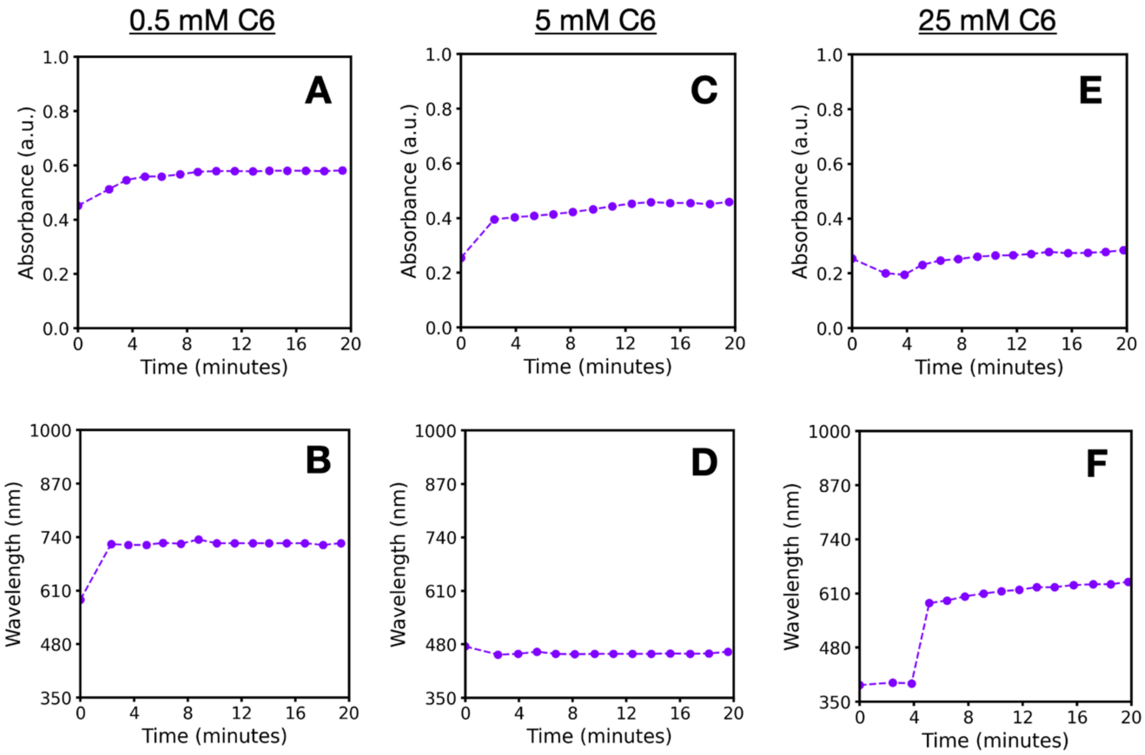

Figure S4. Growth kinetics of silver nanocrystals with varying concentrations of sodium 1-hexanesulfonate. Maximum absorbance (top row) and λ_{max} (bottom row) data are shown for the first 20 minutes of nanocrystal growth. **A-B)** nanocrystals with 0.5 mM sodium 1-hexanesulfonate; **C-D)** nanocrystals with 5 mM sodium 1-hexanesulfonate; **E-F)** nanocrystals with 25 mM sodium 1-hexanesulfonate.

Sodium 1-octanesulfonate Nanocrystals

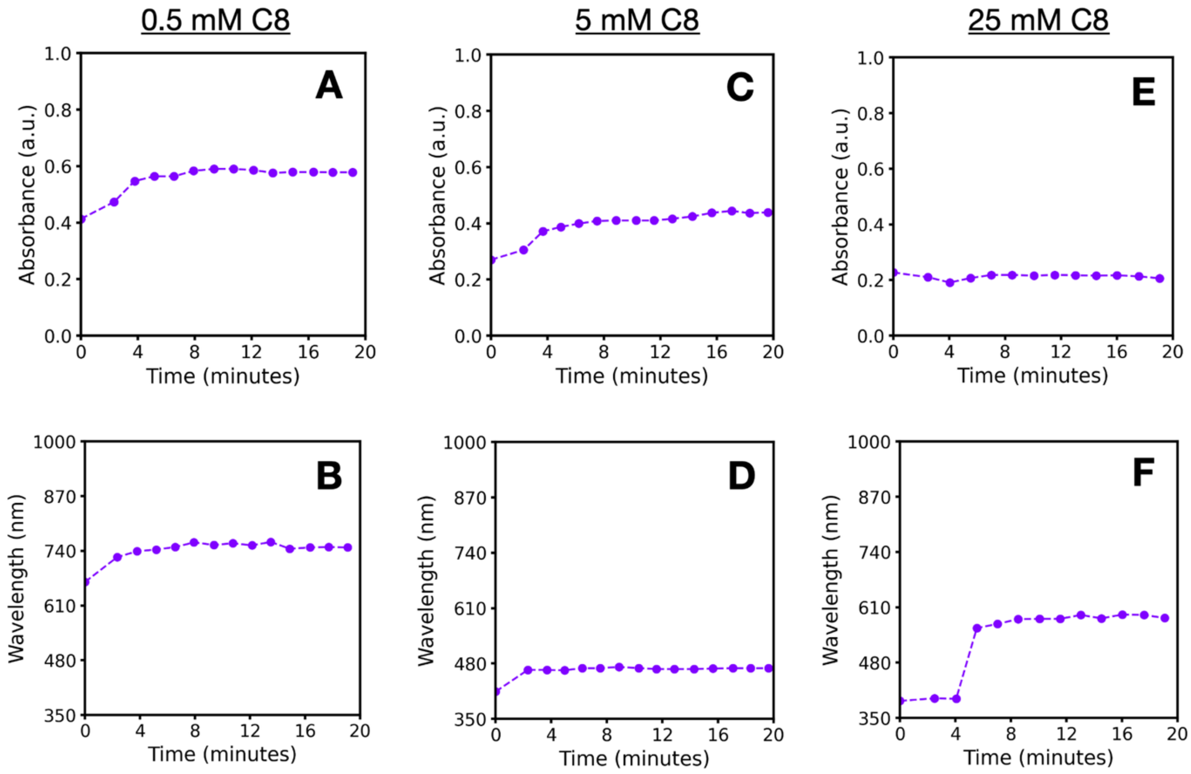

Figure S5. Growth kinetics of silver nanocrystals with varying concentrations of sodium 1-octanesulfonate. Maximum absorbance (top row) and λ_{max} (bottom row) data are shown for the first 20 minutes of nanocrystal growth. **A-B)** nanocrystals with 0.5 mM sodium 1-octanesulfonate; **C-D)** nanocrystals with 5 mM sodium 1-octanesulfonate; **E-F)** nanocrystals with 25 mM sodium 1-octanesulfonate.

Sodium 1-decanesulfonate Nanocrystals

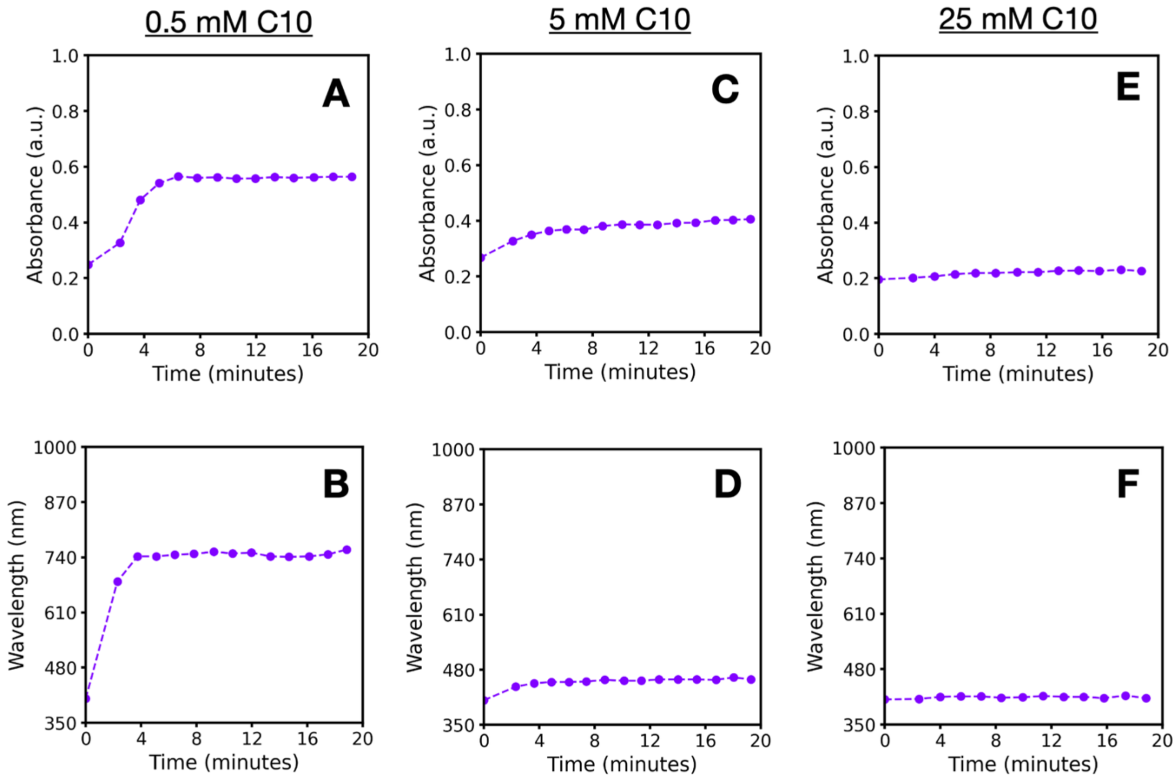

Figure S6. Growth kinetics of silver nanocrystals with varying concentrations of sodium 1-decanesulfonate. Maximum absorbance (top row) and λ_{max} (bottom row) data are shown for the first 20 minutes of nanocrystal growth. **A-B)** nanocrystals with 0.5 mM sodium 1-decanesulfonate; **C-D)** nanocrystals with 5 mM sodium 1-decanesulfonate; **E-F)** nanocrystals with 25 mM sodium 1-decanesulfonate.

Alkyl Sulfonate Data

Table S1. Data for silver nanocrystals synthesized with no sulfonate or sodium nitrate (0.1 mM to 30 mM).

Concentration (mM)	Average Lambda Max Wavelength (nm)	Standard Deviation of Lambda Max Wavelength	Average Lambda Max Absorbance (a.u.)	Standard Deviation of Lambda Max Absorbance	Number of Reactions
0	733.3	33.1	0.544	0.032	12
0.1	759.0	19.2	0.654	0.037	4
0.15	756.9	12.5	0.574	0.068	6
0.175	767.2	20.9	0.576	0.054	7
0.2	763.7	21.3	0.572	0.065	7
0.225	762.8	8.4	0.588	0.054	6
0.3	761.5	24.4	0.580	0.048	7
0.5	740.8	13.1	0.580	0.067	6
0.75	742.0	4.6	0.578	0.059	6
1	750.3	17.0	0.586	0.066	6
1.25	743.4	11.5	0.567	0.025	6
1.5	759.5	20.3	0.577	0.076	7
1.75	780.9	26.5	0.594	0.053	7
2	773.9	24.9	0.570	0.044	10
2.25	783.9	20.6	0.610	0.057	7
2.5	777.1	38.3	0.615	0.059	7
2.75	745.1	53.2	0.524	0.083	22
3	778.5	42.9	0.624	0.048	7
3.5	774.1	12.5	0.604	0.075	6
4	777.4	6.9	0.586	0.059	6
4.5	783.4	40.7	0.591	0.071	7
6	794.3	30.6	0.581	0.051	7
7	788.8	32.2	0.584	0.055	7
8	784.5	39.1	0.566	0.094	7
9	795.1	28.1	0.608	0.098	7
12.5	825.0	26.2	0.628	0.050	7
15	827.6	31.9	0.640	0.059	7
17.5	832.0	35.8	0.689	0.159	7
20	828.1	43.8	0.577	0.102	11
22.5	842.7	40.0	0.640	0.076	7
25	834.9	44.5	0.501	0.135	17
27.5	830.2	46.5	0.562	0.101	10
30	824.9	38.5	0.526	0.115	7

Table S2. Data for silver nanocrystals synthesized with 0.1 mM to 30 mM sodium 1-methanesulfonate (C1).

Concentration (mM)	Average Lambda Max Wavelength (nm)	Standard Deviation of Lambda Max Wavelength	Average Lambda Max Absorbance (a.u.)	Standard Deviation of Lambda Max Absorbance	Number of Reactions
0.15	735.2	34.3	0.588	0.056	6
0.175	732.6	40.7	0.563	0.037	6
0.2	743.6	41.4	0.572	0.047	6
0.225	744.3	30.1	0.579	0.042	6
0.3	743.4	40.8	0.583	0.040	6
0.5	745.8	26.8	0.570	0.038	6
0.75	741.5	38.5	0.568	0.038	6
1	756.2	49.5	0.586	0.047	6
1.25	749.1	35.3	0.606	0.042	6
1.5	762.9	35.0	0.587	0.048	6
1.75	797.5	18.6	0.628	0.024	4
2	789.7	46.2	0.628	0.045	6
2.25	770.4	38.8	0.612	0.056	6
2.5	788.0	22.7	0.626	0.034	6
2.75	774.4	40.8	0.620	0.042	6
3	779.0	24.5	0.617	0.034	6
3.5	804.0	9.8	0.637	0.031	6
4	792.1	17.0	0.614	0.038	6
4.5	805.2	12.5	0.633	0.016	6
6	834.2	8.1	0.646	0.023	6
7	828.9	21.1	0.659	0.026	6
8	843.4	19.7	0.671	0.021	6
9	843.3	20.8	0.689	0.039	6
12.5	867.1	19.1	0.680	0.019	6
15	870.5	26.9	0.689	0.048	6
17.5	895.8	13.9	0.685	0.097	5
20	890.0	25.7	0.683	0.022	6
22.5	892.2	7.8	0.658	0.020	6
25	883.0	17.1	0.628	0.053	5
27.50	865.5	31.4	0.540	0.062	6
30	861.3	32.0	0.526	0.103	6

Table S3. Data for silver nanocrystals synthesized with 0.1 mM to 30 mM sodium 1-butanesulfonate (C4).

Concentration (mM)	Average Lambda Max Wavelength (nm)	Standard Deviation of Lambda Max Wavelength	Average Lambda Max Absorbance (a.u.)	Standard Deviation of Lambda Max Absorbance	Number of Reactions
0.1	730.0	41.8	0.573	0.030	4
0.15	764.5	25.2	0.604	0.025	5
0.175	763.1	18.8	0.604	0.064	4
0.2	745.1	42.2	0.589	0.077	8
0.225	778.7	23.3	0.649	0.036	5
0.25	760.4	44.1	0.607	0.071	7
0.3	778.9	24.8	0.644	0.035	5
0.5	744.1	35.9	0.629	0.070	5
0.75	742.1	14.4	0.600	0.025	5
1	753.1	31.6	0.596	0.035	5
1.25	741.9	29.9	0.605	0.016	4
1.5	760.8	22.7	0.656	0.103	5
1.75	742.5	46.5	0.585	0.063	8
2	774.4	30.7	0.610	0.049	5
2.25	755.0	19.4	0.601	0.059	4
2.5	756.9	33.9	0.596	0.061	7
2.75	766.0	42.6	0.619	0.054	4
3	781.0	30.1	0.611	0.064	5
3.5	774.8	19.3	0.601	0.019	5
4	765.5	19.1	0.611	0.033	5
4.5	783.2	14.9	0.627	0.027	5
5	789.1	6.8	0.641	0.024	4
6	778.9	28.7	0.606	0.061	5
7	789.3	18.2	0.639	0.023	4
8	804.5	39.4	0.646	0.062	5
9	798.8	26.6	0.650	0.039	5
10	786.7	7.2	0.578	0.075	3
12.5	801.1	29.6	0.641	0.034	5
15	807.6	7.2	0.677	0.107	5
17.5	799.3	13.5	0.630	0.029	4
20	805.1	16.5	0.638	0.022	5
22.5	795.0	18.4	0.616	0.051	5
25	801.0	31.4	0.565	0.087	6
27.5	770.6	27.6	0.534	0.053	5
30	750.2	2.5	0.459	0.119	4

Table S4. Data for silver nanocrystals synthesized with 0.1 mM to 30 mM sodium 1-pentanesulfonate (C5).

Concentration (mM)	Average Lambda Max Wavelength (nm)	Standard Deviation of Lambda Max Wavelength	Average Lambda Max Absorbance (a.u.)	Standard Deviation of Lambda Max Absorbance	Number of Reactions
0.1	739.5	23.8	0.600	0.023	4
0.15	723.8	27.9	0.580	0.046	3
0.175	720.9	25.6	0.579	0.033	4
0.2	724.3	32.9	0.553	0.030	4
0.225	730.4	32.9	0.566	0.030	4
0.3	717.0	37.6	0.562	0.039	4
0.5	732.8	38.8	0.557	0.032	4
0.75	726.3	36.4	0.553	0.031	4
1	728.9	36.0	0.539	0.020	4
1.25	708.8	15.5	0.538	0.012	3
1.5	669.1	21.3	0.498	0.020	4
1.75	636.7	21.1	0.488	0.033	4
2	615.5	16.6	0.474	0.032	4
2.25	598.6	5.2	0.437	0.051	3
2.5	573.8	17.7	0.468	0.006	4
2.75	555.7	1.5	0.462	0.012	3
3	561.6	2.0	0.485	0.120	3
3.5	521.6	12.2	0.414	0.031	4
4	520.9	1.1	0.420	0.018	3
4.5	515.2	2.4	0.413	0.030	3
6	495.3	11.9	0.416	0.018	4
7	501.6	3.9	0.408	0.011	3
8	506.5	10.5	0.387	0.026	4
9	516.2	24.5	0.371	0.051	4
12.5	552.9	13.2	0.348	0.024	4
15	615.3	3.8	0.314	0.022	3
17.5	605.1	22.5	0.327	0.044	4
20	634.3	16.6	0.330	0.051	4
22.5	649.7	11.9	0.314	0.019	4
25	647.7	15.8	0.295	0.053	4
27.5	655.1	12.6	0.278	0.030	4
30	644.3	27.6	0.217	0.025	4

Table S5. Data for silver nanocrystals synthesized with 0.1 mM to 30 mM sodium 1-hexanesulfonate (C6).

Concentration (mM)	Average Lambda Max Wavelength (nm)	Standard Deviation of Lambda Max Wavelength	Average Lambda Max Absorbance (a.u.)	Standard Deviation of Lambda Max Absorbance	Number of Reactions
0.1	745.4	25.3	0.581	0.041	4
0.125	740.1	19.8	0.575	0.034	6
0.15	753.0	40.2	0.639	0.100	3
0.175	735.9	24.5	0.574	0.001	3
0.2	729.1	15.1	0.570	0.016	3
0.225	735.0	28.5	0.568	0.013	3
0.25	765.8	9.3	0.583	0.023	4
0.3	749.2	43.0	0.568	0.019	4
0.5	736.6	16.6	0.575	0.022	4
0.75	733.9	18.6	0.555	0.012	4
1	668.0	30.9	0.514	0.015	4
1.25	649.5	16.9	0.514	0.012	5
1.5	607.8	5.5	0.502	0.022	3
1.75	587.2	23.2	0.493	0.017	6
2	570.4	22.6	0.474	0.014	4
2.25	531.7	8.5	0.446	0.008	3
2.5	511.8	8.8	0.439	0.021	4
2.75	520.7	14.6	0.447	0.012	3
3	515.4	6.7	0.440	0.007	4
3.5	502.3	2.3	0.421	0.017	4
4	494.7	2.3	0.435	0.011	3
4.5	477.5	3.3	0.410	0.025	4
5	478.0	1.0	0.391	0.021	3
6	478.5	2.2	0.410	0.010	3
7	485.6	12.3	0.410	0.010	4
8	502.8	10.6	0.396	0.035	4
9	518.6	4.6	0.377	0.010	4
10	527.5	19.0	0.338	0.031	4
12.5	552.6	29.5	0.351	0.039	5
15	583.2	32.8	0.342	0.022	3
17.5	642.7	7.0	0.346	0.027	5
20	649.5	38.4	0.327	0.030	5
22.5	643.4	25.2	0.330	0.032	3
25	661.1	25.4	0.291	0.041	4
27.5	671.4	18.7	0.260	0.074	4
30	661.5	13.3	0.221	0.080	4

Table S6. Data for silver nanocrystals synthesized with 0.1 mM to 30 mM sodium 1-heptanesulfonate (C7).

Concentration (mM)	Average Lambda Max Wavelength (nm)	Standard Deviation of Lambda Max Wavelength	Average Lambda Max Absorbance (a.u.)	Standard Deviation of Lambda Max Absorbance	Number of Reactions
0.1	733.3	6.2	0.584	0.013	4
0.15	747.7	7.5	0.594	0.020	5
0.175	756.9	17.2	0.597	0.028	4
0.2	769.3	16.5	0.613	0.021	4
0.225	731.6	2.0	0.582	0.007	3
0.3	753.1	17.1	0.587	0.036	5
0.5	740.9	22.0	0.583	0.027	5
0.75	685.1	31.1	0.534	0.027	4
1	643.7	23.1	0.499	0.033	5
1.25	601.7	36.8	0.458	0.057	4
1.5	549.7	16.4	0.427	0.035	5
1.75	546.3	21.7	0.435	0.031	4
2	523.2	15.5	0.425	0.023	5
2.25	510.6	10.4	0.426	0.027	4
2.5	504.0	7.2	0.434	0.007	5
2.75	496.7	4.3	0.408	0.016	5
3	500.9	4.4	0.391	0.014	5
3.5	482.7	10.0	0.408	0.029	5
4	471.3	10.8	0.410	0.019	5
4.5	478.2	9.7	0.399	0.031	5
6	484.2	10.7	0.388	0.021	5
7	499.7	3.6	0.374	0.035	3
8	503.5	8.0	0.347	0.030	5
9	518.2	4.7	0.338	0.014	5
12.5	549.5	8.1	0.297	0.024	5
15	572.6	14.7	0.245	0.056	5
17.5	580.1	16.3	0.262	0.021	5
20	601.1	18.7	0.258	0.032	4
22.5	618.3	16.5	0.244	0.045	5
25	620.1	18.3	0.230	0.032	5
27.5	619.2	14.4	0.193	0.011	5
30	615.7	16.0	0.146	0.015	5

Table S7. Data for silver nanocrystals synthesized with 0.1 mM to 30 mM sodium 1-octanesulfonate (C8).

Concentration (mM)	Average Lambda Max Wavelength (nm)	Standard Deviation of Lambda Max Wavelength	Average Lambda Max Absorbance (a.u.)	Standard Deviation of Lambda Max Absorbance	Number of Reactions
0.1	750.6	14.4	0.564	0.027	4
0.125	766.7	31.4	0.608	0.052	5
0.15	745.6	30.3	0.620	0.076	6
0.175	744.4	24.6	0.593	0.036	4
0.2	726.3	13.3	0.556	0.017	3
0.225	727.1	14.3	0.547	0.008	3
0.25	738.8	33.9	0.588	0.068	4
0.3	769.2	21.9	0.610	0.044	5
0.5	702.4	19.9	0.529	0.016	5
0.75	738.5	18.1	0.570	0.020	5
1	699.8	13.6	0.524	0.022	4
1.25	672.1	47.3	0.510	0.022	3
1.5	622.6	28.0	0.490	0.030	6
1.75	605.8	15.7	0.483	0.008	3
2	585.2	20.9	0.471	0.025	5
2.25	553.0	13.3	0.446	0.037	3
2.5	549.8	7.9	0.448	0.026	6
2.75	539.4	4.1	0.459	0.008	4
3	517.8	5.9	0.427	0.015	6
3.5	500.9	8.2	0.428	0.016	4
4	480.5	11.2	0.433	0.008	4
4.5	476.5	9.1	0.438	0.020	6
5	466.6	17.5	0.429	0.013	4
6	458.2	6.9	0.423	0.031	4
7	452.9	10.9	0.435	0.017	6
8	468.3	7.6	0.412	0.016	4
9	474.6	14.6	0.380	0.015	4
10	481.6	29.4	0.408	0.016	4
12.5	520.4	20.4	0.346	0.018	6
15	546.6	31.6	0.318	0.025	6
17.5	540.7	18.3	0.318	0.010	4
20	560.6	22.2	0.315	0.005	4
22.5	571.6	22.8	0.298	0.014	4
25	563.5	3.6	0.294	0.016	5
27.5	586.6	18.9	0.270	0.017	6
30	595.2	28.0	0.226	0.053	6

Effect of High Sulfonate Concentration on Nanocrystal Absorbance

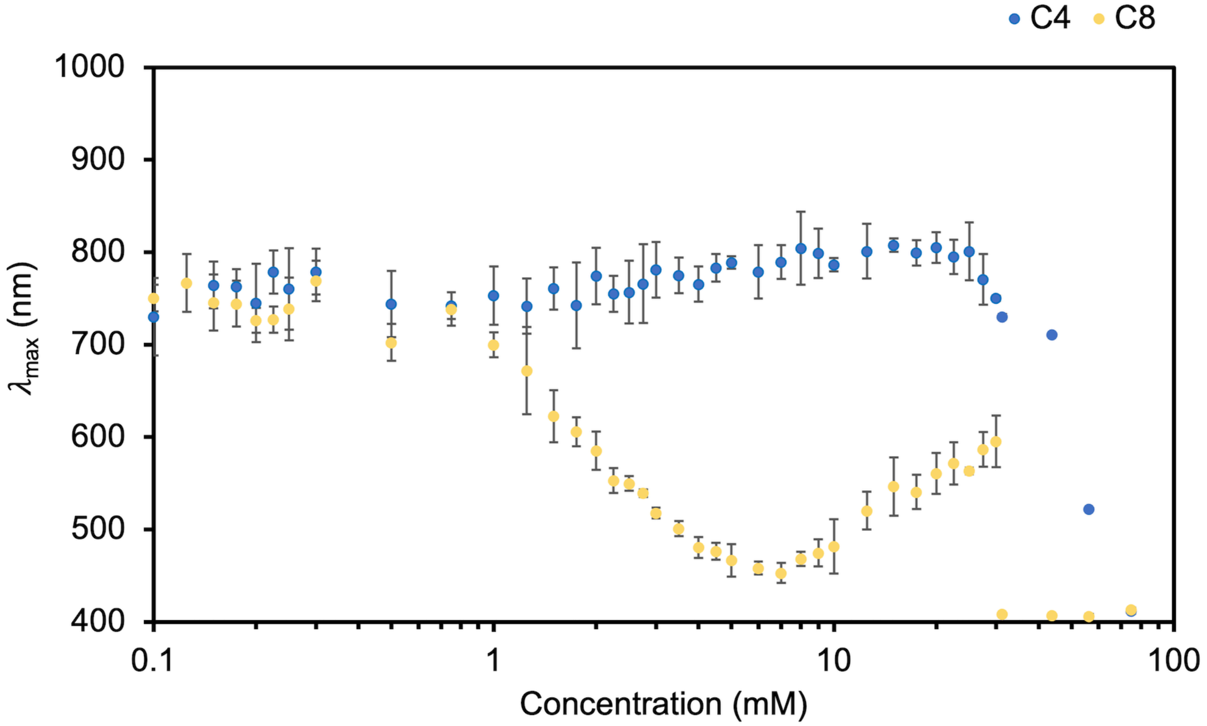

Figure S7. λ_{max} for silver nanocrystals synthesized with in-reaction concentrations of 0.1 mM to 75 mM sodium 1-butanesulfonate (blue) or sodium 1-octanesulfonate (yellow) plotted on a logarithmic scale. Error bars represent one standard deviation (where not visible, the bars are within the size of the data points). Data points with sulfonate concentrations of 31.25 mM, 43.8 mM, 56.3 mM, and 75 mM represent the average from two reactions. All absorbance data represents the scan recorded closest to 45 minutes after the addition of sodium borohydride to a reaction. These scans ranged from 30 to 80 minutes post-borohydride addition.

Effect of High Sulfonate Concentration on Nanocrystal Growth Kinetics

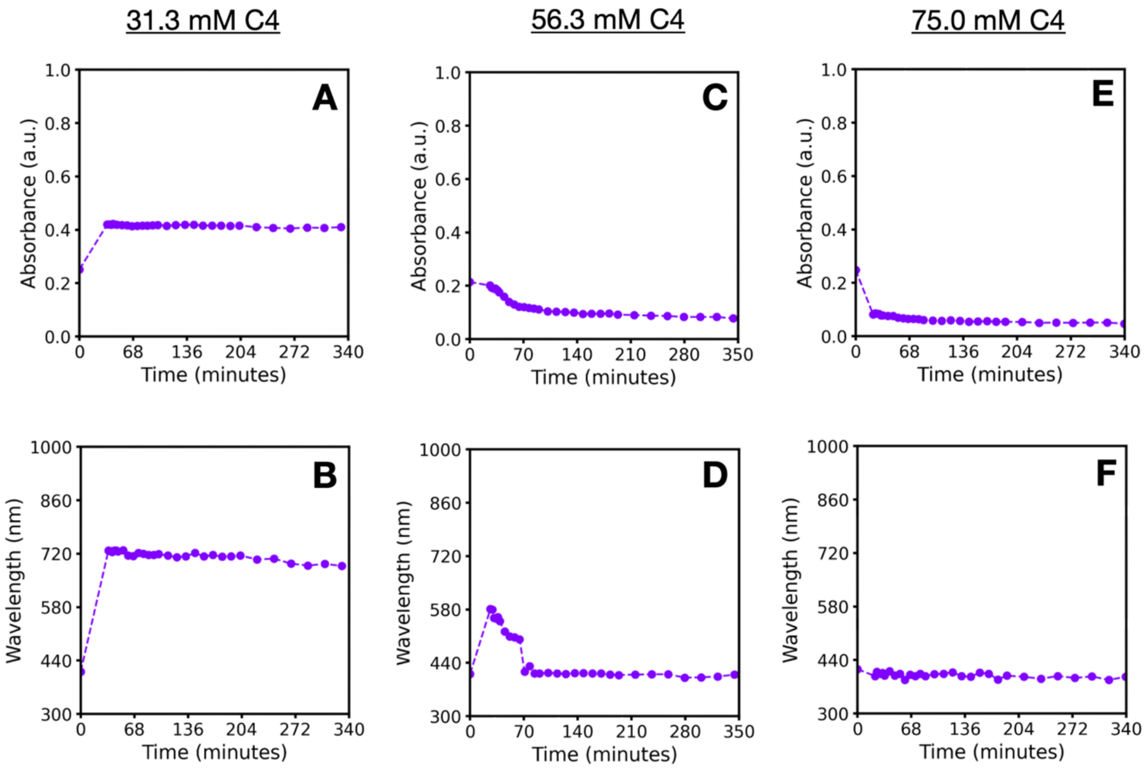

Figure S8. Long-term growth kinetics of 31.3 mM, 56.3 mM, and 75.0 mM sodium 1-butanesulfonate nanocrystals from 340-350 minutes post-borohydride addition. **A)** Maximum absorbance values of 31.3 mM sodium 1-butanesulfonate nanocrystals over time. **B)** λ_{max} values of 31.3 mM sodium 1-butanesulfonate nanocrystals over time. **C)** Maximum absorbance values of 56.3 mM sodium 1-butanesulfonate nanocrystals over time. **D)** λ_{max} values of 56.3 mM sodium 1-butanesulfonate nanocrystals over time. **E)** Maximum absorbance values of 75.0 mM sodium 1-butanesulfonate nanocrystals over time. **F)** λ_{max} values of 75.0 mM sodium 1-butanesulfonate nanocrystals over time.

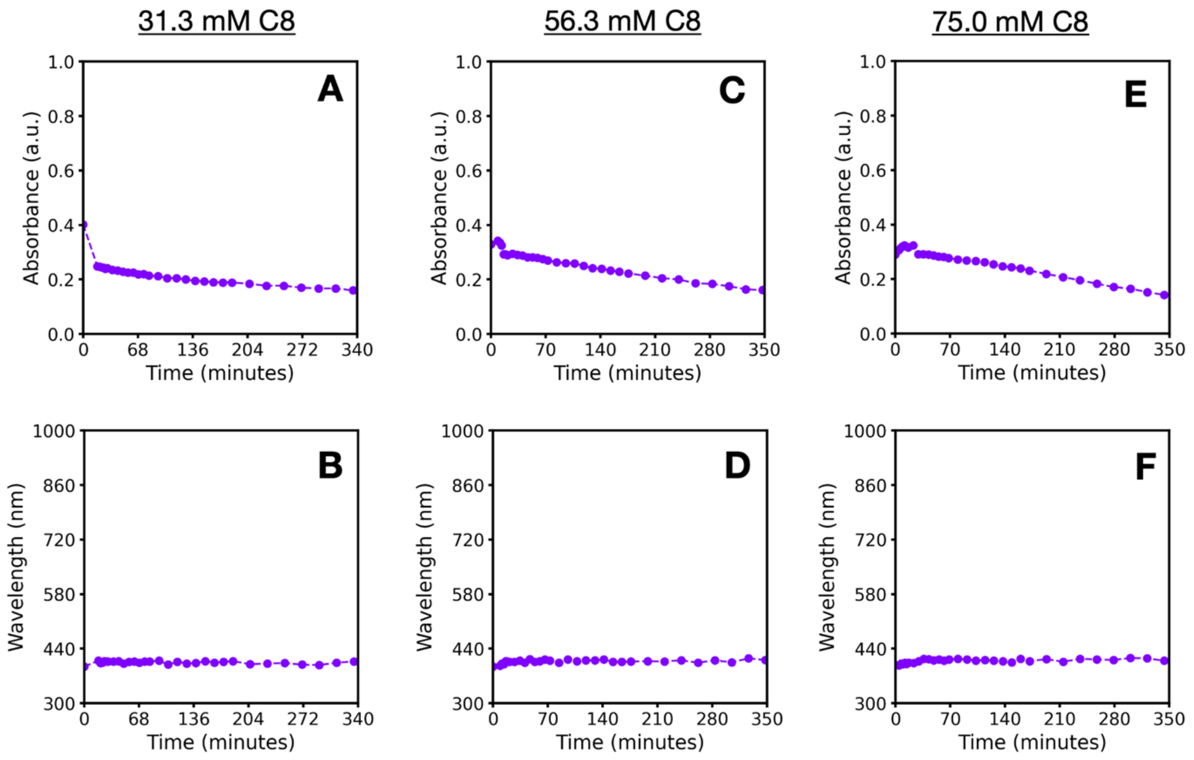

Figure S9. Long-term growth kinetics of 31.3 mM, 56.3 mM, and 75.0 mM sodium 1-octanesulfonate nanocrystals from 340-350 minutes post-borohydride addition. **A)** Maximum absorbance values of 31.3 mM sodium 1-octanesulfonate nanocrystals over time. **B)** λ_{max} values of 31.3 mM sodium 1-octanesulfonate nanocrystals over time. **C)** Maximum absorbance values of 56.3 mM sodium 1-octanesulfonate nanocrystals over time. **D)** λ_{max} values of 56.3 mM sodium 1-octanesulfonate nanocrystals over time. **E)** Maximum absorbance values of 75.0 mM sodium 1-octanesulfonate nanocrystals over time. **F)** λ_{max} values of 75.0 mM sodium 1-octanesulfonate nanocrystals over time.

Silver Nanocrystal Long-Term Kinetics Studies

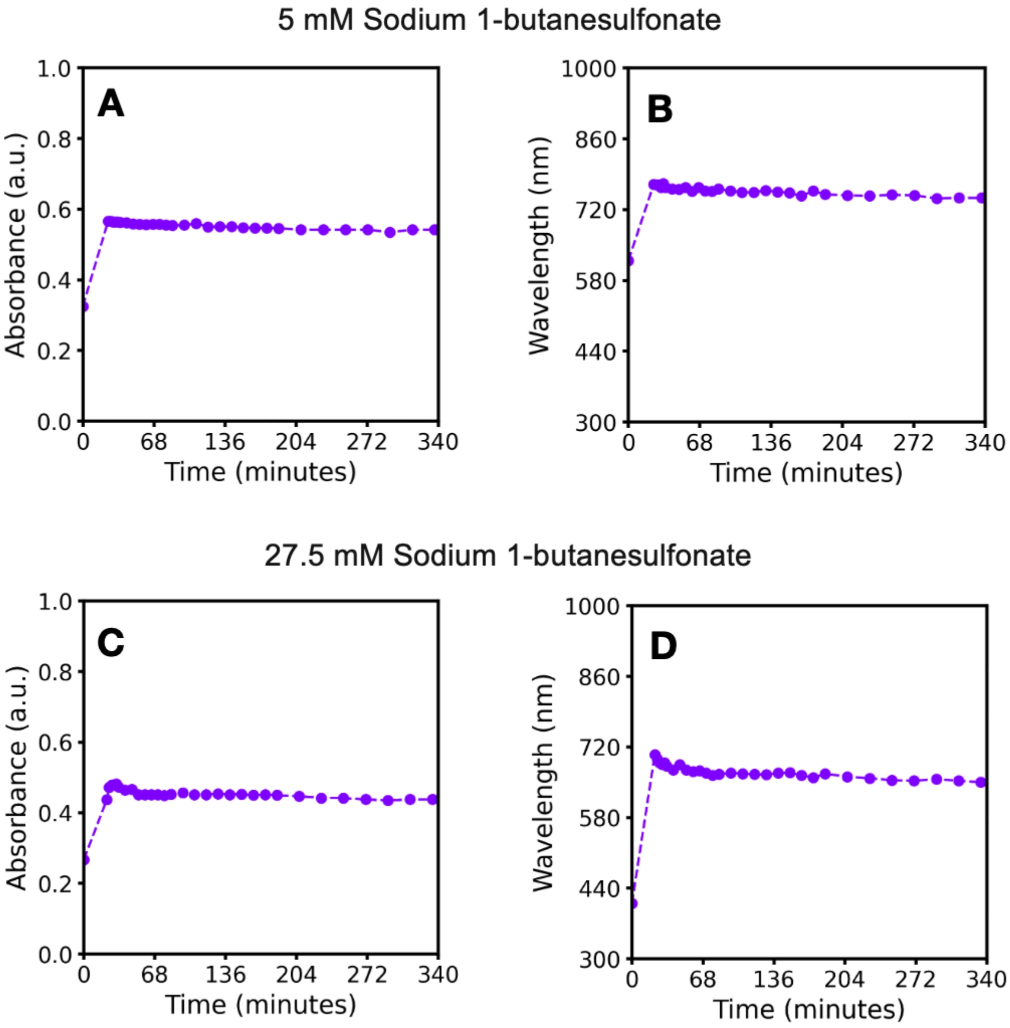

Figure S10. Long-term growth kinetics of 5 mM and 27.5 mM sodium 1-butanesulfonate nanocrystals over a period of 340 minutes post-borohydride addition. **A)** Maximum absorbance values of 5 mM sodium 1-butanesulfonate nanocrystals over time. **B)** λ_{max} values of 5 mM sodium 1-butanesulfonate nanocrystals over time. **C)** Maximum absorbance values of 27.5 mM sodium 1-butanesulfonate nanocrystals over time. **D)** λ_{max} values of 27.5 mM sodium 1-butanesulfonate nanocrystals over time.

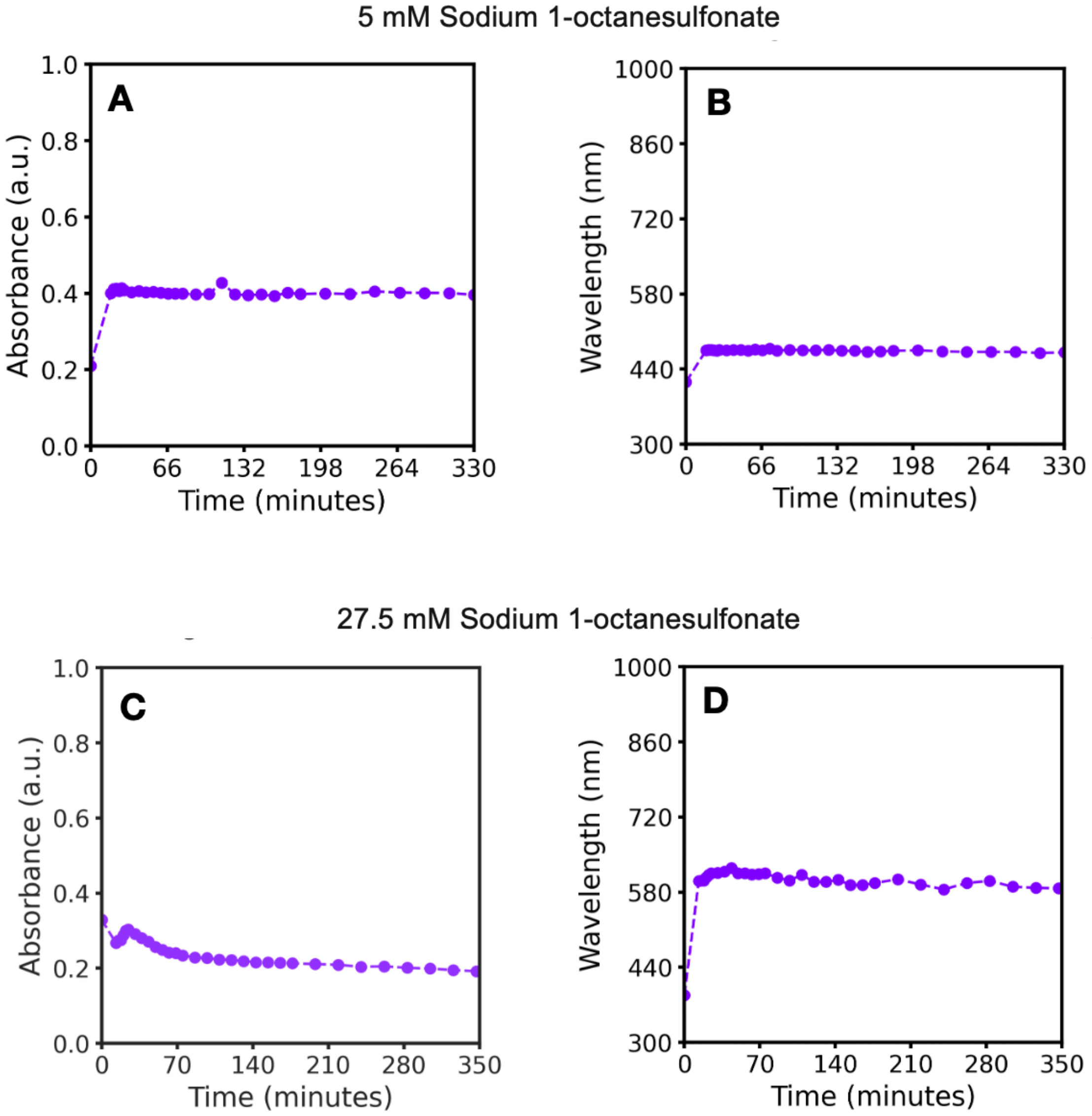

Figure S11. Long-term growth kinetics of 5 mM and 27.5 mM sodium 1-octanesulfonate nanocrystals over a period of 330 minutes to 350 minutes post-borohydride addition. **A)** Maximum absorbance values of 5 mM sodium 1-octanesulfonate nanocrystals over time. **B)** λ_{max} values of 5 mM sodium 1-octanesulfonate nanocrystals over time. **C)** Maximum absorbance values of 27.5 mM sodium 1-octanesulfonate nanocrystals over time. **D)** λ_{max} values of 27.5 mM sodium 1-octanesulfonate nanocrystals over time.

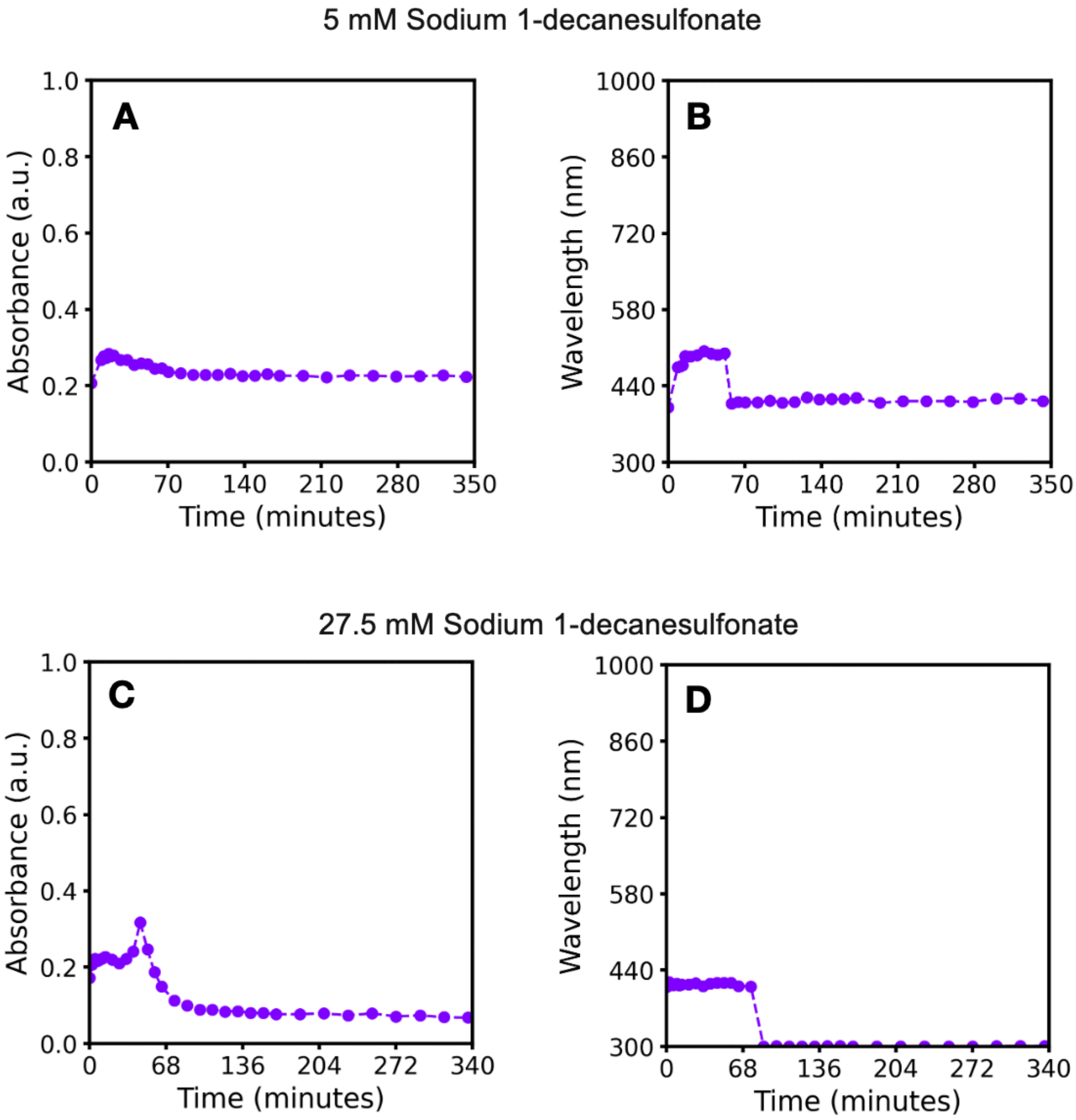

Figure S12. Long-term growth kinetics of 5 mM and 27.5 mM sodium 1-decanesulfonate nanocrystals over a period of 340 minutes after sodium borohydride addition. **A)** Maximum absorbance values of 5 mM sodium 1-decanesulfonate nanocrystals over time. **B)** λ_{max} values of 5 mM sodium 1-decanesulfonate nanocrystals over time. **C)** Maximum absorbance values of 27.5 mM sodium 1-decanesulfonate nanocrystals over time. **D)** λ_{max} values of 27.5 mM sodium 1-decanesulfonate nanocrystals over time.

Polystyrene Sulfonate Nanocrystal Synthesis

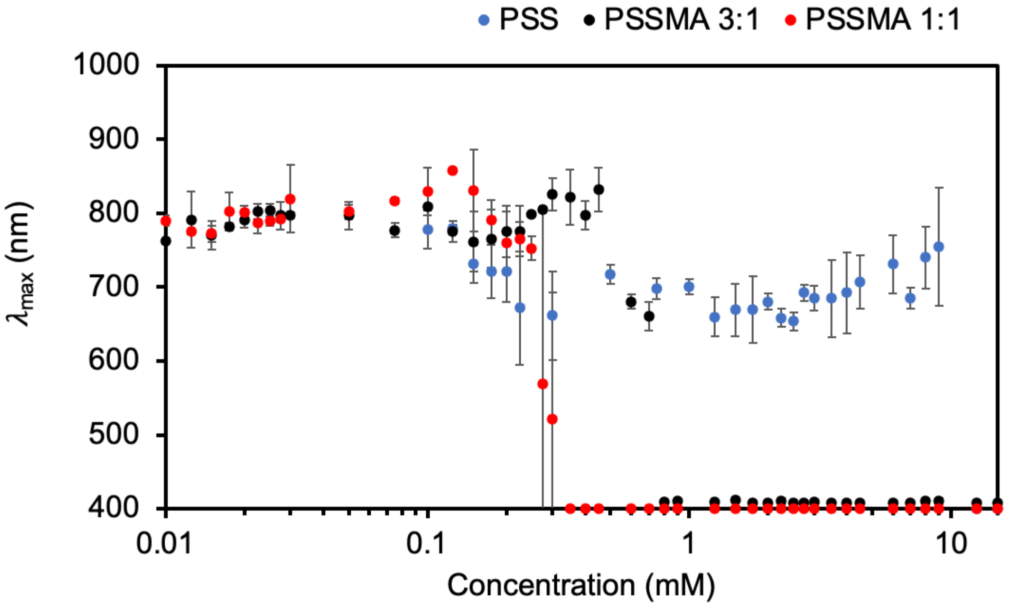

Figure S13. λ_{max} values for silver nanocrystals synthesized with varying concentrations of poly(sodium 4-styrenesulfonate) (PSS, blue), poly(4-styrenesulfonic acid-co-maleic acid) sodium salt with 1:1 4-styrenesulfonic acid:maleic acid mol ratio (PSSMA 1:1, red), and poly(4-styrenesulfonic acid-co-maleic acid) sodium salt with 3:1 4-styrenesulfonic acid:maleic acid mol ratio (PSSMA 3:1, black). The λ_{max} for nanocrystals with PSSMA 3:1 at concentrations above 0.7 mM was increased by 8 nm to make the points visible because of overlap with the PSSMA 1:1 data. Reaction wells were scanned between 15 and 239 minutes after the addition of sodium borohydride. Points with error bars represent the standard deviation of averaged trials when applicable. λ_{max} data for nanocrystals synthesized with PSSMA 1:1 at concentrations above 1.5 mM is representative of one reaction; otherwise, error bars are within the size of the data points when not visible.

Micelle Studies Using Fluorescence Spectroscopy

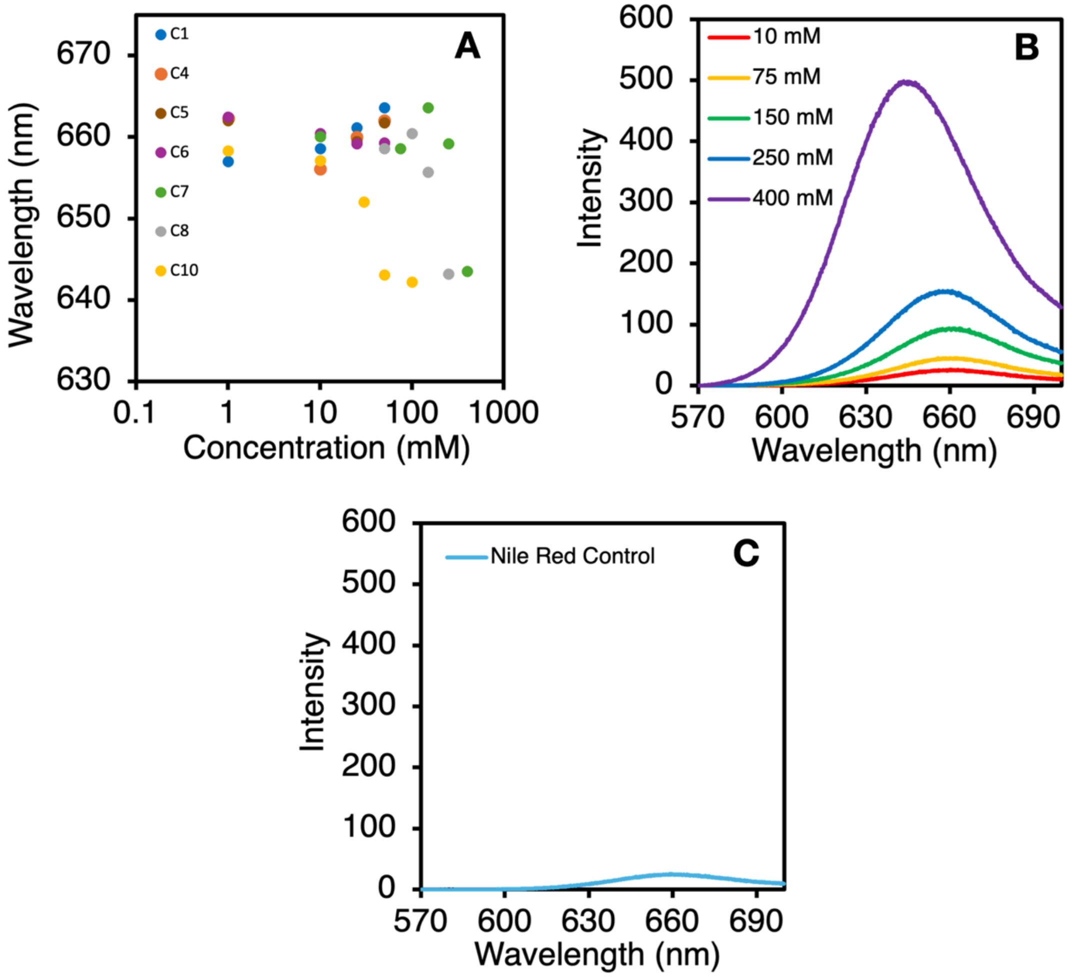

Figure S14. Alkyl sulfonate micelle studies using fluorescence spectroscopy. **A)** The wavelength of maximum emission of Nile Red as functions of sodium alkyl sulfonate concentration on a logarithmic scale. The alkyl sulfonates vary in carbon chain length (C = 1, 4, 5, 6, 7, 8, 9, 10); **B)** Representative fluorescence spectra of Nile Red in the presence of 10, 75, 150, 250, and 400 mM sodium 1-heptanesulfonate. A blue-shift in maximum emission wavelength and increase in emission intensity is observed as sulfonate concentration increases. **C)** Fluorescence spectra for a control sample with 25 µL of 0.05 mM Nile Red and 25 µL of 821 mM sodium chloride in Milli-Q water. The wavelength of maximum emission of Nile Red in the control is 658.8 nm. For all samples, the excitation wavelength was set to 550 nm and emission spectra were recorded between 570 nm and 700 nm at a scan rate of 13 nm/min. The ionic strength of each sample was adjusted to 8.21 mM with sodium chloride to match the ionic strength of the silver nanocrystal reactions.

Table S8. Sodium alkyl sulfonate experimental CMC ranges compared to literature values.

Chain Length	Experimental CMC Range (mM)	Literature CMC (mM)[7,8]
C1	> 50	Not Reported
C4	> 50	Not Reported
C5	> 50	990[8] – 1040[7]
C6	> 50	460[8] – 540[7]
C7	250 - 400	302[7]
C8	150 - 250	130[8] – 155[7]
C10	30 - 50	44[8]

In the presence of surfactant micelles in aqueous media, the maximum emission wavelength for Nile Red is expected to blue shift and the fluorescence intensity to increase.[4,9] The critical micelle concentration (CMC) is identified as the equivalence point in the region of the blue shift of the maximum emission wavelengths.[9] A blue-shift in maximum emission wavelength was observed for sodium 1-heptanesulfonate (CMC range of 250 – 400 mM), sodium 1-octanesulfonate (CMC range of 150 – 250 mM), and sodium 1-decanesulfonate (CMC range of 30 – 50 mM). The CMC ranges for these long-chain alkyl sulfonates exceed the sulfonate concentration used in silver nanocrystal synthesis (0.1 mM – 30 mM), suggesting the lack of micelle formation in reactions using these sulfonates.

Sodium 1-methanesulfonate, sodium 1-butanesulfonate, sodium 1-pentanesulfonate, and sodium 1-hexanesulfonate were each screened from concentrations of 1 mM to 50 mM for fluorescence studies. Sodium 1-methanesulfonate and sodium 1-butanesulfonate CMC values are not reported in the literature. If these short-chain sulfonates form micelles, the CMC would be orders of magnitude above the maximum concentration (30 mM) used for nanocrystal synthesis. Sodium 1-pentanesulfonate and sodium 1-hexanesulfonate have CMC literature values that far exceed the maximum sulfonate concentration in the silver nanocrystal reactions.[7,8] As expected, no blue-shift in maximum emission wavelength was observed, suggesting a lack of micelle formation when these sulfonates are used in the silver nanocrystal reactions.

Equilibration Method Study

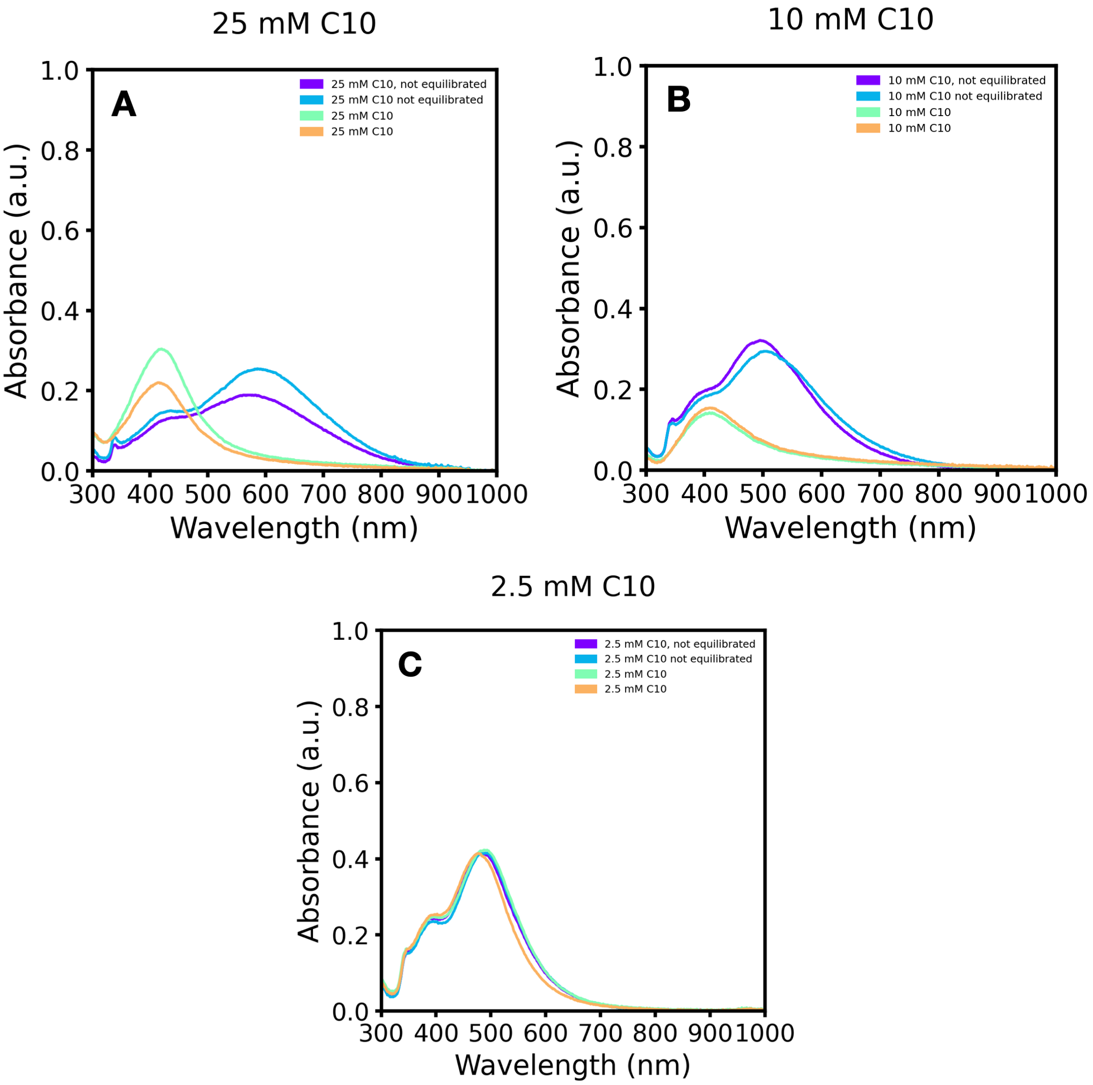

Figure S15. Absorbance spectra for silver nanocrystals synthesized with sodium 1-decanesulfonate that was prepared by various methods. **A)** Absorbance spectra for 25 mM decanesulfonate nanocrystals that used stock solutions of 100 mM decanesulfonate that were prepared by different equilibration methods (two duplicate reactions shown for each sulfonate preparation method). **B)** Absorbance spectra for 10 mM decanesulfonate nanocrystals that used stock solutions of 100 mM decanesulfonate that were prepared by different equilibration methods (two duplicate reactions shown for each sulfonate preparation method). **C)** Absorbance spectra for 2.5 mM decanesulfonate nanocrystals that used stock solutions of 100 mM decanesulfonate that were prepared by different equilibration methods (two duplicate reactions shown for each sulfonate preparation method).

A shift in λ_{max} and changes to the nanocrystal absorbance spectra were observed when decanesulfonate stock solutions were prepared by different methods and used at higher

concentrations in nanocrystal reactions. In reactions with 25 mM and 10 mM decanesulfonate, a blue-shift in the λ_{max} value is observed when the non-equilibrated decanesulfonate stock solution was used in the nanocrystal synthesis. In comparison, when a lower concentration of decanesulfonate, 2.5 mM, was introduced into reaction solutions, the spectra appear similar to each other, regardless of decanesulfonate preparation conditions. This result could be explained by the CMC of decanesulfonate, which was experimentally determined to be between 30 mM and 50 mM (Table S8). Because stock solutions were prepared at 100 mM concentrations, it is possible that the preparation conditions of the solution could affect micelle formation. Further, because 100 mM solutions were diluted to 10 mM for use in reactions with decanesulfonate concentrations at and below 3 mM, the dilution of the 100 mM decanesulfonate could disperse micelles in the 10 mM solutions for both methods of equilibration, resulting in more similar results for reactions with lower concentrations of decanesulfonate.

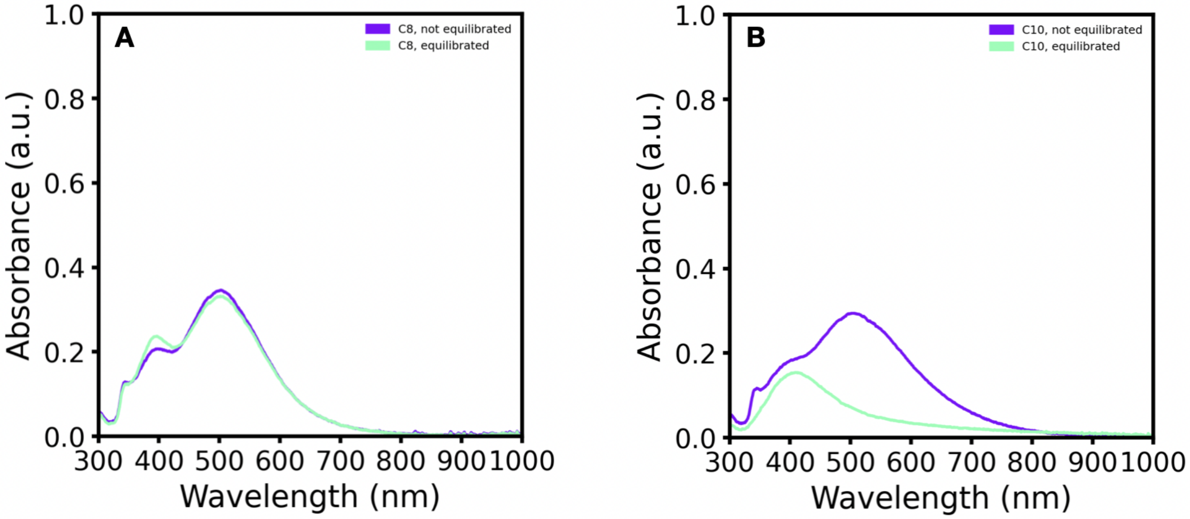

Figure S16. Absorbance spectra for silver nanocrystals synthesized with 10 mM sodium 1-decanesulfonate or 10 mM sodium 1-octanesulfonate using stock solutions prepared by various methods. **A)** Absorbance spectra for 10 mM octanesulfonate silver nanocrystals for which the stock solution of 100 mM octanesulfonate was prepared by different methods. **B)** Absorbance spectra for 10 mM decanesulfonate silver nanocrystals for which the stock solution of 100 mM decanesulfonate was prepared by different methods.

The effects of sulfonate solution equilibration on nanocrystal reactions were also investigated for sodium 1-octanesulfonate, the sulfonate with the next longest alkyl chain length, and thus the next lowest CMC after decanesulfonate. At 10 mM decanesulfonate reaction concentrations, a blue-shift in the λ_{max} of the nanocrystals is observed when the non-equilibrated sulfonate stock solution was used in the synthesis. At the same reaction concentration of 10 mM, no significant change in the absorbance spectra of the silver nanocrystals was observed when using octanesulfonate solutions prepared under either equilibration condition. This result can be explained by the higher CMC range for octanesulfonate (130 – 155 mM, Table S8) compared to decanesulfonate (30 – 50 mM, Table S8). Therefore, micelles would form in the 100 mM decanesulfonate stock solution

and could influence nanocrystal synthesis. 100 mM is below the CMC for octanesulfonate, so micelles would not form to the same extent, which could explain why the octanesulfonate equilibration method does not appear to influence nanocrystal absorbance.

Transmission Electron Microscopy

Sodium Nitrate Nanocrystals

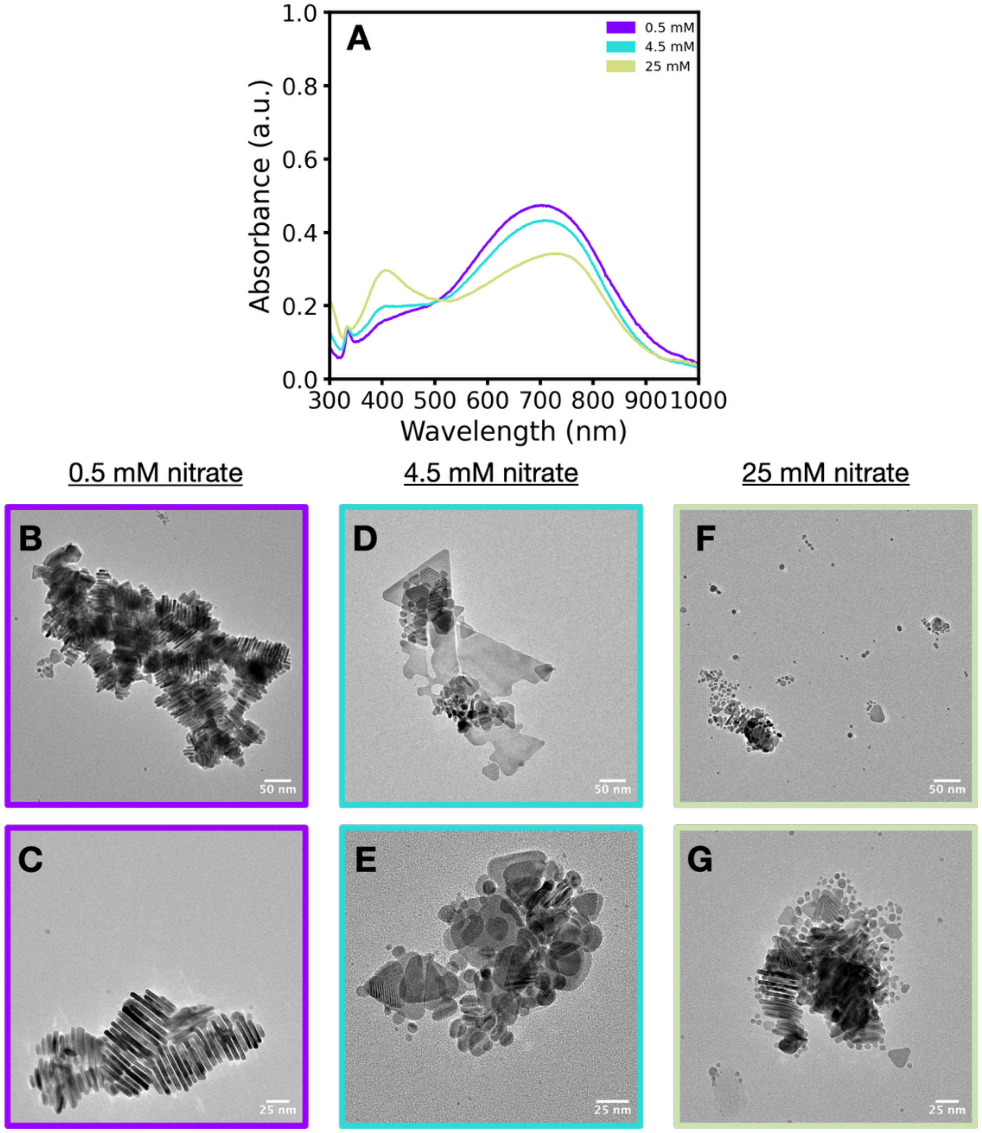

Figure S17. Absorbance spectra and transmission electron micrographs of silver nanocrystals with varying sodium nitrate concentrations. **A)** Absorbance spectra of silver nanocrystals synthesized with 0.5 mM, 4.5 mM, and 25 mM sodium nitrate; **B-G)** Transmission electron micrographs of silver nanocrystals synthesized with 0.5 mM (**B-C**, left column, purple), 4.5 mM (**D-E**, middle column, teal), and 25 mM (**F-G**, right column, green) sodium nitrate with increasing magnification from top to bottom.

Sodium 1-butanesulfonate Nanocrystals

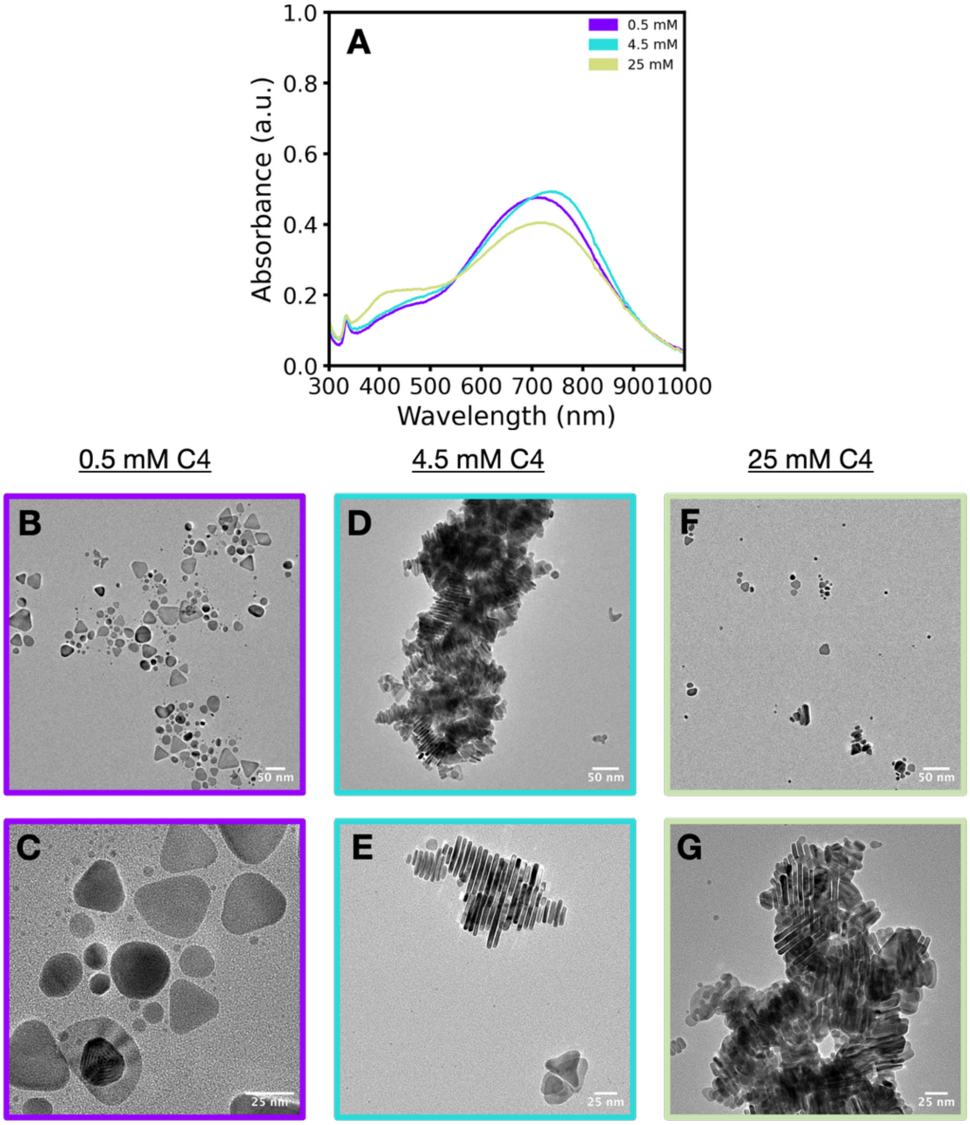

Figure S18. Absorbance spectra and transmission electron micrographs of silver nanocrystals with varying sodium 1-butanesulfonate concentrations. **A)** Absorbance spectra of silver nanocrystals synthesized with 0.5 mM, 4.5 mM, and 25 mM sodium 1-butanesulfonate; **B-G)** Transmission electron micrographs of silver nanocrystals synthesized with 0.5 mM (**B-C**, left column, purple), 4.5 mM (**D-E**, middle column, teal), and 25 mM (**F-G**, right column, green) sodium 1-butanesulfonate with increasing magnification from top to bottom.

Sodium 1-hexanesulfonate Nanocrystals

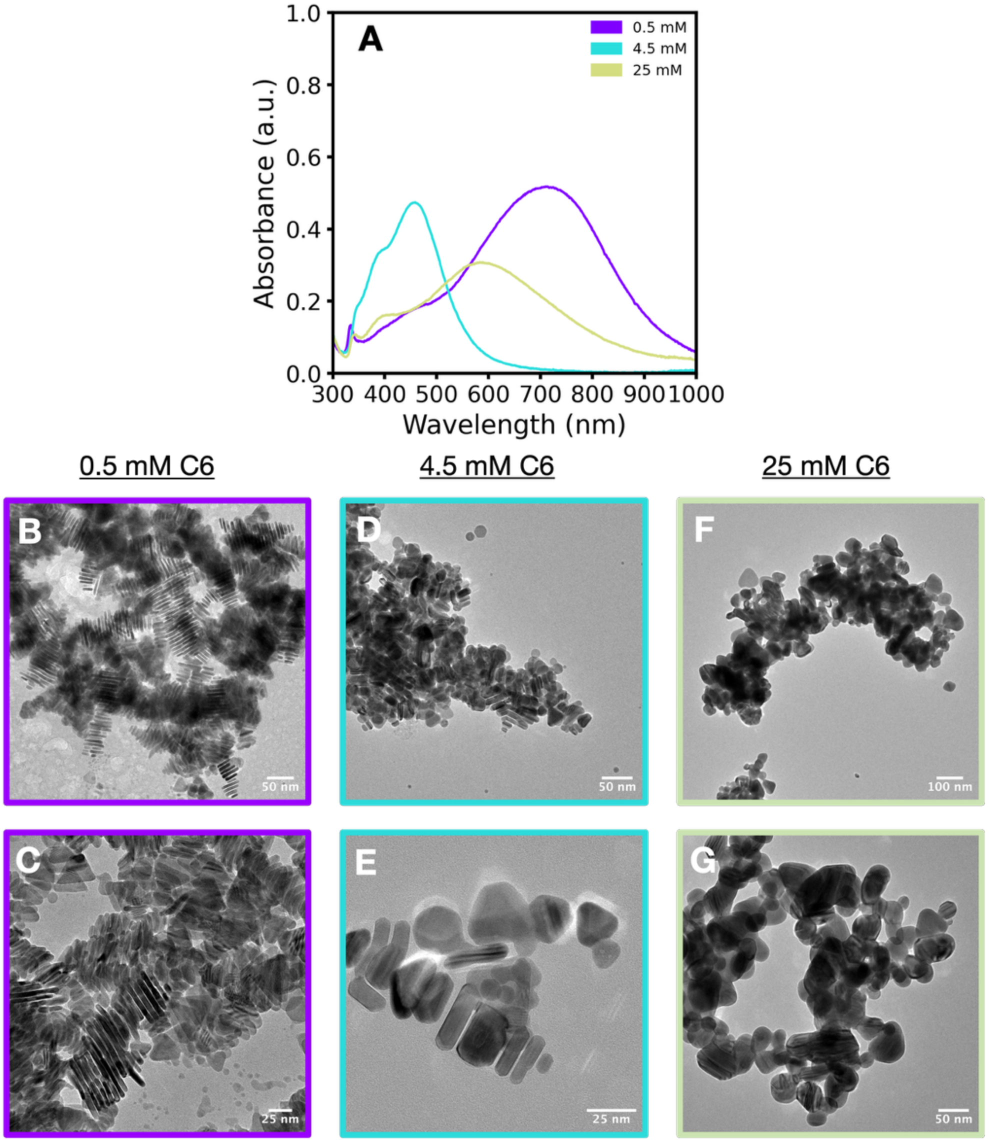

Figure S19. Absorbance spectra and transmission electron micrographs of silver nanocrystals with varying sodium 1-hexanesulfonate concentrations. **A)** Absorbance spectra of silver nanocrystals synthesized with 0.5 mM, 4.5 mM, and 25 mM sodium 1-hexanesulfonate; **B-G)** Transmission electron micrographs of silver nanocrystals synthesized with 0.5 mM (**B-C**, left column, purple), 4.5 mM (**D-E**, middle column, teal), and 25 mM (**F-G**, right column, green) sodium 1-hexanesulfonate with increasing magnification from top to bottom.

Sodium 1-octanesulfonate Nanocrystals

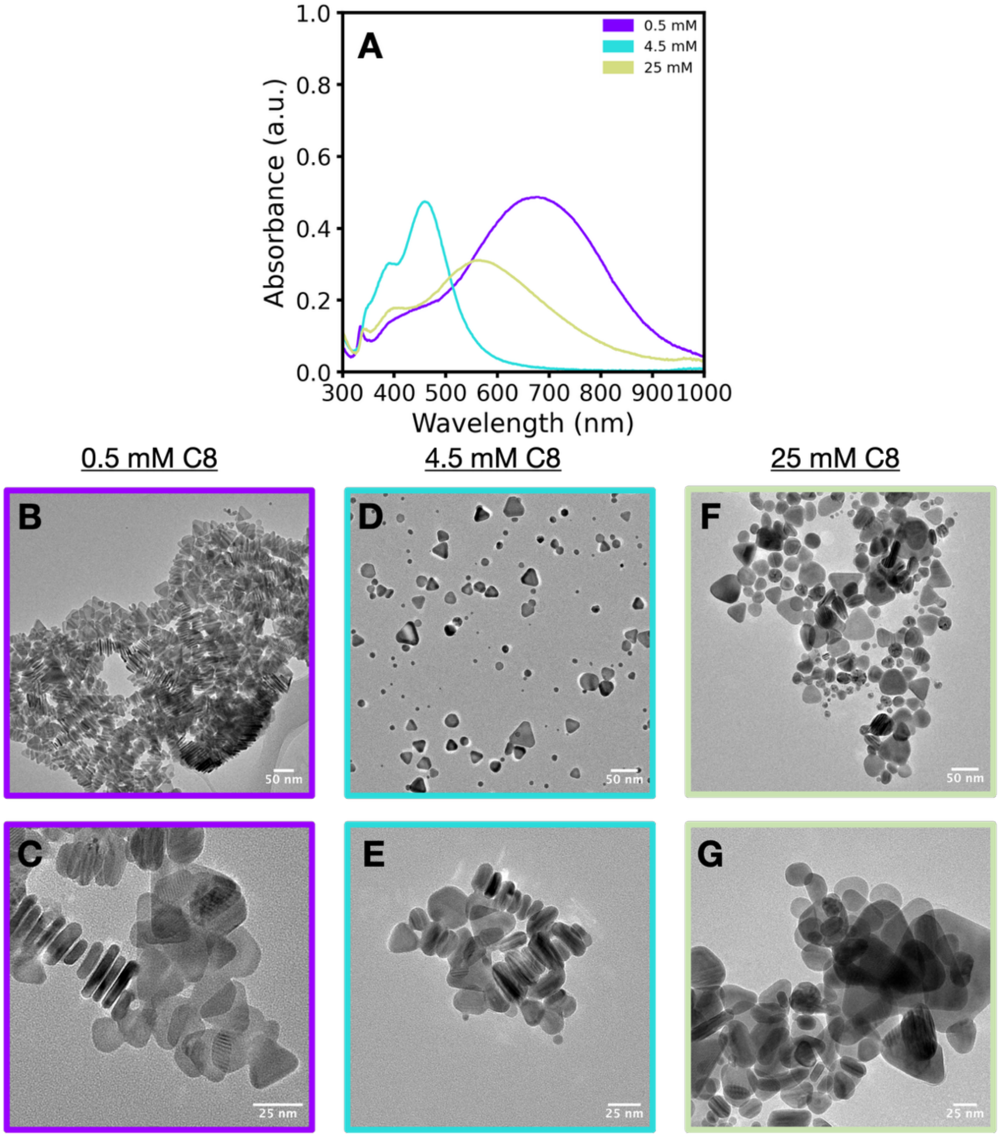

Figure S20. Absorbance spectra and transmission electron micrographs of silver nanocrystals with varying sodium 1-octanesulfonate concentrations. **A)** Absorbance spectra of silver nanocrystals synthesized with 0.5 mM, 4.5 mM, and 25 mM sodium 1-octanesulfonate; **B-G)** Transmission electron micrographs of silver nanocrystals synthesized with 0.5 mM (**B-C**, left column, purple), 4.5 mM (**D-E**, middle column, teal), and 25 mM (**F-G**, right column, green) sodium 1-octanesulfonate with increasing magnification from top to bottom.

Post-Synthesis Ligand Addition: Sodium 1-octanesulfonate

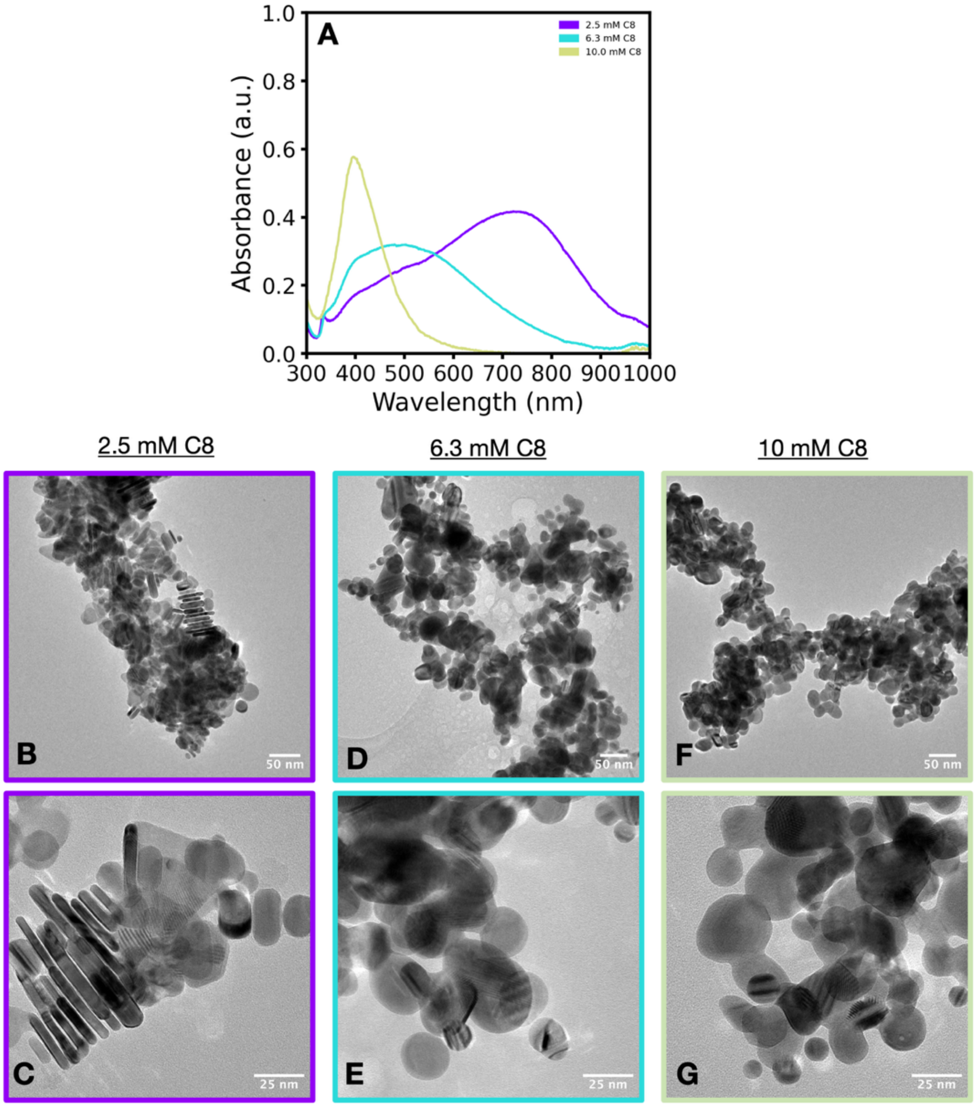

Figure S21. Absorbance spectra and transmission electron micrographs of 10 mM sodium 1-butanesulfonate silver nanocrystals to which varying concentrations of sodium 1-octanesulfonate was added 21 min post-synthesis. **A)** Absorbance spectra of nanocrystals initially synthesized with 10 mM sodium 1-butanesulfonate to which sodium 1-octanesulfonate was added post-synthesis at reaction concentrations of 2.5 mM (purple), 6.3 mM (teal), and 10.0 mM (green); **B-G)** Transmission electron micrographs of silver nanocrystals with post-synthesis addition of 2.5 mM (**B-C**, left column, purple), 6.3 mM (**D-E**, middle column, teal), and 10 mM (**F-G**, right column, green) sodium 1-octanesulfonate with increasing magnification from top to bottom. A bubble was present in the reaction with 10.0 mM sodium 1-octanesulfonate that caused scattering and raised the baseline. The spectrum baseline was corrected by subtracting 0.540 a.u. from the absorbance values between 300 nm and 1000 nm.

Nanocrystal Dissolution

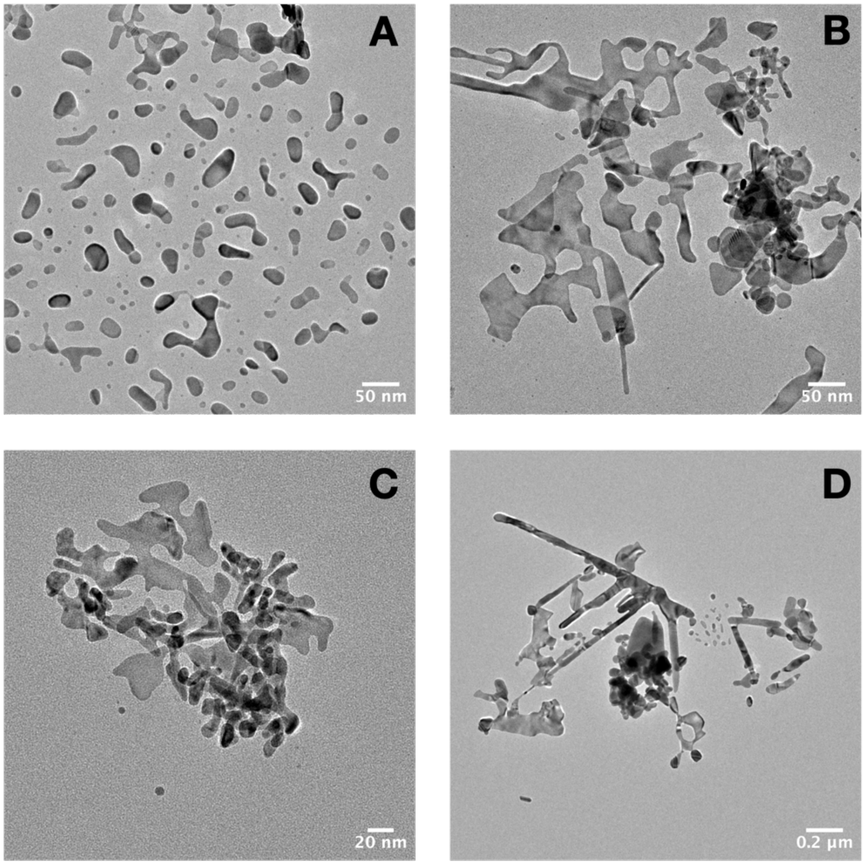

Figure S22. Transmission electron micrographs showing possible silver nanocrystal decomposition, potentially from extended time of drying on the grid or from exposure to the electron beam. **A)** 0.5 mM sodium 1-octanesulfonate nanocrystals; **B)** 0.5 mM sodium nitrate nanocrystals; **C)** 4.5 mM sodium 1-butanesulfonate nanocrystals; **D)** 25 mM sodium 1-hexanesulfonate nanocrystals.

TEM Measurements: Sodium 1-butanesulfonate Nanocrystals

Table S9. Measurements of nanocrystal thickness from image analysis of transmission electron micrographs of 4.5 mM sodium 1-butanesulfonate nanocrystals.

Measurement Number	Nanoparticle Thickness (nm)
1	4.61
2	4.69
3	5.57
4	5.33
5	6.50
6	5.20
7	5.69
8	4.77
9	4.33
10	4.65
11	4.73
12	5.36
13	4.97
14	6.09
15	5.33
16	5.33
17	5.61
18	6.39
19	6.74
20	6.85
21	5.39
22	5.93
23	5.41
24	5.77
25	5.88
26	5.68
27	4.96
28	4.97
29	4.49
30	4.82
31	5.34
32	4.90
33	5.97
34	4.35
35	6.25
36	3.68
37	3.81
38	4.31
39	4.23
40	4.85
41	5.13
42	5.61
43	4.38
44	4.31
45	5.84
46	5.10
47	5.07
48	5.60
49	5.66
50	5.89
51	5.31
52	5.91
53	4.82
54	4.59
**Average**	5.24
**Standard Deviation**	0.71

TEM Measurements: Sodium 1-octanesulfonate Nanocrystals

Table S10. Measurements of nanocrystal thickness from image analysis of transmission electron micrographs of 4.5 mM sodium 1-octanesulfonate nanocrystals.

Measurement Number	Nanoparticle Thickness (nm)
1	9.01
2	11.50
3	8.68
4	7.84
5	7.84
6	11.31
7	9.01
8	10.31
9	5.87
10	13.09
11	9.55
12	10.46
13	9.38
14	7.07
15	6.78
16	14.18
**Average**	9.49
**Standard Deviation**	2.26

NMR Studies Discussion

The Murphy group first reported methods to use NMR to study larger (>6 nm diameter) gold nanocrystals in 2019.[10] While the alkyl sulfonate silver nanocrystals described herein are quite different than the nanocrystals studied by the Murphy group, we nonetheless made significant efforts to study them by NMR. One of the key insights of the Murphy group was that the low concentration of most metallic nanocrystal systems was one of the primary limitations to their study with NMR, which has a relatively high detection limit. In response, the Murphy group concentrates their gold nanocrystals prior to measurement. Our efforts to concentrate the alkyl sulfonate silver nanocrystals proved challenging and, in many cases, resulted in poor stability and subsequent precipitation. In cases where we were able to concentrate them, the NMR measurements were inconclusive. The methods described by the Murphy group were only demonstrated on monodisperse nanocrystals up to 25 nm in diameter, which itself is an impressive feat, whereas the alkyl sulfonate silver nanocrystals are highly polydisperse and range in size up to hundreds of nanometers (see S17-S20). The large size of these nanocrystals will slow their rotation in the NMR, resulting in extreme homogeneous broadening.[11] Additionally, the Murphy group has primarily used their method on nanocrystals with alkyl thiol ligands that are among the strongest binding ligands to gold and therefore have minimal dynamic exchange on the surface,[12–14] in contrast to what we expect is a highly dynamic surface for the alkyl sulfonate nanocrystals. We believe that the size, polydispersity, and dynamic equilibrium of the alkyl sulfonates on the surface of these silver nanocrystals preclude the use of standard NMR techniques to study these nanocrystals, and we have not been able to conclusively measure anything except free alkyl sulfonate and citrate in solution.

Sulfonate Combination Studies: % Sulfonate vs λ_{max}

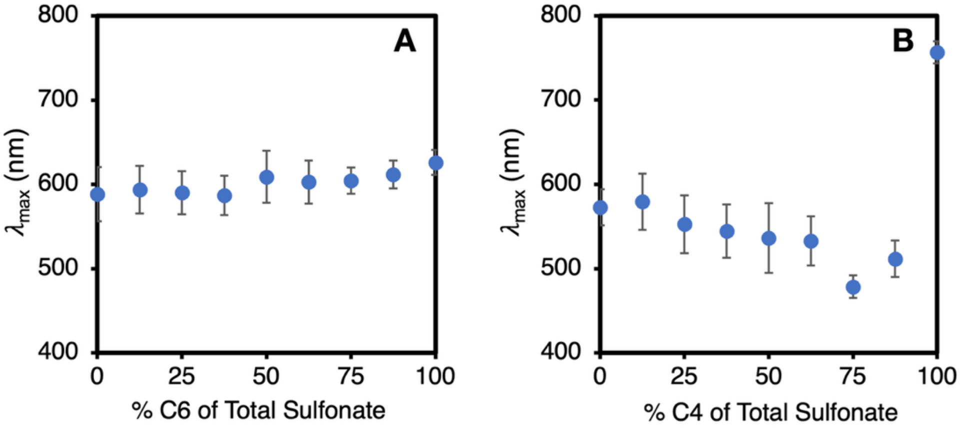

Figure S23. λ_{max} values for nanocrystals synthesized with two sulfonates of different chain lengths at various ratios with a constant total concentration of 20 mM. **A)** λ_{max} of nanocrystals with various ratios of sodium 1-hexanesulfonate and sodium 1-octanesulfonate at a constant total sulfonate concentration of 20 mM. Data is plotted as the percent of hexanesulfonate in the reaction; **B)** λ_{max} of reactions with various ratios of sodium 1-butanesulfonate and sodium 1-octanesulfonate at a constant total sulfonate concentration of 20 mM. Data is plotted as the percent of butanesulfonate in the reaction. The data represents an average from 3-4 reactions and error bars represent one standard deviation.

Sulfonate Combination Studies: Absorbance Spectra

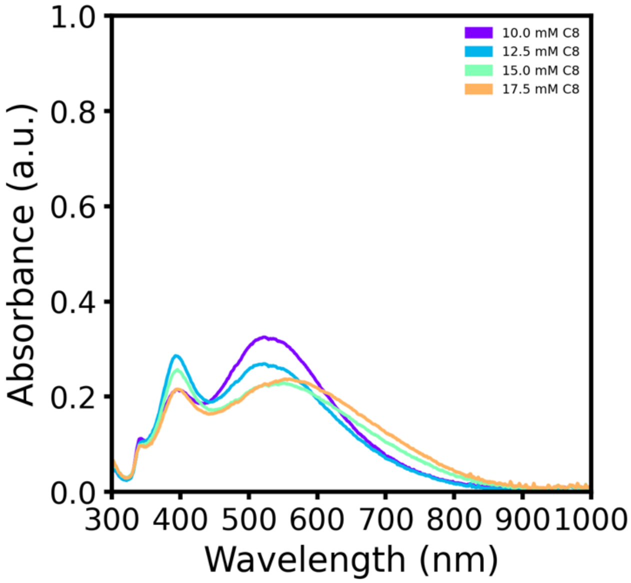

Figure S24. Representative absorbance spectra of silver nanocrystals synthesized with varying ratios of sodium 1-butanesulfonate (C4) to sodium 1-octanesulfonate (C8) concentration. In some cases, the peak with λ_{max} ~400 nm displays a stronger absorbance than the peak at ~500 nm characteristic of nanocrystals synthesized with higher concentrations of long-chain sulfonates. The legend displays the concentration of C8 in a reaction. The spectra represent the scan recorded closest to 45 minutes after the addition of sodium borohydride to a reaction, with a range of 30 to 80 minutes post-borohydride addition.

Kinetics of Post-Synthesis Ligand Addition

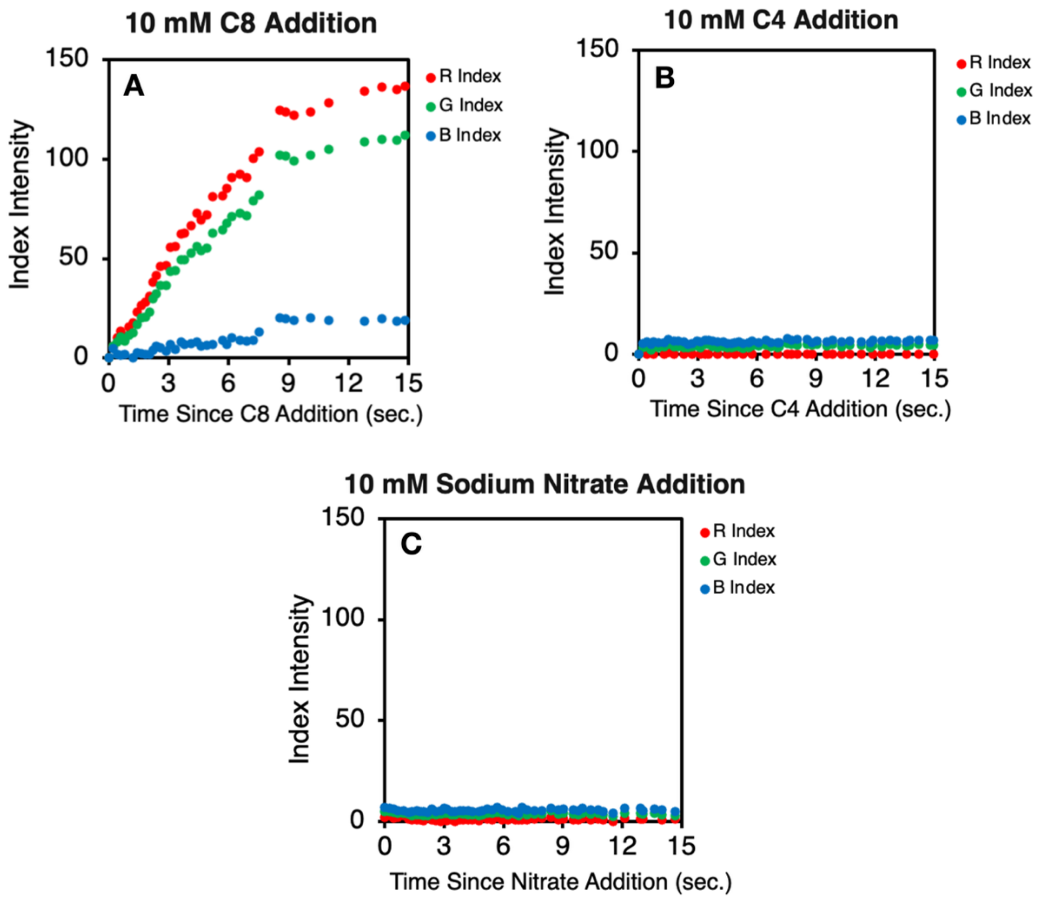

Figure S25. Kinetics from video analysis of post-synthesis ligand addition studies with 10 mM sodium 1-butanesulfonate nanocrystals to which 10 mM sodium 1-octanesulfonate, 10 mM sodium 1-butanesulfonate, and 10 mM sodium nitrate were added. **A)** RGB Index values of 10 mM butanesulfonate nanocrystals with post-synthesis addition of 10 mM octanesulfonate; **B)** RGB Index values of 10 mM butanesulfonate nanocrystals with post-synthesis addition of 10 mM butanesulfonate; **C)** RGB Index values of 10 mM butanesulfonate nanocrystals with post-synthesis addition of 10 mM sodium nitrate. In each plot, the baseline was subtracted and in **B** and **C**, 5 nm was added to each G index intensity value and 7 nm was added to each B index intensity value to make visible the overlapping data in the plot.

Time Variation of Post-Synthesis Sulfonate Addition

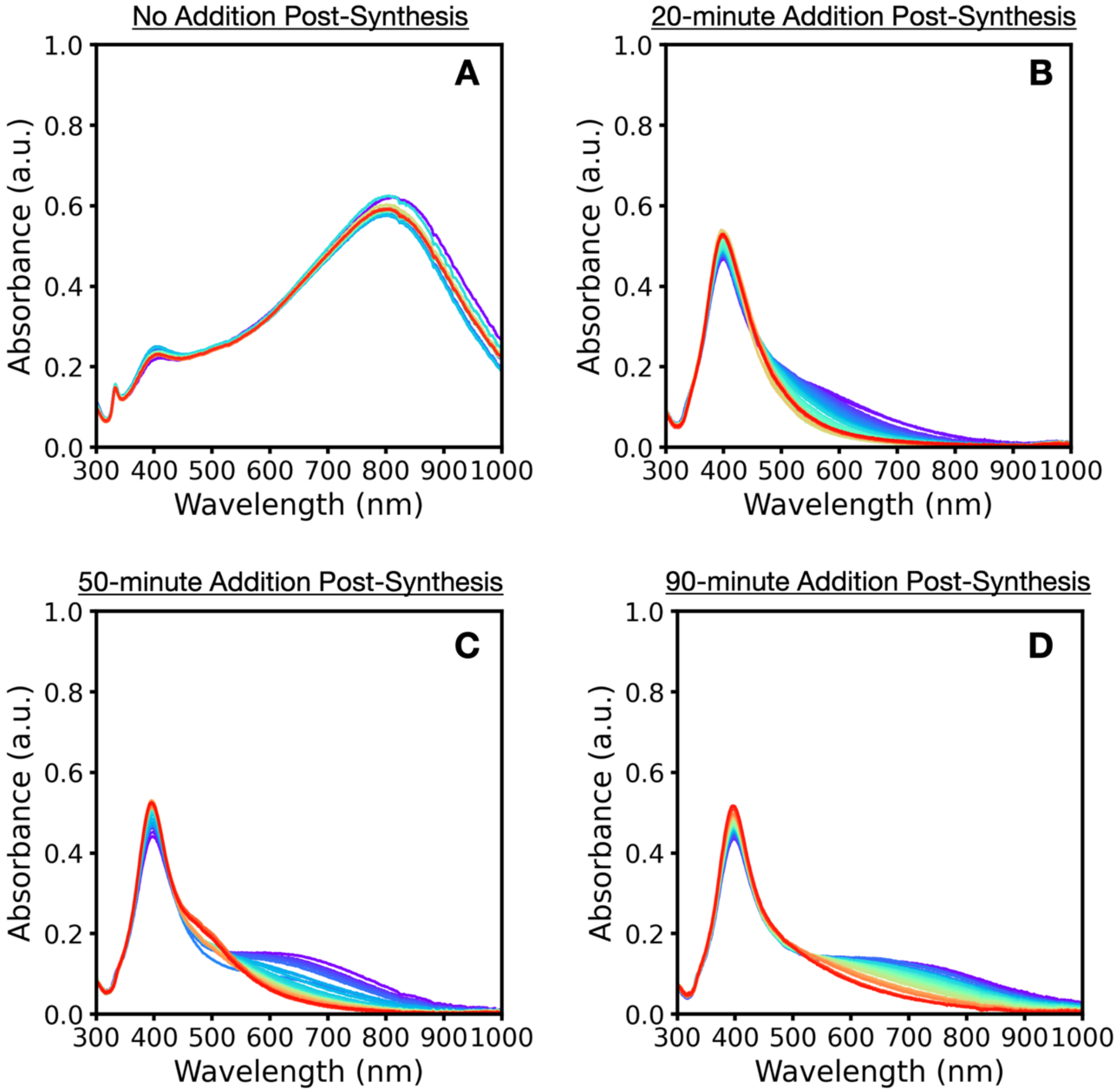

Figure S26. Absorbance spectra for 10 mM sodium 1-butanesulfonate (C4) silver nanocrystals to which 10 mM sodium 1-octanesulfonate (C8) was added at various times post-nanocrystal synthesis. **A)** 10 mM C4 nanocrystals to which there was no later sulfonate addition. The nanocrystals were allowed to grow for 20 minutes and then absorbance spectra recorded over a period of about 70 minutes. **B)** 10 mM C4 nanocrystals to which 10 mM C8 was added about 20 minutes post-synthesis. Spectra were then recorded over a period of approximately 70 minutes. **C)** 10 mM C4 nanocrystals to which 10 mM C8 was added approximately 50 minutes post-synthesis. Spectra were then recorded over a period of approximately 43 minutes. **D)** 10 mM C4 nanocrystals to which 10 mM C8 was added about 90 minutes post-synthesis. Spectra were then recorded over a period of approximately 8 minutes.

Post-Synthesis Addition of Sodium Nitrate

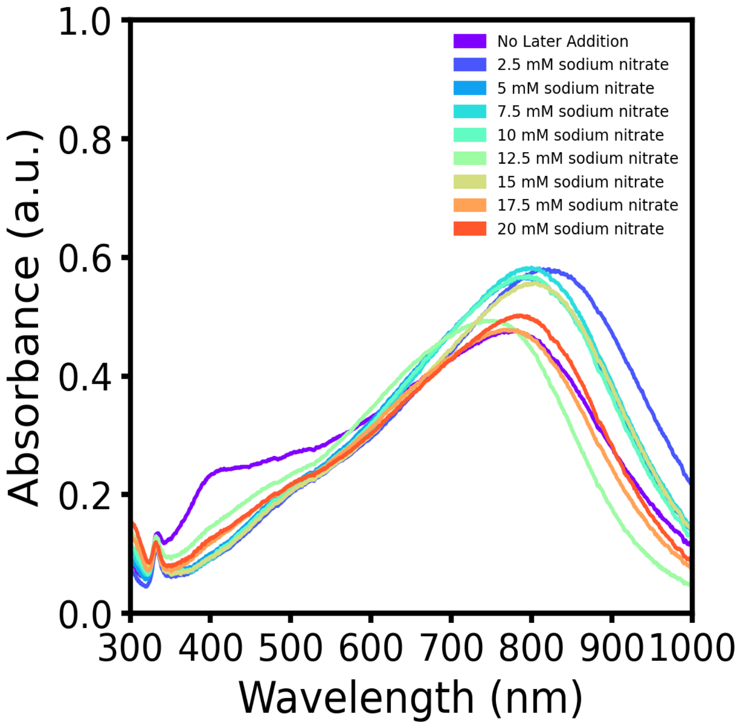

Figure S27. Absorbance spectra of silver nanocrystals initially synthesized with 10 mM sodium 1-butanesulfonate to which varying ~~reaction~~ concentrations of sodium nitrate (2.5 to 20 mM in reaction) were introduced post-synthesis. Spectra were recorded between 30 and 34 minutes after the addition of sodium nitrate to the reaction wells. A control reaction (purple) with no addition of sodium nitrate post-synthesis is included and was scanned about 68 minutes after borohydride addition.

As sodium nitrate concentration was increased, maximum absorbance intensity decreased but the λ_{max} did not dramatically blue-shift as observed when sodium 1-octanesulfonate was added post-synthesis. This suggests that ionic strength changes cannot explain λ_{max} shifts resulting from the addition of a long-chain additive.

Post-Synthesis Sulfonate Addition to Purified Sulfonate Nanocrystals

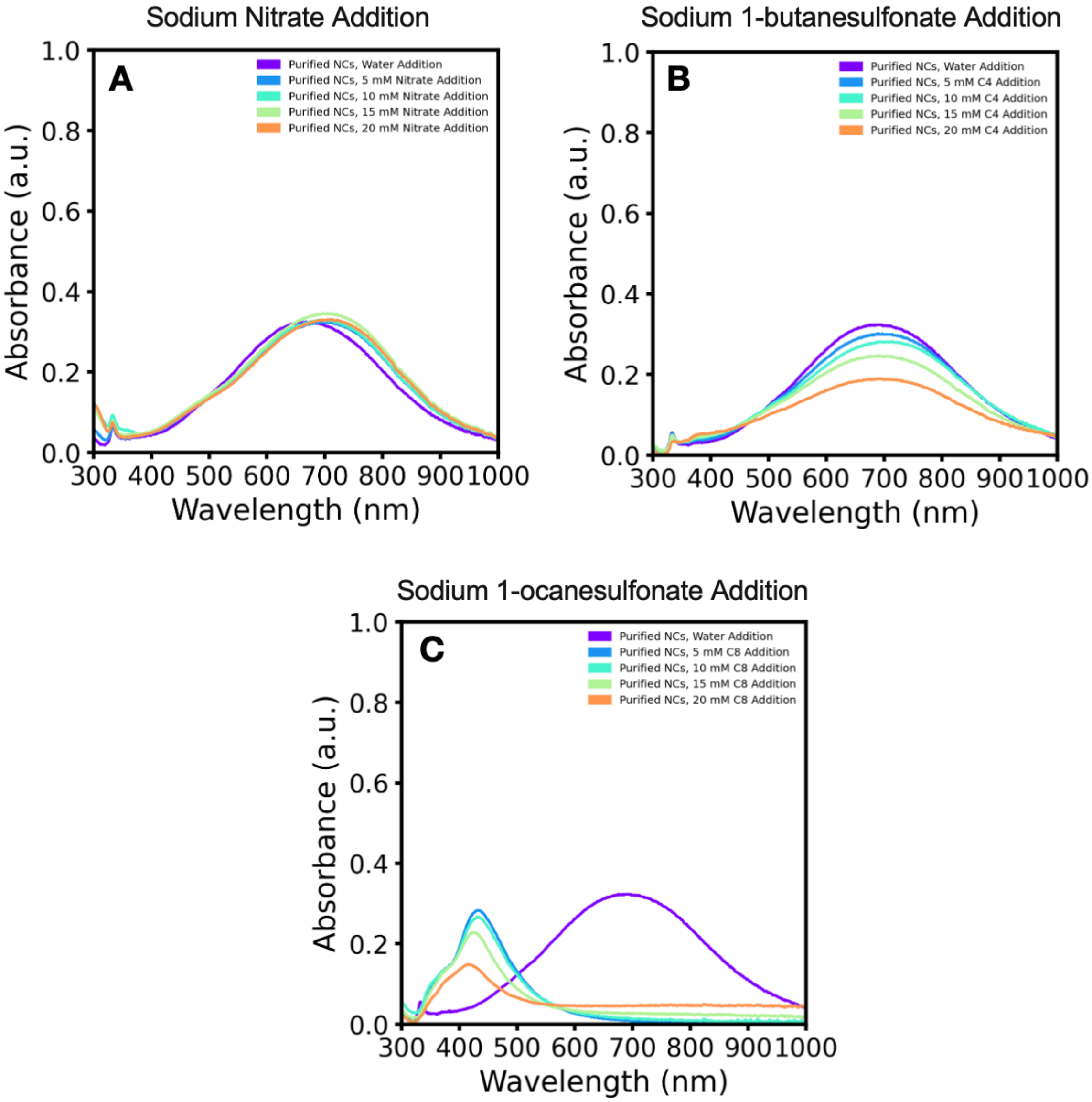

Figure S28. Absorbance spectra of purified 10 mM butanesulfonate nanocrystals to which varying concentrations of sodium nitrate or sulfonate were added post-synthesis. **A)** Absorbance spectra of purified 10 mM butanesulfonate nanocrystals with post-synthesis additions of water, 5 mM, 10 mM, 15 mM, or 20 mM sodium nitrate. **B)** Absorbance spectra of purified 10 mM butanesulfonate nanocrystals with post-synthesis additions of water, 5 mM, 10 mM, 15 mM, or 20 mM sodium 1-butanesulfonate (C4). **C)** Absorbance spectra of purified 10 mM butanesulfonate nanocrystals with post-synthesis additions of water, 5 mM, 10 mM, 15 mM, or 20 mM sodium 1-octanesulfonate (C8).

TEM for Post-Synthesis Sulfonate Addition to Bromide Nanocrystals

Citrate Nanocrystals

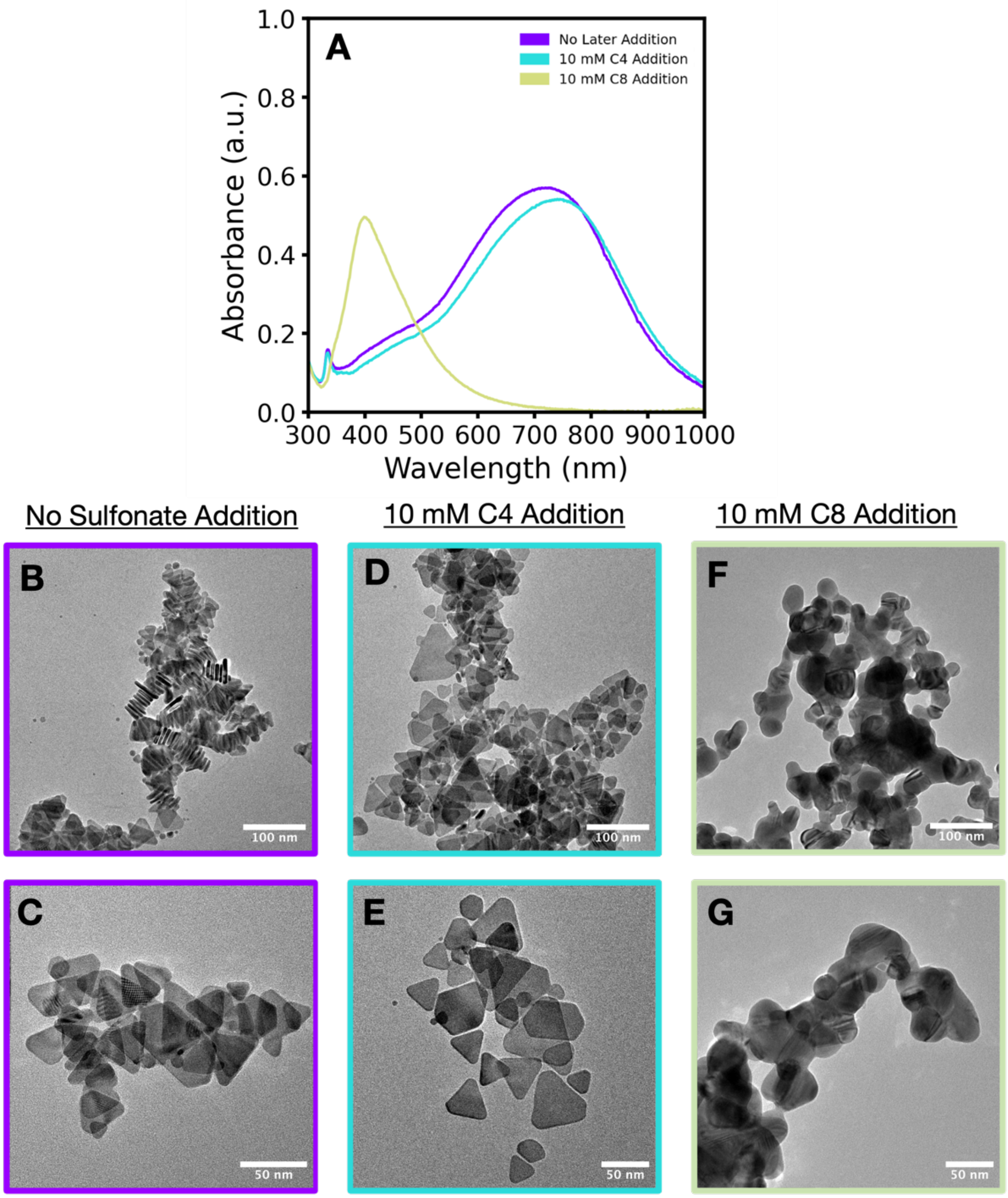

Figure S29. Absorbance spectra and transmission electron micrographs of citrate-capped silver nanocrystals with post-synthesis addition of water, sodium 1-butanesulfonate, or sodium 1-octanesulfonate. **A)** Absorbance spectra of citrate nanocrystals with post-synthesis addition of water (no sulfonate), 10 mM sodium 1-butanesulfonate, or 10 mM sodium 1-octanesulfonate; **B-G)** Transmission electron micrographs of citrate nanocrystals with post-synthesis addition of water (**B-C**, left column, purple), 10 mM sodium 1-butanesulfonate (**D-E**, middle column, teal), or 10 mM sodium 1-octanesulfonate (**F-G**, right column, green) with increasing magnification from top to bottom.

Citrate and Low Concentration Bromide (0.5 μM) Nanocrystals

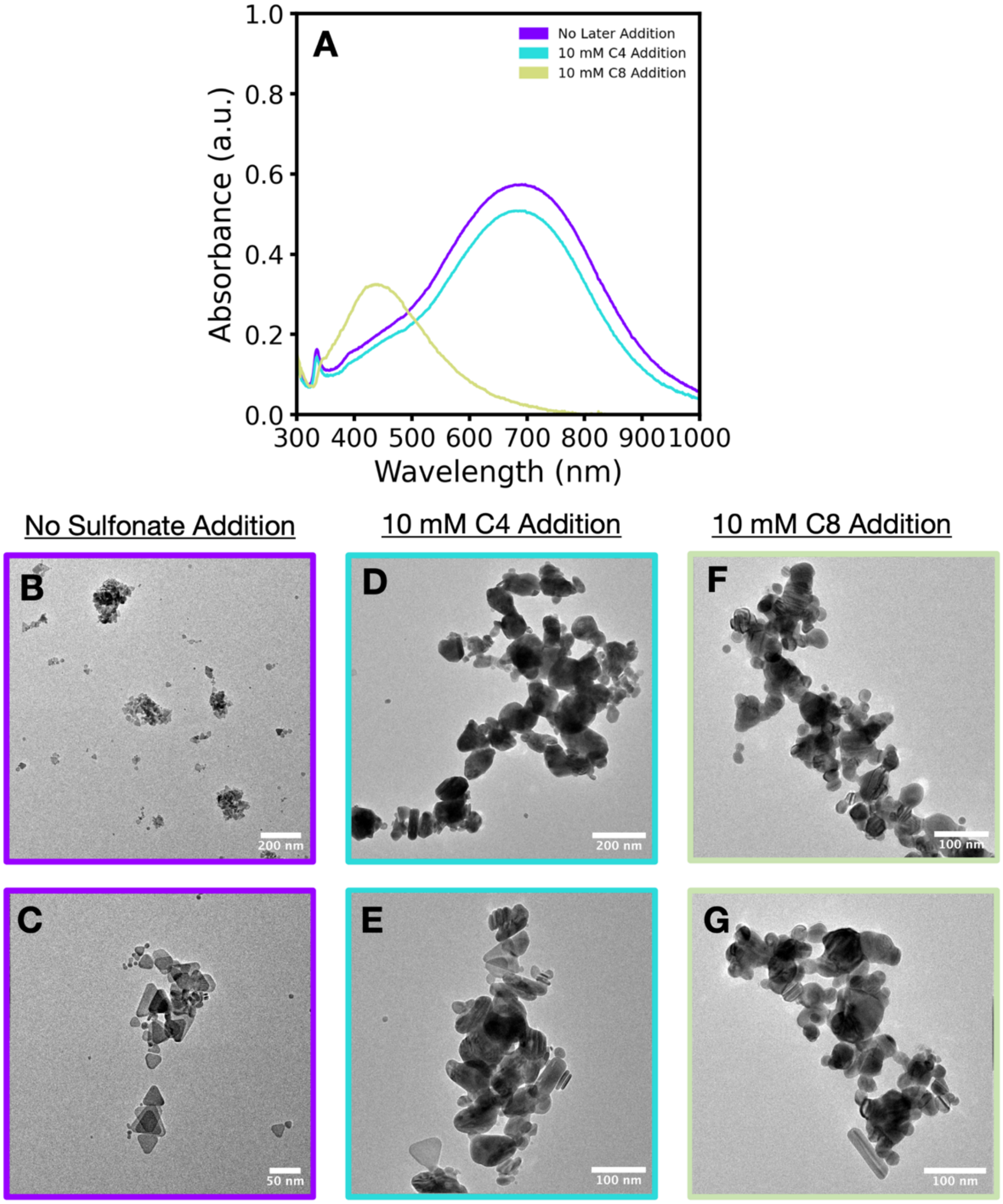

Figure S30. Absorbance spectra and transmission electron micrographs of silver nanocrystals synthesized with citrate and 0.5 μM potassium bromide to which water, sodium 1-butanesulfonate, or sodium 1-octanesulfonate was added post-synthesis. **A)** Absorbance spectra of citrate and 0.5 μM bromide nanocrystals with later addition of water (no sulfonate), 10 mM sodium 1-butanesulfonate, or 10 mM sodium 1-octanesulfonate; **B-G)** Transmission electron micrographs of 0.5 μM bromide nanocrystals with post-synthesis addition of water (**B-C**, left column, purple), 10 mM sodium 1-butanesulfonate (**D-E**, middle column, teal), or 10 mM sodium 1-octanesulfonate (**F-G**, right column, green) with increasing magnification from top to bottom. A bubble was

present in the reaction with 10.0 mM sodium 1-octanesulfonate that caused scattering and raised the baseline. The spectrum baseline was corrected by subtracting 0.07 a.u. from the absorbance values between 300 nm and 1000 nm.

Citrate and High Concentration Bromide (1.5 µM) *Nanocrystals*

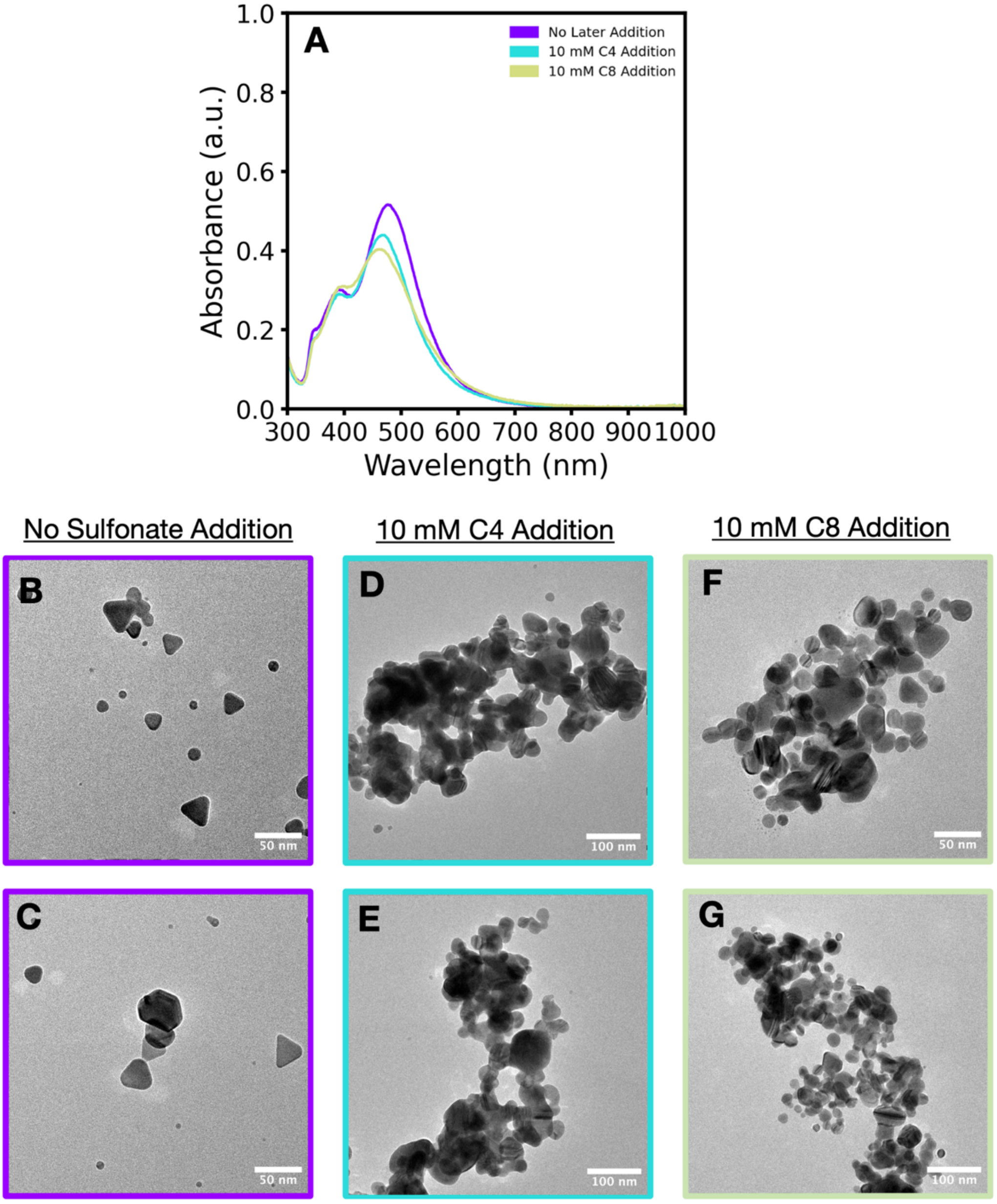

Figure S31. Absorbance spectra and transmission electron micrographs of silver nanocrystals synthesized with citrate and 1.5 µM potassium bromide to which water, sodium 1-butanesulfonate, or sodium 1-octanesulfonate was added post-synthesis. **A)** Absorbance spectra of citrate and 1.5 µM bromide nanocrystals with later addition of water (no sulfonate), 10 mM sodium 1-butanesulfonate, or 10 mM sodium 1-octanesulfonate; **B-G)** Transmission electron micrographs of citrate and 1.5 µM bromide nanocrystals with later addition of water (**B-C**, left column, purple), 10 mM sodium 1-butanesulfonate (**D-E**, middle column, teal), or 10 mM sodium 1-octanesulfonate (**F-G**, right column, green) with increasing magnification from top to bottom.

Post-Synthesis Sulfonate Addition to Nanocubes

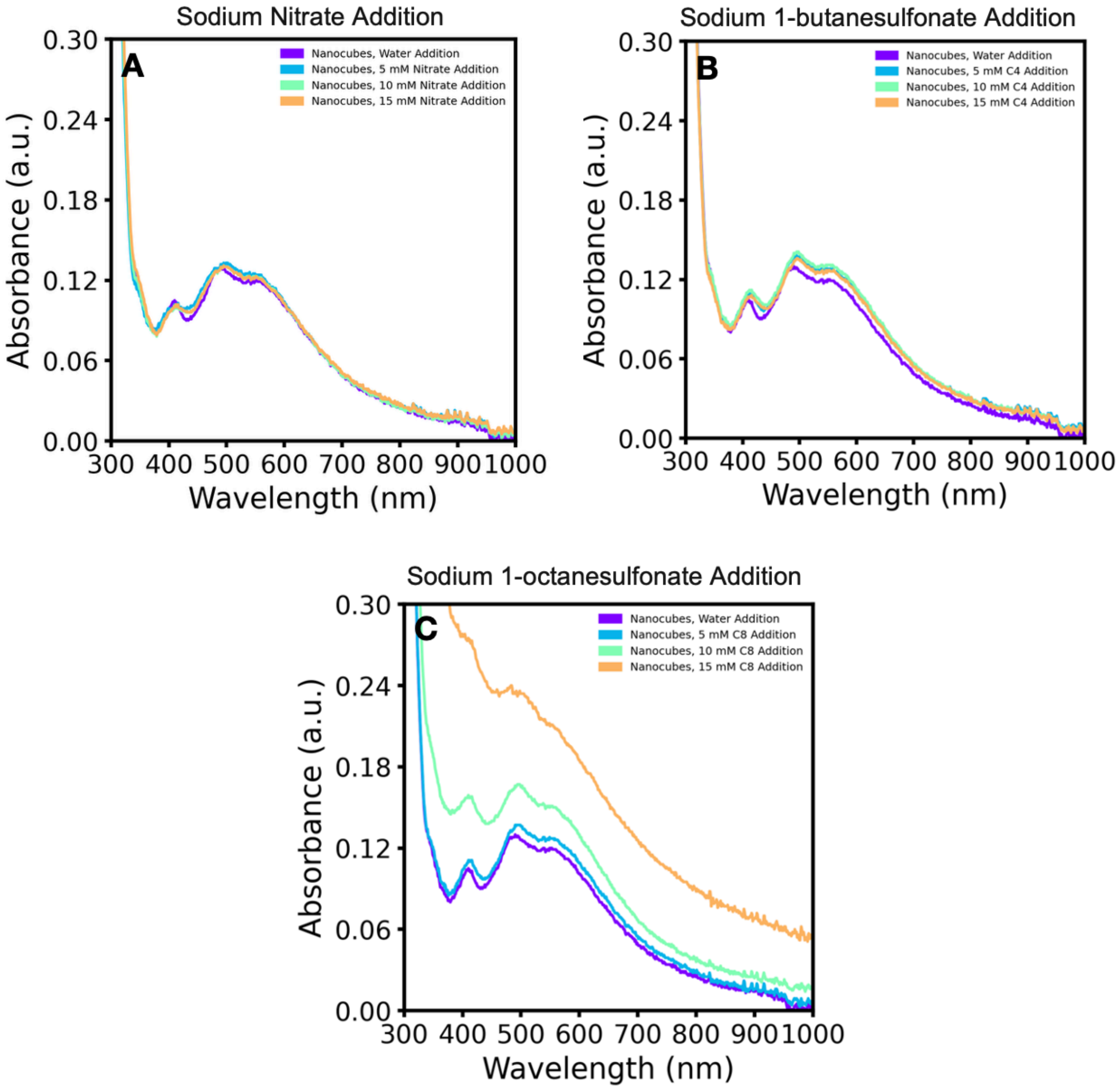

Figure S32. Absorbance spectra of unpurified silver nanocubes synthesized following the Zhou et al. procedure[3] to which varying concentrations of sodium nitrate or sulfonate were added post-synthesis. **A)** Absorbance spectra of unpurified nanocubes with post-synthesis additions of water, 5 mM, 10 mM, or 15 mM sodium nitrate. **B)** Absorbance spectra of unpurified nanocubes with post-synthesis additions of water, 5 mM, 10 mM, or 15 mM sodium 1-butanesulfonate (C4). **C)** Absorbance spectra of unpurified nanocubes with post-synthesis additions of water, 5 mM, 10 mM, or 15 mM sodium 1-octanesulfonate (C8).

Post-Synthesis Sulfonate Addition to Purified Nanocubes

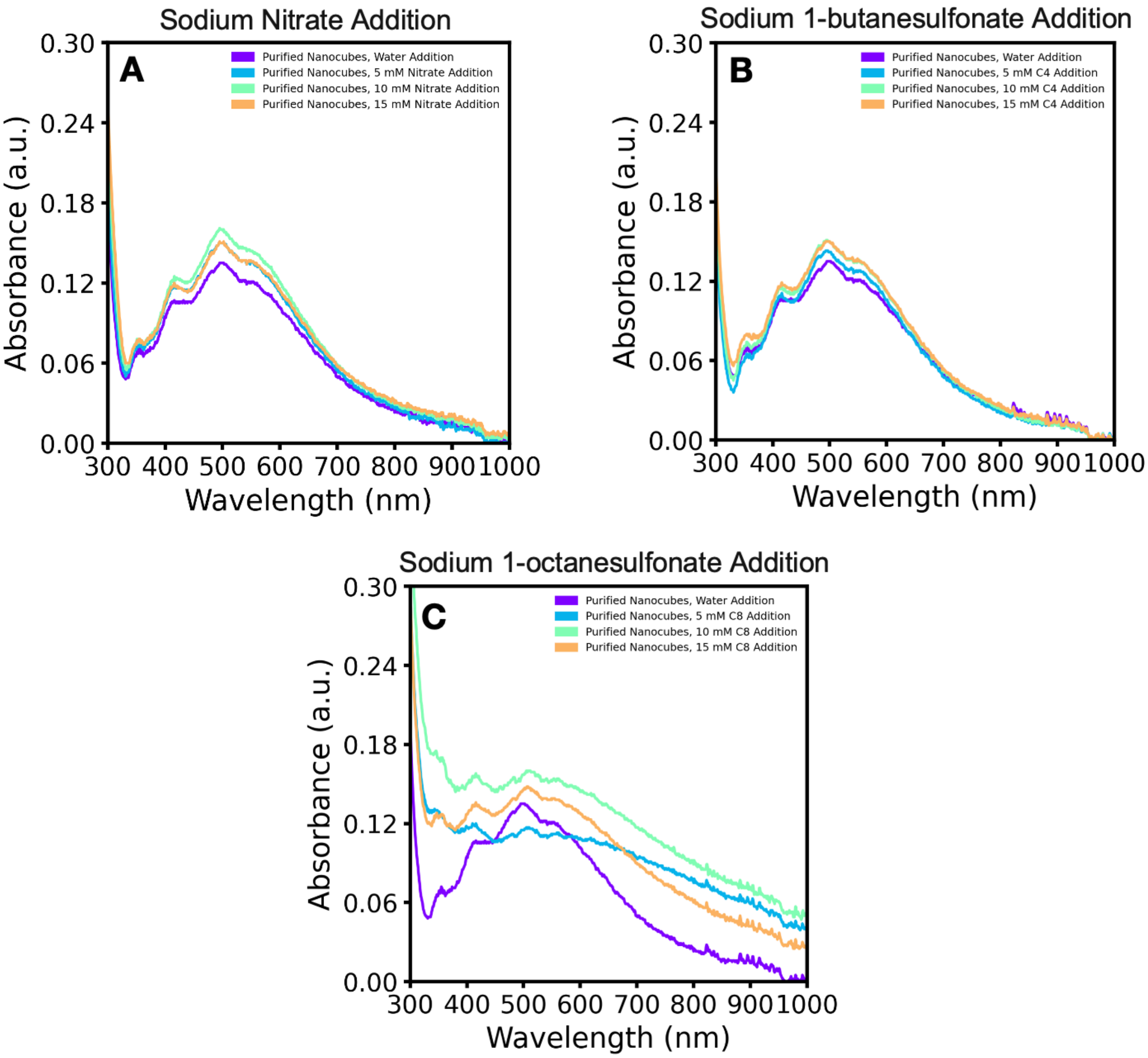

Figure S33. Absorbance spectra of purified silver nanocubes synthesized following the Zhou et al. procedure[3] to which varying concentrations of sodium nitrate or sulfonate were added post-synthesis. **A)** Absorbance spectra of purified nanocubes with post-synthesis additions of water, 5 mM, 10 mM, or 15 mM sodium nitrate. **B)** Absorbance spectra of purified nanocubes with post-synthesis additions of water, 5 mM, 10 mM, or 15 mM sodium 1-butanesulfonate (C4). **C)** Absorbance spectra of purified nanocubes with post-synthesis additions of water, 5 mM, 10 mM, or 15 mM sodium 1-octanesulfonate (C8).

TEM of Purified Nanocubes with Post-Synthesis Sulfonate Addition

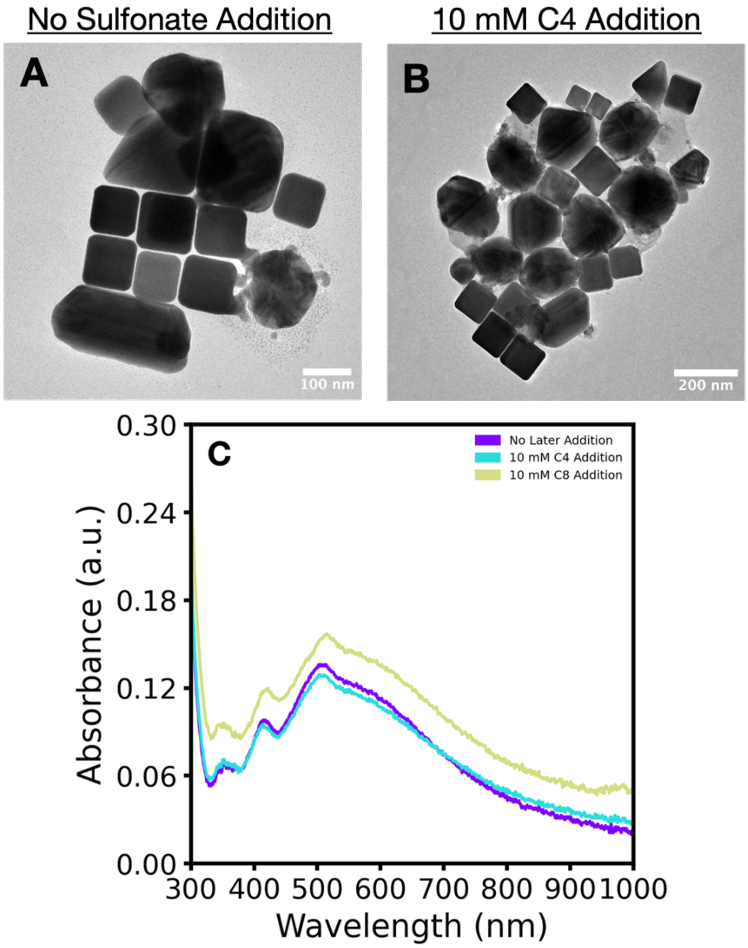

Figure S34. Transmission electron micrographs and absorbance spectra of purified nanocubes from Zhou et. al.[3] synthesis to which water, 10 mM sodium 1-butanesulfonate or 10 mM sodium 1-octanesulfonate were added post-synthesis. **A)** Transmission electron micrograph of purified nanocubes to which 30 μL of water was added post-synthesis. **B)** Transmission electron micrograph of purified nanocubes to which 10 mM sodium 1-butanesulfonate was added post-synthesis. **C)** Absorbance spectra of purified nanocubes with post-synthesis additions of water (no later sulfonate addition), 10 mM sodium 1-butanesulfonate (C4), or 10 mM sodium 1-octanesulfonate (C8). We could not image the samples in which sodium 1-octanesulfonate was added to the nanocubes because the grid was saturated with crystals, presumably due to the aggregation that aligns with the increase in scattering absorbance for that sample.

References

(1) Métraux, G. S.; Mirkin, C. A. Rapid Thermal Synthesis of Silver Nanoprisms with Chemically Tailorable Thickness. *Adv. Mater.* **2005**, *17* (4), 412–415. https://doi.org/10.1002/adma.200401086.

(2) Frank, A. J.; Cathcart, N.; Maly, K. E.; Kitaev, V. Synthesis of Silver Nanoprisms with Variable Size and Investigation of Their Optical Properties: A First-Year Undergraduate Experiment Exploring Plasmonic Nanoparticles. *J. Chem. Educ.* **2010**, *87* (10), 1098–1101. https://doi.org/10.1021/ed100166g.

(3) Zhou, S.; Li, J.; Gilroy, K. D.; Tao, J.; Zhu, C.; Yang, X.; Sun, X.; Xia, Y. Facile Synthesis of Silver Nanocubes with Sharp Corners and Edges in an Aqueous Solution. *ACS Nano* **2016**, *10* (11), 9861–9870. https://doi.org/10.1021/acsnano.6b05776.

(4) da Silva, R. M. P.; van der Zwaag, D.; Albertazzi, L.; Lee, S. S.; Meijer, E. W.; Stupp, S. I. Super-Resolution Microscopy Reveals Structural Diversity in Molecular Exchange among Peptide Amphiphile Nanofibres. *Nat. Commun.* **2016**, *7* (1), 11561. https://doi.org/10.1038/ncomms11561.

(5) Frank, A. J.; Cathcart, N.; Maly, K. E.; Kitaev, V. Synthesis of Silver Nanoprisms with Variable Size and Investigation of Their Optical Properties: A First-Year Undergraduate Experiment Exploring Plasmonic Nanoparticles. *J. Chem. Educ.* **2010**, *87* (10), 1098–1101. https://doi.org/10.1021/ed100166g.

(6) Martin, K. N.; Rubsamen, M. S.; Kaplan, N. P.; Hendricks, M. P. Method for Interfacing a Plate Reader Spectrometer Directly with an OT-2 Liquid Handling Robot. **2022**. https://doi.org/10.26434/chemrxiv-2022-6z4q1.

(7) Annunziata, O.; Costantino, L.; D'Errico, G.; Paduano, L.; Vitagliano, V. Transport Properties for Aqueous Sodium Sulfonate Surfactants: 2. Intradiffusion Measurements: Influence of the Obstruction Effect on the Monomer and Micelle Mobilities. *J. Colloid Interface Sci.* **1999**, *216* (1), 16–24. https://doi.org/10.1006/jcis.1999.6269.

(8) Mukerjee, P.; Mysels, Karol J. *Critical Micelle Concentrations of Aqueous Surfactant Systems*; U.S. National Bureau of Standards; Vol. 36.

(9) Stuart, M. C. A.; van de Pas, J. C.; Engberts, J. B. F. The Use of Nile Red to Monitor the Aggregation Behavior in Ternary Surfactant–Water–Organic Solvent Systems. *J. Phys. Org. Chem.* **2005**, *18* (9), 929–934. https://doi.org/10.1002/poc.919.

(10) Wu, M.; Vartanian, A. M.; Chong, G.; Pandiakumar, A. K.; Hamers, R. J.; Hernandez, R.; Murphy, C. J. Solution NMR Analysis of Ligand Environment in Quaternary Ammonium-Terminated Self-Assembled Monolayers on Gold Nanoparticles: The Effect of Surface Curvature and Ligand Structure. *J. Am. Chem. Soc.* **2019**, *141* (10), 4316–4327. https://doi.org/10.1021/jacs.8b11445.

(11) De Roo, J.; Yazdani, N.; Drijvers, E.; Lauria, A.; Maes, J.; Owen, J. S.; Van Driessche, I.; Niederberger, M.; Wood, V.; Martins, J. C.; Infante, I.; Hens, Z. Probing Solvent–Ligand Interactions in Colloidal Nanocrystals by the NMR Line Broadening. *Chem. Mater.* **2018**, *30* (15), 5485–5492. https://doi.org/10.1021/acs.chemmater.8b02523.

(12) Greskovich, K. M.; Powderly, K. M.; Kincanon, M. M.; Forney, N. B.; Jalomo, C. A.; Wo, A.; Murphy, C. J. The Landscape of Gold Nanocrystal Surface Chemistry. *Acc. Chem. Res.* **2023**, *56* (12), 1553–1564. https://doi.org/10.1021/acs.accounts.3c00109.

(13) Nuzzo, R. G.; Allara, D. L. Adsorption of Bifunctional Organic Disulfides on Gold Surfaces. *J. Am. Chem. Soc.* **1983**, *105* (13), 4481–4483. https://doi.org/10.1021/ja00351a063.
(14) Xue, Y.; Li, X.; Li, H.; Zhang, W. Quantifying Thiol–Gold Interactions towards the Efficient Strength Control. *Nat. Commun.* **2014**, *5* (1), 4348. https://doi.org/10.1038/ncomms5348.
